# Supplementary material for: Eco-alternative treatments for Vibrio parahaemolyticus and V. cholerae biofilms from shrimp industry through Eucalyptus (Eucalyptus globulus) and Guava (Psidium guajava) extracts: A road for an Ecuadorian sustainable economy
Source: PLoS One. 2024 Aug 13;19(8):e0304126. doi: 10.1371/journal.pone.0304126 (PMC11321589; doi:10.1371/journal.pone.0304126)
Supplement: S1 Fig — (DOCX) [file pone.0304126.s001.docx]

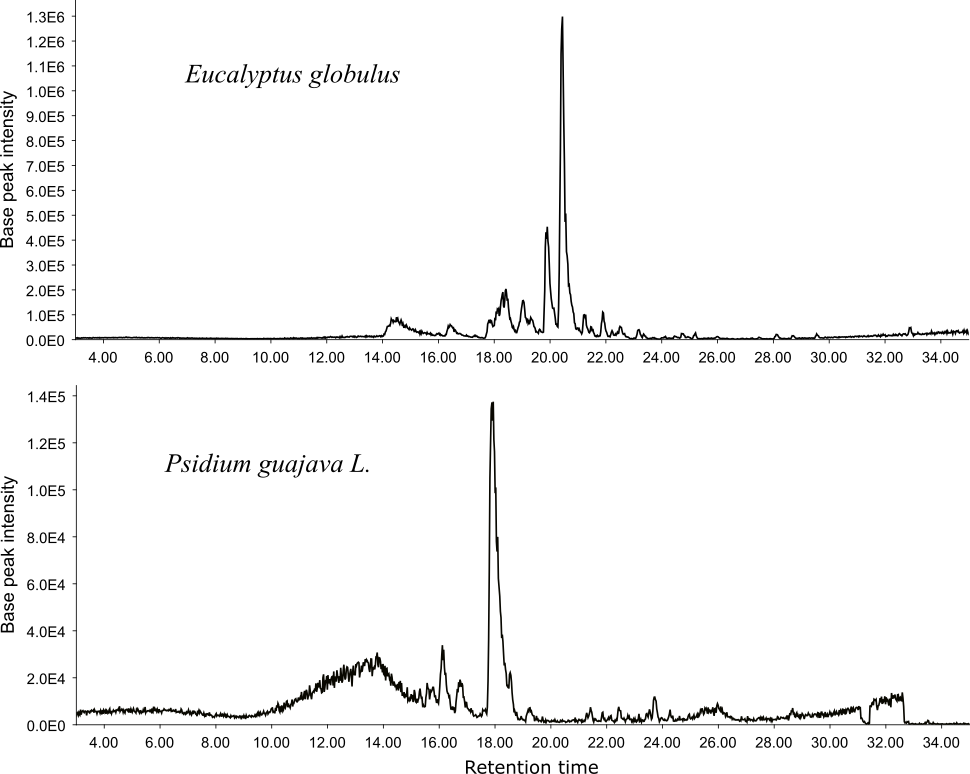


**S1 Fig. Descriptive chromatograms obtained in Eucalyptus (*Eucalyptus globulus* Labill.) and Guava (*Psidium guajava* L.) extracts during HPLC-DAD-MS analysis.**
