# Supplementary material for: Eco-alternative treatments for Vibrio parahaemolyticus and V. cholerae biofilms from shrimp industry through Eucalyptus (Eucalyptus globulus) and Guava (Psidium guajava) extracts: A road for an Ecuadorian sustainable economy
Source: PLoS One. 2024 Aug 13;19(8):e0304126. doi: 10.1371/journal.pone.0304126 (PMC11321589; doi:10.1371/journal.pone.0304126)
Supplement: S2 Fig — Biofilms of Vibrio parahaemolyticus (VP) illustrated in the best growth conditions, (at (24°C), (24h), and initial inoculum of 0.5 McFarland) by fluorescence microscopy using Live/Dead Invitrogen staining. The original image was zoomed at 1:10 to observe the cells in the biofilm to compare the total live and dead cells. An Olympus BX50 microscope with 100X magnification was used, images were obtained with AmScope software, and images were merged with Fiji-ImageJ software. (DOCX) [file pone.0304126.s002.docx]

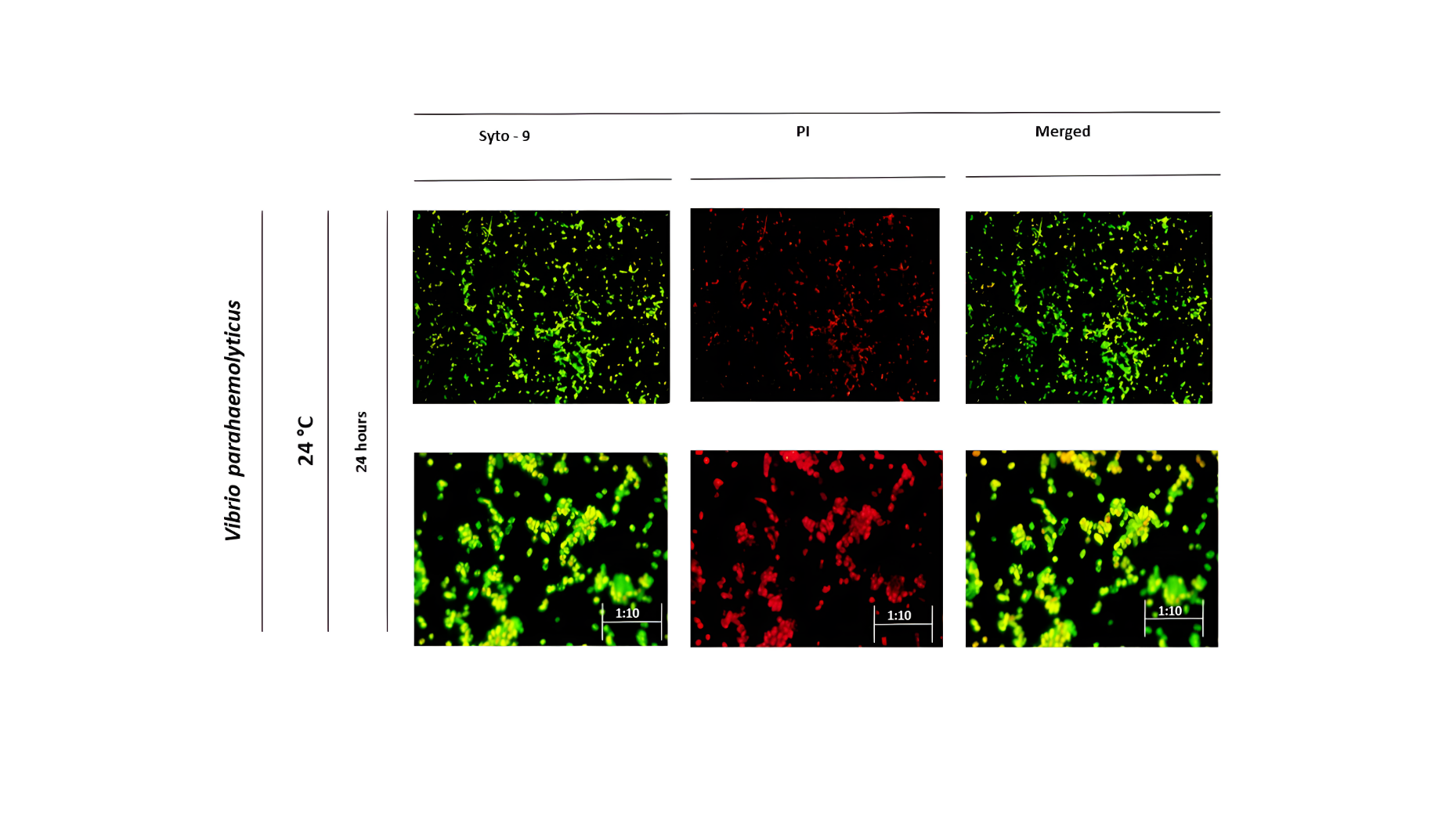


**S2 Fig. Fluorescence microscopy of the best conditions in biofilm formation by *V. parahaemolyticus* and *V. cholerae*.**

Biofilms of *Vibrio parahaemolyticus* (VP) illustrated in the best growth conditions, (at (24°C), (24h), and initial inoculum of 0.5 McFarland) by fluorescence microscopy using Live/Dead Invitrogen staining. The original image was zoomed at 1:10 to observe the cells in the biofilm to compare the total live and dead cells. An Olympus BX50 microscope with 100X magnification was used, images were obtained with AmScope software, and images were merged with Fiji-ImageJ software.
