# Supplementary material for: Eco-alternative treatments for Vibrio parahaemolyticus and V. cholerae biofilms from shrimp industry through Eucalyptus (Eucalyptus globulus) and Guava (Psidium guajava) extracts: A road for an Ecuadorian sustainable economy
Source: PLoS One. 2024 Aug 13;19(8):e0304126. doi: 10.1371/journal.pone.0304126 (PMC11321589; doi:10.1371/journal.pone.0304126)
Supplement: S1 Table — (DOCX) [file pone.0304126.s003.docx]

**S1 Table. Parameters created by desirability functions.**

| Response | Goal | Lower | Target | Upper | Weight | Import |
| --- | --- | --- | --- | --- | --- | --- |
| Biomass and Viability | | | | | | |
| Total cells | Maximum | 1.73E+04 | 8.48E+05 | 8.48E+05 | 1 | 1 |
| PBS | Maximum | 0.049 | 0.102 | 0.102 | 1 | 1 |
| CFU/mL | Maximum | 5.80E+06 | 2.80E+08 | 2.80E+08 | 1 | 1 |
| Minimum inhibitory concentration (MIC) | | | | | | |
| PBS | Minimum | 0.024 | 0.024 | 3.616 | 1 | 1 |
| Biofilm inhibition assays | | | | | | |
| PBS | Minimum | 0.049 | 0.049 | 3.012 | 1 | 1 |
| CFU/mL | Minimum | 1.00E+07 | 1.00E+07 | 2.53E+08 | 1 | 1 |
| Biofilm eradication assays | | | | | | |
| PBS | Minimum | 0.131 | 0.131 | 3.09 | 1 | 1 |
| CFU/mL | Minimum | 1.00E+07 | 1.00E+07 | 2.54E+08 | 1 | 1 |
