# Supplementary material for: Eco-alternative treatments for Vibrio parahaemolyticus and V. cholerae biofilms from shrimp industry through Eucalyptus (Eucalyptus globulus) and Guava (Psidium guajava) extracts: A road for an Ecuadorian sustainable economy
Source: PLoS One. 2024 Aug 13;19(8):e0304126. doi: 10.1371/journal.pone.0304126 (PMC11321589; doi:10.1371/journal.pone.0304126)
Supplement: S2 Table — The Minitab statistical program calculates an optimal solution that serves as the starting point for the plot, and the settings can be modified interactively to determine how different settings affect Vibrio biofilm growth responses using composite desirability is the weighted geometric mean of the individual desirability for the responses. Minitab determines optimal settings for the variables by maximizing the composite desirability. For biofilm formation, we sought to maximize the response of the variables with higher composite desirability. (DOCX) [file pone.0304126.s004.docx]

**S2 Table. Optimization of the growth conditions of the *Vibrio* species.**

| Biomass and viability optimization | | | | | | | | |
| --- | --- | --- | --- | --- | --- | --- | --- | --- |
| Condition | Species | Time | Temperature | Initial inoculum | Total cells adjusted | Biomass PBS abs 630 nm adjusted | Cell Viability CFU Log/mL adjusted | Composite desirability |
| *Vibrio* *parahaemolyticus* | | | | | | | | |
| 1 | VP | 24 | 24 | 0.5 | 1.79E+05 | 0.09 | 9.01E+07 | 0.35 |
| 2 | VP | 24 | 24 | 0.05 | 1.28E+05 | 0.09 | 9.96E+07 | 0.32 |
| 3 | VP | 30 | 72 | 0.5 | 3.45E+05 | 0.07 | 3.47E+07 | 0.26 |
| 4 | VP | 24 | 72 | 0.5 | 5.34E+04 | 0.08 | 1.57E+08 | 0.25 |
| 5 | VP | 24 | 72 | 0.05 | 6.09E+04 | 0.09 | 8.01E+07 | 0.22 |
| *Vibrio* *cholerae* | | | | | | | | |
| 1 | VC | 30 | 72 | 0.5 | 3.06E+05 | 0.09 | 1.49E+08 | 0.51 |
| 2 | VC | 30 | 72 | 0.05 | 4.40E+05 | 0.08 | 8.45E+07 | 0.44 |
| 3 | VC | 24 | 24 | 0.5 | 2.67E+05 | 0.07 | 3.83E+07 | 0.23 |
| 4 | VC | 24 | 24 | 0.05 | 1.37E+05 | 0.07 | 5.49E+07 | 0.21 |
| 5 | VC | 30 | 24 | 0.5 | 4.48E+04 | 0.07 | 1.22E+08 | 0.17 |

Legend- The Minitab statistical program calculates an optimal solution that serves as the starting point for the plot, and the settings can be modified interactively to determine how different settings affect *Vibrio* biofilm growth responses using composite desirability is the weighted geometric mean of the individual desirability for the responses. Minitab determines optimal settings for the variables by maximizing the composite desirability. For biofilm formation, we sought to maximize the response of the variables with higher composite desirability.
