# Supplementary material for: Eco-alternative treatments for Vibrio parahaemolyticus and V. cholerae biofilms from shrimp industry through Eucalyptus (Eucalyptus globulus) and Guava (Psidium guajava) extracts: A road for an Ecuadorian sustainable economy
Source: PLoS One. 2024 Aug 13;19(8):e0304126. doi: 10.1371/journal.pone.0304126 (PMC11321589; doi:10.1371/journal.pone.0304126)
Supplement: S3 Table — Evaluation of the in vitro biofilm formation of two Vibrio species (Vibrio parahaemolyticus and Vibrio cholerae). At least six assays with quintuplicate biofilms samples were performed on different days. For the evaluation of the data, normality, and data transformation tests were performed in order to obtain a parametric analysis of all the data with the Minitab program. The mean, standard deviation, and minimum and maximum range of the trials are shown in the table. All OD measurements by PBS suspension and CV staining were adjusted by subtracting the absorbance measurements of sterility controls from the absorbance measurements of biofilm samples. (DOCX) [file pone.0304126.s005.docx]

**S3 Table. Summary of the mean and median results of the present study from *Vibrio parahaemolyticus* and *Vibrio cholerae* biomass, cell viability, and total cells count assays.**

| **Species** | | | | ***Vibrio parahaemolyticus*** | | | | | | ***Vibrio cholerae*** | | | | | | |  | | ***Vibrio parahaemolyticus*** | | | ***Vibrio cholerae*** | | |
| --- | --- | --- | --- | --- | --- | --- | --- | --- | --- | --- | --- | --- | --- | --- | --- | --- | --- | --- | --- | --- | --- | --- | --- | --- |
| **Measurement techniques** | | | | **Biomass PBS A630** | | **Biomass CV A630** | | **Viability CFU Log/mL** | | **Biomass PBS A630** | | **Biomass CV A630** | | **Cell Viability CFU Log/mL** | | |  | | **Fluorescence Microscopy** | | | | | |
| **Time** | **Temperature** | **Initial Inoculum (McFarland)** | **Assays n°** | **Mean (SD)** | **Median [Min - Max]** | **Mean (SD)** | **Median [Min - Max]** | **Mean (SD)** | **Median [Min - Max]** | **Mean (SD)** | **Median [Min - Max]** | **Mean (SD)** | **Median [Min - Max]** | | **Mean (SD)** | **Median [Min - Max]** | | **Total samples** | **Total mean**  **cm ^2^**  **(SD)** | **Live mean**  **cm ^2^**  **(SD)** | **Dead mean**  **cm ^2^**  **(SD)** | **Total mean**  **cm ^2^**  **(SD)** | **Live mean**  **cm ^2^**  **(SD)** | **Dead mean**  **cm ^2^**  **(SD)** |
| 24h | 24°C | 0.05 | 6 | 0.09 (0.001) | 0.09 (0.09 - 0.09) | 0.20 (0.05) | 0.18 (0.15 - 0.26) | 8.04 (0.03) | 7.99 (7.9 - 8.36) | 0.07 (0.01) | 0.07 (0.06 - 0.07) | 0.10 (0.01) | 0.09 (0.09 - 0.11) | | 7.74 (0.11) | 7.73 (7.61 - 7.88) | | 15 | 9.29E+6  (8.97E+6) | 5.35E+6  (4.63E+6) | 3.94E+6  (4.41E+6) | 7.15E+6  (1.83E+7) | 5.53E+6  (3.95E+6) | 1.62E+6  (1.52E+6) |
|  |  | 0.5 | 6 | 0.09 (0.41) | 0.09 (0.08 - 0.09) | 0.16 (0.03) | 0.16 (0.123 - 0.201) | 7.96 (0.001) | 7.95 (7.91 - 8.00) | 0.07 (0.001) | 0.07 (0.06 - 0.07) | 0.10 (0.01) | 0.09 (0.09 - 0.10) | | 7.58 (0.09) | 7.59 (7.43 - 7.68) | | 15 | 1.51E+7  (1.38E+7) | 1.42E+7  (1.34E+7) | 8.88E+5  (9.01E+5) | 1.51E+7  (1.31E+6) | 1.37E+7  (1.35E+6) | 1.41E+6  (1.01E+6) |
|  | 30°C | 0.05 | 6 | 0.09 (0.01) | 0.09 (0.08 -0.10) | 0.10 (0.01) | 0.098 (0.095 - 0.112) | 7.60 (0.18) | 7.58 (7.46 - 7.75) | 0.07 (0.001) | 0.07 (0.07 - 0.08) | 0.08 (0.001) | 0.08 (0.08 - 0.08) | | 8.14 (0.24) | 8.02 (7.94 - 8.45) | | 15 | 3.92E+6  (2.73E+6) | 2.41E+6  (1.53E+6) | 1.51E+6  (1.31E+6) | 3.92E+6  (1.91E+6) | 3.45E+6  (1.99E+6) | 4.03E+5  (1.85E+5) |
|  |  | 0.5 | 6 | 0.07 (0.001) | 0.07 (0.07 - 0.08) | 0.09 (0.001) | 0.089 (0.082 - 0.089) | 7.51 (0.14) | 7.456 (7.38 - 7.69) | 0.07 (0.001) | 0.07 (0.06 - 0.07) | 0.07 (0.001) | 0.07 (0.07 - 0.07) | | 8.08 (0.11) | 8.10 (7.95 - 8.23) | | 15 | 1.35E+7  (1.42E+7) | 1.22E+7  (1.34E+7) | 1.33E+6  (1.28E+6) | 1.13E+7  (1.18E+7) | 9.29E+6  (9.07E+6) | 2.05E+6  (2.44E+6) |
| 48h | 24°C | 0.05 | 6 | 0.06 (0.001) | 0.06 (0.05 - 0.06) | 0.09 (0.01) | 0.918 (0.079 - 0.097) | 8.00 (0.06) | 8.01 (7.92 - 8.07) | 0.07 (0.01) | 0.07 (0.06 - 0.09) | 0.09 (0.01) | 0.09 (0.08 - 0.09) | | 7.87 (0.15) | 7.85 (7.70 - 8.07) | | 15 | 4.83E+6  (1.51E+6) | 3.53E+6  (1.09E+6) | 1.30E+6  (4.84E+5) | 3.67E+6  (1.84E+6) | 2.14E+6  (1.24E+6) | 1.53E+6  (7.21E+5) |
|  |  | 0.5 | 6 | 0.05 (0.01) | 0.05 (0.05 - 0.05) | 0.08 (0.001) | 0.077 (0.077 - 0.078) | 7.95 (0.05) | 7.93 (7.90 - 8.05) | 0.07 (0.01) | 0.07 (0.06 - 0.08) | 0.08 (0.01) | 0.08 (0.07 - 0.08) | | 7.57 (0.11) | 7.56 (7.45 - 7.73) | | 15 | 3.72E+6  (2.04E+6) | 3.39E+6  (1.90E+6) | 3.23E+5  (3.37E+5) | 2.95E+6  (9.24E+5) | 2.62E+6  (9.29E+5) | 3.33E+5  (3.43E+5) |
|  | 30°C | 0.05 | 6 | 0.05 (0.001) | 0.05 (0.05 - 0.06) | 0.10 (0.01) | 0.096 (0.091 - 0.104) | 7.32 (0.03) | 7.36 (7.04 - 7.53) | 0.06 (0.001) | 0.06 (0.05 - 0.06) | 0.09 (0.02) | 0.08 (0.08 - 0.12) | | 7.66 (0.08) | 7.64 (7.59 - 7.76) | | 15 | 2.70E+6  (2.12E+6) | 1.69E+6  (1.52E+6) | 1.01E+6  (6.46E+5) | 3.79E+6  (2.16E+6) | 2.13E+6  (1.37E+6) | 1.66E+6  (8.86E+5) |
|  |  | 0.5 | 6 | 0.05 (0.001) | 0.05 (0.05 - 0.05) | 0.08 (0.001) | 0.078 (0.076 - 0.078) | 7.27 (0.12) | 7.28 (7.08 - 7.43) | 0.06 (0.001) | 0.06 (0.06 - 0.07) | 0.08 (0.001) | 0.08 (0.07 - 0.08) | | 7.64 (0.16) | 7.70 (7.42 - 7.84) | | 15 | 2.78E+6  (1.54E+6) | 2.12E+6  (1.44E+6) | 6.60E+5  (5.98E+5) | 3.19E+6  (1.54E+6) | 2.93E+6  (1.56E+6) | 2.56E+5  (3.37E+5) |
| 72h | 24°C | 0.05 | 6 | 0.09 (0.14) | 0.09 (0.07 - 0.10) | 0.09 (0.001) | 0.090 (0.090 - 0.178) | 7.90 (0.04) | 7.90 (7.85 - 7.96) | 0.05 (0.001) | 0.05 (0.04 - 0.05) | 0.07 (0.01) | 0.07 (0.07 - 0.07) | | 7.38 (0.39) | 7.56 (6.76 - 7.69) | | 15 | 4.31E+6  (3.15E+6) | 2.63E+6  (2.11E+6) | 1.66E+6  (1.11E+6) | 5.52E+6  (3.58E+6) | 4.58E+6  (3.56E+6) | 9.43E+5  (5.94E+5) |
|  |  | 0.5 | 6 | 0.08 (0.001) | 0.08 (0.08 – 0.09) | 0.11 (0.03) | 0.097 (0.090 - 0.178) | 8.19 (0.21) | 8.21 (7.96 - 8.42) | 0.07 (0.001) | 0.07 (0.06 - 0.08) | 0.07 (0.01) | 0.06 (0.06 - 0.07) | | 7.47 (0.13) | 7.47 (7.32 - 7.62) | | 15 | 7.21E+6  (8.96E+6) | 6.21E+6  (8.30E+6) | 9.95E+5  (1.45E+6) | 2.71E+6  (2.03E+6) | 2.60E+6  (2.08E+6) | 1.25E+5  (1.21E+5) |
|  | 30°C | 0.05 | 6 | 0.07 (0.01) | 0.07 (0.06 - 0.07) | 0.09 (0.001) | 0.089 (0.087 - 0.095) | 7.24 (0.03) | 7.27 (7.03 - 7.41) | 0.08 (0.001) | 0.08 (0.08 - 0.09) | 0.11 (0.04) | 0.10 (0.09 - 0.19) | | 7.92 (0.05) | 7.93 (7.86 - 7.98) | | 15 | 5.43E+6  (2.47E+6) | 3.51E+6  (1.55E+6) | 1.91E+6  (1.07E+6) | 6.06E+6  (4.95E+6) | 5.56E+6  (4.81E+6) | 4.99E+5  (2.38E+5) |
|  |  | 0.5 | 6 | 0.07 (0.001) | 0.07 (0.07 - 0.08) | 0.09 (0.001) | 0.092 (0.089 - 0.093) | 7.54 (0.02) | 7.36 (7.36 - 7.73) | 0.09 (0.001) | 0.09 (0.08 - 0.09) | 0.12 (0.01) | 0.12 (0.11 - 0.13) | | 8.17 (0.04) | 8.17 (7.95 - 8.45) | | 15 | 1.28E+7  (5.93E+6) | 1.15E+7  (5.54E+6) | 1.36E+6  (5.75E+5) | 4.05E+6  (2.63E+6) | 3.84E+6  (2.51E+6) | 2.15E+5  (2.36E+5) |

Legend- Evaluation of the *in vitro* biofilm formation of two *Vibrio* species (*Vibrio* *parahaemolyticus* and *Vibrio* *cholerae*). At least six assays with quintuplicate biofilms samples were performed on different days. For the evaluation of the data, normality, and data transformation tests were performed in order to obtain a parametric analysis of all the data with the Minitab program. The mean, standard deviation, and minimum and maximum range of the trials are shown in the table. All OD measurements by PBS suspension and CV staining were adjusted by subtracting the absorbance measurements of sterility controls from the absorbance measurements of biofilm samples.
