# Supplementary material for: Eco-alternative treatments for Vibrio parahaemolyticus and V. cholerae biofilms from shrimp industry through Eucalyptus (Eucalyptus globulus) and Guava (Psidium guajava) extracts: A road for an Ecuadorian sustainable economy
Source: PLoS One. 2024 Aug 13;19(8):e0304126. doi: 10.1371/journal.pone.0304126 (PMC11321589; doi:10.1371/journal.pone.0304126)
Supplement: S4 Table — Variables, 1: Species; 2: Temperature; 3: Time; and 4: Initial Inoculum. A normalization test and data transformation were performed to obtain normalized data for a parametric test of biofilm growth. A multivariate ANOVA statistical analysis was performed to evaluate biofilm formation in TSB plus 1% NaCl differences between statistical values of multiple variables. The degrees of freedom (DF) indicate the number of observations free to vary, the adjusted sum of squares (SC) indicates the total sum of the variation or deviation contributed by the variables, the adjusted mean square (MC) indicates the variation that exists between the variables, the F value indicates the significance that a variable contributes to the system and the relationship between the variables (the higher the F value the greater the significance), and the p value equal to or less than 0.05 indicates that the null hypothesis is false and variables are significant for biofilm growth. (DOCX) [file pone.0304126.s006.docx]

**S4 Table. Summary of results of reducing model of ANOVA statistical analysis of biomass, viability, total cells counting assays in *Vibrio parahaemolyticus* and *Vibrio cholerae.***

| **ANOVA reduced model abstract** | | | | | | | | | | |
| --- | --- | --- | --- | --- | --- | --- | --- | --- | --- | --- |
|  | **Biomass CV A630** | | | | | **Biomass PBS A630** | | | | |
| **Variables** | **DF** | **SC adjusted** | **MC adjusted** | **F-Value** | **P-Value** | **DF** | **SC adjusted** | **MC adjusted** | **F-Value** | **P-Value** |
| **1** | 1.00 | 49793.00 | 49792.90 | 112.60 | 0.001 | 1.00 | 765.00 | 765.00 | 1.12 | 0.29 |
| **2** | 1.00 | 9.00 | 9.40 | 0.02 | 0.88 | 1.00 | 458.00 | 458.00 | 0.67 | 0.42 |
| **3** | 2.00 | 37248.00 | 18623.90 | 42.11 | 0.0001 | 2.00 | 394890.00 | 197445.00 | 288.31 | 0.001 |
| **4** | 1.00 | 15694.00 | 15693.90 | 35.49 | 0.001 | 1.00 | 13125.00 | 13125.00 | 19.17 | 0.001 |
| **1*2** | 1.00 | 30402.00 | 30402.10 | 68.75 | 0.001 | 1.00 | 59018.00 | 59018.00 | 86.18 | 0.001 |
| **1*3** | 2.00 | 16472.00 | 8235.80 | 18.62 | 0.001 | 2.00 | 238057.00 | 119029.00 | 173.81 | 0.001 |
| **1*4** | 1.00 | 137.00 | 137.10 | 0.31 | 0.58 | 1.00 | 23472.00 | 23472.00 | 34.27 | 0.001 |
| **2*3** | 2.00 | 99851.00 | 49925.50 | 112.90 | 0.001 | 2.00 | 49754.00 | 24877.00 | 36.33 | 0.001 |
| **2*4** | 1.00 | 527.00 | 527.30 | 1.19 | 0.28 | 1.00 | 923.00 | 923.00 | 1.35 | 0.25 |
| **3*4** | 2.00 | 18767.00 | 9383.30 | 21.22 | 0.001 | 2.00 | 45155.00 | 22577.00 | 32.97 | 0.001 |
| **1*2*3** | 2.00 | 45124.00 | 22561.80 | 51.02 | 0.001 | 2.00 | 134686.00 | 67343.00 | 98.33 | 0.001 |
| **1*2*4** | - | - | - | - | - | 1.00 | 6832.00 | 6832.00 | 9.98 | 0.001 |
| **1*3*4** | - | - | - | - | - | 2.00 | 12338.00 | 6169.00 | 9.01 | 0.001 |
| **2*3*4** | - | - | - | - | - | 2.00 | 26082.00 | 13041.00 | 19.04 | 0.0001 |
| **1*2*3*4** | - | - | - | - | - | 2.00 | 41229.00 | 20614.00 | 30.10 | 0.0001 |
| **Error** | 127.00 | 56162.00 | 442.20 | - | - | - | - | - | - | - |
| **Lack of fit** | 7.00 | 5523.00 | 789.00 | 1.87 | 0.08 | - | - | - | - | - |
| **Pure error** | 120.00 | 50640.00 | 422.00 | - | - | 120.00 | 82180.00 | 685.00 | - | - |
| **Total** | 143.00 | 370186.00 | - | - | - | 143.00 | 1128965.00 | - | - | - |
|  | **Viability CFU Log/mL** | | | | | **Live cells per cm^2^** | | | | |
| **Variables** | **DF** | **SC adjusted** | **MC adjusted** | **F-Value** | **P-Value** | **DF** | **SC adjusted** | **MC adjusted** | **F-Value** | **P-Value** |
| **1** | 1.00 | 0.12 | 0.12 | 6.12 | 0.02 | 1.00 | 0.02 | 0.02 | 0.02 | 0.88 |
| **2** | 1.00 | 0.63 | 0.63 | 31.14 | 0.001 | 1.00 | 1401.00 | 14009.00 | 1.54 | 0.22 |
| **3** | 2.00 | 0.60 | 0.30 | 14.72 | 0.001 | 2.00 | 57704.00 | 288522.00 | 31.70 | 0.001 |
| **4** | 1.00 | 0.01 | 0.01 | 0.58 | 0.45 | 1.00 | 22723.00 | 227234.00 | 24.97 | 0.001 |
| **1*2** | 1.00 | 74667.00 | 746672.00 | 368.64 | 0.0001 | 1.00 | 0.25 | 0.25 | 0.27 | 0.60 |
| **1*3** | 2.00 | 0.04 | 0.02 | 1.04 | 0.36 | 2.00 | 6749.00 | 33745.00 | 3.71 | 0.03 |
| **1*4** | 1.00 | 0.06 | 0.06 | 3.00 | 0.09 | 1.00 | 7290.00 | 72897.00 | 8.01 | 0.01 |
| **2*3** | 2.00 | 10657.00 | 0.53 | 26.31 | 0.001 | 2.00 | 38045.00 | 190226.00 | 20.90 | 0.0001 |
| **2*4** | 1.00 | 0.08 | 0.08 | 4.11 | 0.05 | 1.00 | 7315.00 | 73152.00 | 8.04 | 0.01 |
| **3*4** | 2.00 | 0.81 | 0.41 | 20.10 | 0.0001 | 2.00 | 2196.00 | 10979.00 | 1.21 | 0.30 |
| **1*2*3** | 2.00 | 0.69 | 0.34 | 16.93 | 0.001 | 2.00 | 16604.00 | 83018.00 | 9.12 | 0.001 |
| **1*2*4** | 1.00 | 0.09 | 0.09 | 4.60 | 0.03 | - | - | - | - | - |
| **1*3*4** | - | - | - | - | - | 2.00 | 16148.00 | 80738.00 | 8.87 | 0.0001 |
| **2*3*4** | - | - | - | - | - | - | - | - | - | - |
| **1*2*3*4** | - | - | - | - | - | - | - | - | - | - |
| **Error** | 126.00 | 25521.00 | 0.02 |  |  | 701.00 | 638033.00 | 0.91 |  |  |
| **Lack of fit** | 6.00 | 0.05 | 0.01 | 0.36 | 0.90 | 5.00 | 2488.00 | 0.50 | 0.54 | 0.74 |
| **Pure error** | 120.00 | 25066.00 | 0.02 | - | - | 696.00 | 635545.00 | 0.91 | - | - |
| **Total** | 143.00 | 142264.00 | - | - | - | 719.00 | 814369.00 | - | - | - |
|  | **Dead cells per cm^2^** | | | | | **Total cells per cm^2^** | | | | |
| **Variables** | **DF** | **SC adjusted** | **MC adjusted** | **F-Value** | **P-Value** | **DF** | **SC adjusted** | **MC adjusted** | **F-Value** | **P-Value** |
| **1** | 1.00 | 830.80 | 830.81 | 64.46 | 0.001 | 1.00 | 1818.00 | 18177.00 | 2.59 | 0.11 |
| **2** | 1.00 | 8.20 | 8.25 | 0.64 | 0.42 | 1.00 | 1699.00 | 16988.00 | 2.42 | 0.12 |
| **3** | 2.00 | 765.30 | 382.64 | 29.69 | 0.001 | 2.00 | 50120.00 | 250598.00 | 35.71 | 0.001 |
| **4** | 1.00 | 2419.80 | 2419.77 | 187.73 | 0.001 | 1.00 | 3696.00 | 36964.00 | 5.27 | 0.02 |
| **1*2** | 1.00 | 116.30 | 116.31 | 9.02 | 0.001 | 1.00 | 0.02 | 0.02 | 0.03 | 0.87 |
| **1*3** | 2.00 | 810.80 | 405.42 | 31.45 | 0.001 | 2.00 | 12317.00 | 61585.00 | 8.78 | 0.001 |
| **1*4** | 1.00 | 5.60 | 5.57 | 0.43 | 0.51 | 1.00 | 4844.00 | 48436.00 | 6.90 | 0.01 |
| **2*3** | 2.00 | 134.00 | 67.01 | 5.20 | 0.01 | 2.00 | 26037.00 | 130187.00 | 18.55 | 0.001 |
| **2*4** | 1.00 | 408.90 | 408.88 | 31.72 | 0.001 | 1.00 | 7601.00 | 76006.00 | 10.83 | 0.001 |
| **3*4** | 2.00 | 828.00 | 414.00 | 32.12 | 0.001 | 2.00 | 9025.00 | 45123.00 | 6.43 | 0.001 |
| **1*2*3** | 2.00 | 56.80 | 28.38 | 2.20 | 0.11 | 2.00 | 10246.00 | 51231.00 | 7.30 | 0.001 |
| **1*2*4** | 1.00 | 33.70 | 33.75 | 2.62 | 0.11 | - | - | - | - | - |
| **1*3*4** | 2.00 | 714.60 | 357.28 | 27.72 | 0.001 | 2.00 | 13492.00 | 67460.00 | 9.61 | 0.0001 |
| **2*3*4** | 2.00 | 133.20 | 66.61 | 5.17 | 0.01 | - | - | - | - | - |
| **1*2*3*4** | 2.00 | 100.20 | 50.08 | 3.89 | 0.02 | - | - | - | - | - |
| **Error** | 696.00 | 8971.30 | 12.89 |  |  | 701.00 | 491957.00 | 0.70 |  |  |
| **Lack of fit** | - | - | - | - | - | 5.00 | 2526.00 | 0.51 | 0.72 | 0.61 |
| **Pure error** | - | - | - | - | - | 696.00 | 489431.00 | 0.70 | - | - |
| **Total** | 719.00 | 16333.20 | - | - | - | 719.00 | 632666.00 | - | - | - |

Legend- Variables, 1: Species; 2: Temperature; 3: Time; and 4: Initial Inoculum. A normalization test and data transformation were performed to obtain normalized data for a parametric test of biofilm growth. A multivariate ANOVA statistical analysis was performed to evaluate biofilm formation in TSB plus 1% NaCl differences between statistical values of multiple variables. The degrees of freedom (DF) indicate the number of observations free to vary, the adjusted sum of squares (SC) indicates the total sum of the variation or deviation contributed by the variables, the adjusted mean square (MC) indicates the variation that exists between the variables, the F value indicates the significance that a variable contributes to the system and the relationship between the variables (the higher the F value the greater the significance), and the *p* value equal to or less than 0.05 indicates that the null hypothesis is false and variables are significant for biofilm growth.
