# Supplementary material for: Eco-alternative treatments for Vibrio parahaemolyticus and V. cholerae biofilms from shrimp industry through Eucalyptus (Eucalyptus globulus) and Guava (Psidium guajava) extracts: A road for an Ecuadorian sustainable economy
Source: PLoS One. 2024 Aug 13;19(8):e0304126. doi: 10.1371/journal.pone.0304126 (PMC11321589; doi:10.1371/journal.pone.0304126)
Supplement: S5 Table — The Tukey and Fisher methods of intervariable comparison create confidence intervals for all pairwise differences in the means of the levels of the variables. A p value less than or equal to 0.05 indicates whether there is a significant difference between the pairs of variable levels. (DOCX) [file pone.0304126.s007.docx]

**S5 Table. Summary of the statistical analysis and intervariable correlations by Tukey and Fisher methods in biofilm formation of *V. parahaemolyticus* (VP) and *V. cholerae* (VC).**

| **Tukey and Fisher Pot Variables Comparison Plot** | | | | | | | | | | | |
| --- | --- | --- | --- | --- | --- | --- | --- | --- | --- | --- | --- |
|  |  | **Biomass CV A630** | | | | | **Biomass PBS A630** | | | | |
| **Variables** |  | **Mean** | **Mean Difference** | **Significant difference** | | **P-Value** | **Mean** | **Mean Difference** | **Significant difference** | | **P-Value** |
| **Species** | **VP** | 0.10 | 0.01 | VP - VC | Yes | 0.001 | 0.08 | 0.00 | VP - VC | No | 0.20 |
|  | **VC** | 0.08 |  |  |  |  | 0.07 |  |  |  |  |
| **Temperature** | **24°C** | 0.09 | 0.00 | 24°C - 30°C | No | 0.99 | 0.07 | 0.00 | 24°C - 30°C | No | 0.51 |
|  | **30°C** | 0.09 |  |  |  |  | 0.07 |  |  |  |  |
| **Initial Inoculum** | **0.05** | 0.10 | 0.01 | 0.05 - 0.5 | No | 0.10 | 0.07 | 0.00 | 0.05 - 0.5 | No | 0.33 |
|  | **0.5** | 0.09 |  |  |  |  | 0.07 |  |  |  |  |
| **Time** | **24h** | 0.10 | 0.01 | 24h - 48h | Yes | 0.001 | 0.08 | 0.02 | 24h - 48h | Yes | 0.001 |
|  | **48h** | 0.08 |  | 48h - 72h | No | 0.18 | 0.06 | 0.01 | 48h - 72h | Yes | 0.001 |
|  | **72h** | 0.09 | 0.01 | 24h - 72h | Yes | 0.03 | 0.07 | 0.01 | 24h - 72h | No | 0.54 |
|  |  | **Viability CFU Log/mL** | | | | | **Live cells per cm^2^** | | | | |
| **Variables** |  | **Mean** | **Mean Difference** | **Significant difference** | | **P-Value** | **Mean** | **Mean Difference** | **Significant difference** | | **P-Value** |
| **Species** | **VP** | 7.72 | 0.06 | VP - VC | No | 0.26 | 2.98E+6 | 0.01 | VP - VC | No | 0.88 |
|  | **VC** | 7.77 |  |  |  |  | 2.95E+6 |  |  |  |  |
| **Temperature** | **24°C** | 7.81 | 0.13 | 24°C - 30°C | Yes | 0.02 | 3.10E+6 | 0.09 | 24°C - 30°C | No | 0.25 |
|  | **30°C** | 7.68 |  |  |  |  | 2.84E+6 |  |  |  |  |
| **Initial Inoculum** | **0.05** | 7.74 | 0.02 | 0.05 - 0.5 | No | 0.86 | 3.54E+6 | 0.36 | 0.05 - 0.5 | Yes | 0.001 |
|  | **0.5** | 7.75 |  |  |  |  | 2.48E+6 |  |  |  |  |
| **Time** | **24h** | 7.83 | 0.16 | 24h - 48h | Yes | 0.02 | 3.97E+6 | 0.68 | 24h - 48h | Yes | 0.001 |
|  | **48h** | 7.67 | 0.07 | 48h - 72h | No | 0.52 | 3.25E+06 | 0.48 | 48h - 72h | Yes | 0.001 |
|  | **72h** | 7.74 | 0.09 | 24h - 72h | No | 0.26 | 2.02E+06 | 0.20 | 24h - 72h | Yes | 0.03 |
|  |  | **Dead cells per** **cm^2^** | | | | | **Total cells per cm^2^** | | | | |
| **Variables** |  | **Mean** | **Mean Difference** | **Significant difference** | | **P-Value** | **Mean** | **Mean Difference** | **Significant difference** | | **P-Value** |
| **Species** | **VP** | 9.09E+5 | 2.15 | VP - VC | Yes | 0.001 | 4.26E+06 | 0.10 | VP - VC | No | 0.13 |
|  | **VC** | 5.05E+5 |  |  |  |  | 3.86E+06 |  |  |  |  |
| **Temperature** | **24°C** | 7.04E+05 | 0.21 | 24°C - 30°C | No | 0.41 | 4.26E+06 | 0.10 | 24°C - 30°C | No | 0.15 |
|  | **30°C** | 6.64E+05 |  |  |  |  | 3.86E+06 |  |  |  |  |
| **Initial Inoculum** | **0.05** | 1.10E+06 | 3.67 | 0.05 - 0.5 | Yes | 0.001 | 4.36E+06 | 0.14 | 0.05 - 0.5 | Yes | 0.03 |
|  | **0.5** | 4.03E+05 |  |  |  |  | 3.77E+06 |  |  |  |  |
| **Time** | **24h** | 1.00E+06 | 2.24 | 24h - 48h | No | 0.93 | 5.51E+06 | 0.64 | 24h - 48h | Yes | 0.001 |
|  | **48h** | 5.68E+05 | 0.12 | 48h - 72h | Yes | 0.001 | 2.89E+06 | 0.37 | 48h - 72h | Yes | 0.001 |
|  | **72h** | 5.49E+05 | 2.12 | 24h - 72h | No | 0.05 | 4.18E+06 | 0.28 | 24h - 72h | Yes | 0.001 |

Legend- The Tukey and Fisher methods of intervariable comparison create confidence intervals for all pairwise differences in the means of the levels of the variables. A *p* value less than or equal to 0.05 indicates whether there is a significant difference between the pairs of variable levels.
