# Supplementary material for: Eco-alternative treatments for Vibrio parahaemolyticus and V. cholerae biofilms from shrimp industry through Eucalyptus (Eucalyptus globulus) and Guava (Psidium guajava) extracts: A road for an Ecuadorian sustainable economy
Source: PLoS One. 2024 Aug 13;19(8):e0304126. doi: 10.1371/journal.pone.0304126 (PMC11321589; doi:10.1371/journal.pone.0304126)
Supplement: S6 Table — (DOCX) [file pone.0304126.s008.docx]

**S6 Table. Summary table of Minimum Inhibition Concentration (MIC).**

| **Minimum inhibitory concentration (MIC)** | | | | | | | | | | | | | | | | | | |
| --- | --- | --- | --- | --- | --- | --- | --- | --- | --- | --- | --- | --- | --- | --- | --- | --- | --- | --- |
| **Microorganism** | | | | | **Control** | | | | | | | | | | | | | |
| ***Vibrio parahaemolyticus* (VP-87)** | | | | | **Abs 630 (SD)** | | | | | | | 2.93  (0.19) | | | | | | |
|  |  |  |  |  | **% Inhibition (%SD)** | | | | | | | -  (6.34) | | | | | | |
| ***Vibrio parahaemolyticus* (VP-275)** | | | | | **Abs 630 (SD)** | | | | | | | 2.87  (0.28) | | | | | | |
|  |  |  |  |  | **% Inhibition (%SD)** | | | | | | | -  (9.94) | | | | | | |
| ***Vibrio cholerae* (VC-112)** | | | | | **Abs 630 (SD)** | | | | | | | 1.39  (0.17) | | | | | | |
|  |  |  |  |  | **% Inhibition (%SD)** | | | | | | | -  (12.12) | | | | | | |
| **Plant Extract** | | | | | | | | | | | | | | | | | | |
| **Microorganism** | **Concentration (μg/ml)** | | **50** | **100** | | **200** | **400** | **800** | **1600** | **Concentration (μg/ml)** | | | **50** | **100** | **200** | **400** | **800** | **1600** |
| ***Vibrio parahaemolyticus* (VP-87)** | **Eucalyptus** | **Abs 630 (SD)** | 1.78 | 1.56 | | 0.44 | 0.38 | 0.40 | 0.43 | **Guava** | **Abs 630 (SD)** | | 2.60 | 2.24 | 2.09 | 1.76 | 0.64 | 0.75 |
|  |  |  | (0.07) | (0.03) | | (0.05) | (0.04) | (0.05) | (0.05) |  |  |  | (0.14) | (0.04) | (0.06) | (0.03) | (0.04) | (0.03) |
|  |  | **% Inhibition (%SD)** | 39.04 | 46.74 | | 85.04 | 87.11 | 86.45 | 85.24 |  | **% Inhibition (%SD)** | | 11.00 | 23.42 | 28.39 | 39.82 | 78.20 | 74.38 |
|  |  |  | (3.85) | (2.14) | | (11.89) | (10.24) | (13.65) | (10.91) |  |  |  | (5.45) | (1.71) | (2.75) | (1.85) | (5.65) | (3.60) |
| ***Vibrio parahaemolyticus* (VP-275)** |  | **Abs 630 (SD)** | 1.78 | 1.37 | | 0.45 | 0.51 | 0.13 | 0.16 |  | **Abs 630 (SD)** | | 2.81 | 2.63 | 1.83 | 0.62 | 0.56 | 0.55 |
|  |  |  | (0.09) | (0.05) | | (0.04) | (0.04) | (0.01) | (0.01) |  |  |  | (0.05) | (0.06) | (0.06) | (0.04) | (0.02) | (0.08) |
|  |  | **% Inhibition (%SD)** | 37.89 | 52.16 | | 84.14 | 82.04 | 95.61 | 94.33 |  | **% Inhibition (%SD)** | | 2.02 | 8.1 | 36.30 | 78.27 | 80.44 | 80.82 |
|  |  |  | (5.13) | (3.52) | | (9.01) | (8.64) | (9.93) | (7.31) |  |  |  | (1.89) | (2.16) | (3.31) | (6.81) | (3.73) | (14.29) |
| ***Vibrio cholerae* (VC-112)** |  | **Abs 630 (SD)** | 1.09 | 0.91 | | 0.54 | 0.53 | 0.55 | 0.54 |  | **Abs 630 (SD)** | | 1.27 | 1.19 | 1.07 | 0.98 | 0.65 | 0.75 |
|  |  |  | (0.05) | (0.04) | | (0.02 | (0.01) | (0.02) | (0.01) |  |  |  | (0.02) | (0.03) | (0.04) | (0.04) | (0.02) | (0.06) |
|  |  | **% Inhibition (%SD)** | 21.38 | 34.81 | | 61.28 | 62.22 | 60.80 | 61.10 |  | **% Inhibition (%SD)** | | 8.34 | 14.26 | 23.32 | 29.60 | 52.95 | 46.24 |
|  |  |  | (4.28) | (4.56) | | (3.41) | (2.70) | (3.15) | (2.66) |  |  |  | (1.24) | (2.29) | (3.50) | (4.46) | (2.72) | (7.36) |
| **Antibiotics** | | | | | | | | | | | | | | | | | | |
| **Microorganism** | **Concentration (μg/ml)** | | **2** | **5** | | **10** | **20** | **40** | **80** | **Concentration (μg/ml)** | | | **2** | **5** | **10** | **20** | **40** | **80** |
| ***Vibrio parahaemolyticus* (VP-87)** | **Tetracycline** | **Abs 630 (SD)** | 0.63 | 0.32 | | 0.05 | 0.04 | 0.05 | 0.06 | **Ceftriaxone** | **Abs 630**  **SD** | | 0.08 | 0.06 | 0.07 | 0.07 | 0.07 | 0.07 |
|  |  |  | (0.027) | (0.023) | | (0.002) | (0.003) | (0.003) | (0.003) |  |  |  | 0.004 | 0.003 | 0.005 | 0.005 | 0.004 | 0.004 |
|  |  | **% Inhibition (%SD)** | 78.47 | 89.13 | | 98.25 | 98.49 | 98.15 | 97.78 |  | **% Inhibition %SD** | | 97.28 | 97.99 | 97.60 | 97.68 | 97.63 | 97.61 |
|  |  |  | (4.37) | (7.20) | | (4.11) | (7.61) | (6.04) | (4.06) |  |  |  | 5.61 | 5.79 | 7.82 | 7.54 | 6.42 | 6.03 |
| ***Vibrio parahaemolyticus* (VP-275)** |  | **Abs 630 (SD)** | 1.95 | 1.77 | | 1.75 | 1.70 | 1.61 | 1.62 |  | **Abs 630**  **SD** | | 0.08 | 0.08 | 0.08 | 0.08 | 0.07 | 0.07 |
|  |  |  | (0.13) | (0.14) | | (0.13) | (0.12) | (0.08) | (0.12) |  |  |  | 0.006 | 0.006 | 0.006 | 0.006 | 0.005 | 0.004 |
|  |  | **% Inhibition (%SD)** | 32.12 | 38.34 | | 38.97 | 40.70 | 43.72 | 43.47 |  | **% Inhibition %SD** | | 97.22 | 97.07 | 97.05 | 97.34 | 97.46 | 97.59 |
|  |  |  | (6.48) | (8.02) | | (7.46) | (6.85) | (4.86) | (7.66) |  |  |  | 7.71 | 6.97 | 7.30 | 7.46 | 6.68 | 6.12 |
| ***Vibrio cholerae* (VC-112)** |  | **Abs 630 (SD)** | 0.38 | 0.05 | | 0.06 | 0.06 | 0.07 | 0.05 |  | **Abs 630**  **SD** | | 0.09 | 0.09 | 0.08 | 0.08 | 0.07 | 0.07 |
|  |  |  | (0.035) | (0.004) | | (0.004) | (0.005) | (0.002) | (0.004) |  |  |  | 0.005 | 0.006 | 0.004 | 0.006 | 0.004 | 0.003 |
|  |  | **% Inhibition (%SD)** | 72.79 | 96.68 | | 95.96 | 95.51 | 95.23 | 96.48 |  | **% Inhibition %SD** | | 93.24 | 93.51 | 94.12 | 94.29 | 94.74 | 95.19 |
|  |  |  | (9.35) | (8.35) | | (7.51) | (7.41) | (2.31) | (7.77) |  |  |  | 4.92 | 7.18 | 4.44 | 7.09 | 4.86 | 4.93 |
