# Supplementary material for: Eco-alternative treatments for Vibrio parahaemolyticus and V. cholerae biofilms from shrimp industry through Eucalyptus (Eucalyptus globulus) and Guava (Psidium guajava) extracts: A road for an Ecuadorian sustainable economy
Source: PLoS One. 2024 Aug 13;19(8):e0304126. doi: 10.1371/journal.pone.0304126 (PMC11321589; doi:10.1371/journal.pone.0304126)
Supplement: S7 Table — Summary table with the absorbance values at 630 nm, CFU, percentage of inhibition and eradication, and their respective standard deviation values obtained in the Biofilm tests carried out on 2 species of Vibrio (Vibrio parahaemolyticus and Vibrio cholerae), carried out with 2 commercial antibiotics from different chemical families (1. Tetracycline, 2. Ceftriaxone) and 2 plant extracts (Eucalyptus and Guava). (DOCX) [file pone.0304126.s009.docx]

**S7 Table. Summary table of the Biofilms Inhibitory and Eradication concentration.**

| **Biofilm Inhibition Concentration Plants Extracts** | | | | | | | | | | | | | | | | | | | | | | | | | | | | | | | |
| --- | --- | --- | --- | --- | --- | --- | --- | --- | --- | --- | --- | --- | --- | --- | --- | --- | --- | --- | --- | --- | --- | --- | --- | --- | --- | --- | --- | --- | --- | --- | --- |
| **Microorganism** | | | **Control 24 H 24°C** | | | | | | | **Microorganism** | | | | **Control 72 H 30°C** | | | | | | | | | | | | | | | | | |
| ***Vibrio parahaemolyticus* (VP-87)** | | | **Abs 630**  **SD** | | | 2.90  (0.19) | | | | ***Vibrio parahemolyticus* (VP-87)** | | | | **Abs 630**  **SD** | | | | | | | | | 2.54  (0.23) | | | | | | | | |
|  |  |  | **% Inhibition Abs (%SD)** | | | -  (6.40) | | | |  |  |  |  | **% Inhibition Abs (%SD)** | | | | | | | | | -  (9.23) | | | | | | | | |
|  |  |  | **Mean CFU**  **SD** | | | 2.05E+09  (2.51E+08) | | | |  |  |  |  | **Mean CFU**  **SD** | | | | | | | | | 1.38E+09  (1.65E+08) | | | | | | | | |
|  |  |  | **% Inhibition CFU**  **%SD** | | | -  (12.27) | | | |  |  |  |  | **% Inhibition CFU**  **%SD** | | | | | | | | | -  (11.97) | | | | | | | | |
| ***Vibrio parahaemolyticus* (VP-275)** | | | **Abs 630**  **SD** | | | 2.85  (0.33) | | | | ***Vibrio parahemolyticus* (VP-275)** | | | | **Abs 630**  **SD** | | | | | | | | | 2.91  (0.23) | | | | | | | | |
|  |  |  | **% Inhibition Abs (%SD)** | | | -  (11.55) | | | |  |  |  |  | **% Inhibition Abs (%SD)** | | | | | | | | | -  (8.05) | | | | | | | | |
|  |  |  | **Mean CFU**  **SD** | | | 2.27E+09  (2.63E+08) | | | |  |  |  |  | **Mean CFU**  **SD** | | | | | | | | | 1.57E+09  (1.60E+08) | | | | | | | | |
|  |  |  | **% Inhibition CFU**  **%SD** | | | -  (11.60) | | | |  |  |  |  | **% Inhibition CFU**  **%SD** | | | | | | | | | -  (10.21) | | | | | | | | |
| ***Vibrio cholerae* (VC-112)** | | | **Abs 630**  **SD** | | | 1.14  (0.11) | | | | ***Vibrio cholerae* (VC-112)** | | | | **Abs 630**  **SD** | | | | | | | | | 2.59  (0.19) | | | | | | | | |
|  |  |  | **% Inhibition Abs (%SD)** | | | -  (9.73) | | | |  |  |  |  | **% Inhibition Abs (%SD)** | | | | | | | | | -  (7.26) | | | | | | | | |
|  |  |  | **Mean CFU**  **SD** | | | 1.59E+09  (7.83E+07) | | | |  |  |  |  | **Mean CFU**  **SD** | | | | | | | | | 1.82E+09  (2.47E+08) | | | | | | | | |
|  |  |  | **% Inhibition CFU**  **%SD** | | | -  (4.92) | | | |  |  |  |  | **% Inhibition CFU**  **%SD** | | | | | | | | | -  (13.59) | | | | | | | | |
| **Eucalyptus** | | | | | | | | | | | | | | | | | | | | | | | | | | | | | | | |
| **Microorganism** | **Concentration (μg/ml)** | | | **200** | **400** | | **800** | **1600** | **3200** | **6400** | **Concentration (μg/ml)** | | | | **200** | | | **400** | | **800** | | | | | **1600** | | | **3200** | | | **6400** |
| ***Vibrio parahaemolyticus* (VP-87)** | **24**  **H**  **24°C** | **Abs 630**  **SD** | | 0.49 | 0.37 | | 0.25 | 0.13 | 0.20 | 0.30 | **72 H**  **30°C** | | **Abs 630**  **SD** | | 0.57 | | | 0.50 | | 0.36 | | | | | 0.41 | | | 0.25 | | | 0.21 |
|  |  |  |  | 0.05 | 0.02 | | 0.03 | 0.02 | 0.02 | 0.03 |  |  |  |  | 0.05 | | | 0.05 | | 0.02 | | | | | 0.02 | | | 0.03 | | | 0.02 |
|  |  | **% Inhibition %SD** | | 83.18 | 87.32 | | 91.45 | 95.39 | 93.15 | 89.72 |  |  | **% Inhibition %SD** | | 80.51 | | | 82.82 | | 87.47 | | | | | 85.96 | | | 91.43 | | | 92.89 |
|  |  |  |  | 10.04 | 6.69 | | 10.92 | 12.71 | 9.49 | 11.48 |  |  |  |  | 8.21 | | | 10.61 | | 5.97 | | | | | 5.91 | | | 11.33 | | | 11.86 |
|  |  | **Mean CFU**  **SD** | | 1.68E+09 | 3.21E+08 | | 1.46E+08 | 1.19E+08 | 7.00E+07 | 5.00E+07 |  |  | **Mean CFU**  **SD** | | 5.02E+08 | | | 2.67E+08 | | 2.10E+08 | | | | | 1.33E+08 | | | 7.83E+07 | | | 2.50E+07 |
|  |  |  |  | 1.13E+08 | 4.57E+07 | | 2.01E+07 | 1.81E+07 | 3.33E+06 | 5.44E+06 |  |  |  |  | 1.12E+08 | | | 6.67E+06 | | 3.00E+07 | | | | | 1.19E+07 | | | 8.33E+06 | | | 5.00E+06 |
|  |  | **% Inhibition CFU**  **%SD** | | 17.92 | 84.31 | | 92.89 | 94.19 | 96.58 | 97.56 |  |  | **% Inhibition CFU**  **%SD** | | 63.56 | | | 80.63 | | 84.75 | | | | | 90.31 | | | 94.31 | | | 98.18 |
|  |  |  |  | 6.75 | 14.24 | | 13.78 | 15.24 | 4.76 | 10.89 |  |  |  |  | 22.26 | | | 2.50 | | 14.29 | | | | | 8.90 | | | 10.64 | | | 20.00 |
| ***Vibrio parahaemolyticus* (VP-275)** |  | **Abs 630**  **SD** | | 0.36 | 0.27 | | 0.21 | 0.07 | 0.23 | 0.42 |  |  | **Abs 630**  **SD** | | 0.45 | | | 0.39 | | 0.27 | | | | | 0.35 | | | 0.29 | | | 0.27 |
|  |  |  |  | 0.02 | 0.03 | | 0.01 | 0.01 | 0.02 | 0.02 |  |  |  |  | 0.04 | | | 0.03 | | 0.04 | | | | | 0.02 | | | 0.03 | | | 0.04 |
|  |  | **% Inhibition %SD** | | 87.35 | 90.38 | | 92.65 | 97.67 | 91.93 | 85.13 |  |  | **% Inhibition %SD** | | 84.12 | | | 86.16 | | 90.67 | | | | | 87.76 | | | 89.65 | | | 90.35 |
|  |  |  |  | 5.47 | 10.36 | | 6.59 | 14.19 | 7.07 | 4.88 |  |  |  |  | 9.94 | | | 7.47 | | 15.92 | | | | | 5.07 | | | 9.40 | | | 14.73 |
|  |  | **Mean CFU**  **SD** | | 1.46E+09 | 9.33E+07 | | 8.00E+07 | 1.13E+08 | 1.24E+08 | 2.00E+07 |  |  | **Mean CFU**  **SD** | | 7.93E+08 | | | 4.83E+07 | | 1.11E+07 | | | | | 1.67E+07 | | | 6.00E+07 | | | 1.35E+08 |
|  |  |  |  | 9.33E+07 | 1.00E+07 | | 6.67E+06 | 2.67E+07 | 2.08E+07 | 3.33E+06 |  |  |  |  | 1.37E+08 | | | 5.00E+06 | | 1.57E+06 | | | | | 0.00E+00 | | | 0.00E+00 | | | 1.17E+07 |
|  |  | **% Inhibition CFU**  **%SD** | | 35.54 | 95.89 | | 96.48 | 95.01 | 94.53 | 99.12 |  |  | **% Inhibition CFU**  **%SD** | | 49.36 | | | 96.91 | | 99.29 | | | | | 98.94 | | | 96.17 | | | 91.38 |
|  |  |  |  | 6.38 | 10.71 | | 8.33 | 23.53 | 16.78 | 16.67 |  |  |  |  | 17.23 | | | 10.34 | | 14.14 | | | | | 0.00 | | | 0.00 | | | 8.64 |
| ***Vibrio cholerae* (VC-112)** |  | **Abs 630**  **SD** | | 0.44 | 0.39 | | 0.28 | 0.19 | 0.16 | 0.19 |  |  | **Abs 630**  **SD** | | 0.42 | | | 0.30 | | 0.17 | | | | | 0.13 | | | 0.18 | | | 0.12 |
|  |  |  |  | 0.04 | 0.05 | | 0.03 | 0.01 | 0.01 | 0.02 |  |  |  |  | 0.03 | | | 0.03 | | 0.01 | | | | | 0.02 | | | 0.02 | | | 0.02 |
|  |  | **% Inhibition %SD** | | 61.56 | 65.45 | | 75.36 | 83.39 | 86.18 | 83.29 |  |  | **% Inhibition %SD** | | 63.41 | | | 73.93 | | 84.97 | | | | | 88.89 | | | 84.46 | | | 89.79 |
|  |  |  |  | 9.24 | 11.74 | | 11.07 | 5.52 | 8.85 | 9.45 |  |  |  |  | 6.31 | | | 9.62 | | 8.39 | | | | | 15.87 | | | 11.62 | | | 13.12 |
|  |  | **Mean CFU**  **SD** | | 6.05E+08 | 3.55E+08 | | 1.35E+08 | 3.50E+07 | 6.83E+07 | 5.00E+07 |  |  | **Mean CFU**  **SD** | | 1.02E+09 | | | 3.22E+08 | | 2.57E+08 | | | | | 2.58E+08 | | | 1.75E+08 | | | 1.73E+08 |
|  |  |  |  | 9.50E+07 | 6.17E+07 | | 1.50E+07 | 1.67E+06 | 1.17E+07 | 1.33E+07 |  |  |  |  | 1.62E+08 | | | 7.83E+07 | | 4.11E+07 | | | | | 4.50E+07 | | | 2.17E+07 | | | 3.00E+07 |
|  |  | **% Inhibition CFU**  **%SD** | | 61.99 | 77.70 | | 91.52 | 97.80 | 95.71 | 96.86 |  |  | **% Inhibition CFU**  **%SD** | | 44.13 | | | 82.29 | | 85.87 | | | | | 85.78 | | | 90.37 | | | 90.46 |
|  |  |  |  | 15.70 | 17.37 | | 11.11 | 4.76 | 17.07 | 26.67 |  |  |  |  | 15.93 | | | 24.35 | | 16.01 | | | | | 17.42 | | | 12.38 | | | 17.31 |
| **Guava** | | | | | | | | | | | | | | | | | | | | | | | | | | | | | | | |
| **Microorganism** | **Concentration (μg/ml)** | | | **1600** | **3200** | | **6400** | **12800** | **25600** | **51200** | **Concentration (μg/ml)** | | | | **1600** | | | **3200** | | **6400** | | | | | **12800** | | | **25600** | | | **51200** |
| ***Vibrio parahaemolyticus* (VP-87)** | **24**  **H**  **24°C** | **Abs 630**  **SD** | | 0.50 | 0.50 | | 0.47 | 0.32 | 0.37 | 0.24 | **72 H**  **30°C** | | **Abs 630**  **SD** | | 0.90 | | | 0.94 | | 0.79 | | | | | 0.67 | | | 0.82 | | | 0.52 |
|  |  |  |  | 0.04 | 0.05 | | 0.05 | 0.03 | 0.06 | 0.04 |  |  |  |  | 0.09 | | | 0.11 | | 0.13 | | | | | 0.09 | | | 0.13 | | | 0.07 |
|  |  | **% Inhibition %SD** | | 80.19 | 80.11 | | 81.44 | 87.49 | 85.45 | 90.64 |  |  | **% Inhibition %SD** | | 64.42 | | | 62.81 | | 68.96 | | | | | 73.42 | | | 67.83 | | | 79.42 |
|  |  |  |  | 8.49 | 9.29 | | 10.93 | 10.57 | 16.01 | 15.63 |  |  |  |  | 10.06 | | | 11.38 | | 16.41 | | | | | 13.44 | | | 15.37 | | | 14.02 |
|  |  | **Mean CFU**  **SD** | | 1.52E+09 | 5.47E+08 | | 3.57E+08 | 2.59E+08 | 1.28E+08 | 1.62E+08 |  |  | **Mean CFU**  **SD** | | 1.21E+09 | | | 8.92E+08 | | 6.91E+08 | | | | | 5.53E+08 | | | 5.02E+08 | | | 7.27E+08 |
|  |  |  |  | 1.22E+08 | 1.00E+07 | | 3.33E+06 | 3.45E+07 | 8.33E+06 | 4.50E+07 |  |  |  |  | 1.92E+08 | | | 7.50E+07 | | 1.16E+08 | | | | | 7.33E+07 | | | 2.83E+07 | | | 1.10E+08 |
|  |  | **% Inhibition**  **CFU**  **%SD** | | 25.98 | 73.29 | | 82.57 | 87.35 | 93.73 | 92.10 |  |  | **% Inhibition CFU**  **%SD** | | 12.23 | | | 35.23 | | 49.80 | | | | | 59.81 | | | 63.56 | | | 47.22 |
|  |  |  |  | 8.03 | 1.83 | | 0.93 | 13.31 | 6.49 | 27.84 |  |  |  |  | 15.86 | | | 8.41 | | 16.74 | | | | | 13.25 | | | 5.65 | | | 15.14 |
| ***Vibrio parahaemolyticus* (VP-275)** |  | **Abs 630**  **SD** | | 0.34 | 0.29 | | 0.27 | 0.18 | 0.20 | 0.19 |  |  | **Abs 630**  **SD** | | 0.99 | | | 0.47 | | 0.43 | | | | | 0.47 | | | 0.79 | | | 0.48 |
|  |  |  |  | 0.03 | 0.03 | | 0.04 | 0.02 | 0.02 | 0.03 |  |  |  |  | 0.08 | | | 0.07 | | 0.06 | | | | | 0.06 | | | 0.12 | | | 0.07 |
|  |  | **% Inhibition %SD** | | 88.44 | 90.03 | | 90.78 | 93.77 | 93.25 | 93.51 |  |  | **% Inhibition %SD** | | 66.06 | | | 83.70 | | 85.27 | | | | | 83.97 | | | 72.84 | | | 83.37 |
|  |  |  |  | 9.41 | 9.17 | | 15.25 | 10.12 | 11.80 | 16.14 |  |  |  |  | 8.44 | | | 14.24 | | 14.43 | | | | | 12.04 | | | 15.68 | | | 15.29 |
|  |  | **Mean CFU**  **SD** | | 1.53E+09 | 7.62E+08 | | 7.05E+08 | 3.65E+08 | 2.59E+08 | 1.53E+08 |  |  | **Mean CFU**  **SD** | | 1.13E+09 | | | 6.10E+08 | | 5.30E+08 | | | | | 6.13E+08 | | | 6.56E+08 | | | 5.52E+08 |
|  |  |  |  | 1.08E+08 | 1.65E+08 | | 9.83E+07 | 6.17E+07 | 3.55E+07 | 1.67E+07 |  |  |  |  | 1.60E+08 | | | 3.78E+07 | | 6.13E+07 | | | | | 6.62E+07 | | | 6.12E+07 | | | 6.38E+07 |
|  |  | **% Inhibition CFU**  **%SD** | | 32.82 | 66.45 | | 68.94 | 83.92 | 88.60 | 93.25 |  |  | **% Inhibition CFU**  **%SD** | | 28.09 | | | 61.06 | | 66.17 | | | | | 60.85 | | | 58.16 | | | 64.75 |
|  |  |  |  | 7.10 | 21.66 | | 13.95 | 16.89 | 13.72 | 10.87 |  |  |  |  | 14.20 | | | 6.20 | | 11.56 | | | | | 10.80 | | | 9.34 | | | 11.56 |
| ***Vibrio cholerae* (VC-112)** |  | **Abs 630**  **SD** | | 0.88 | 0.65 | | 0.49 | 0.50 | 0.56 | 0.69 |  |  | **Abs 630**  **SD** | | 1.15 | | | 1.02 | | 0.97 | | | | | 1.04 | | | 1.27 | | | 0.78 |
|  |  |  |  | 0.08 | 0.06 | | 0.06 | 0.08 | 0.09 | 0.10 |  |  |  |  | 0.09 | | | 0.07 | | 0.10 | | | | | 0.12 | | | 0.17 | | | 0.10 |
|  |  | **% Inhibition %SD** | | 65.82 | 74.74 | | 81.15 | 80.49 | 78.11 | 73.35 |  |  | **% Inhibition %SD** | | 55.22 | | | 60.25 | | 62.38 | | | | | 59.83 | | | 50.61 | | | 69.56 |
|  |  |  |  | 8.95 | 9.94 | | 12.51 | 15.57 | 16.29 | 14.69 |  |  |  |  | 8.15 | | | 7.32 | | 10.33 | | | | | 11.18 | | | 13.07 | | | 13.38 |
|  |  | **Mean CFU**  **SD** | | 6.05E+08 | 4.45E+08 | | 3.64E+08 | 2.33E+08 | 1.78E+08 | 1.15E+08 |  |  | **Mean CFU**  **SD** | | 1.53E+09 | | | 1.71E+09 | | 1.18E+09 | | | | | 1.55E+09 | | | 1.60E+09 | | | 1.13E+09 |
|  |  |  |  | 7.83E+07 | 2.50E+07 | | 3.06E+07 | 2.23E+07 | 8.33E+06 | 1.50E+07 |  |  |  |  | 1.34E+08 | | | 8.50E+07 | | 1.50E+08 | | | | | 7.50E+07 | | | 1.35E+08 | | | 6.00E+07 |
|  |  | **% Inhibition CFU**  **%SD** | | 61.99 | 72.04 | | 77.10 | 85.34 | 88.80 | 92.77 |  |  | **% Inhibition CFU**  **%SD** | | 15.96 | | | 5.78 | | 34.86 | | | | | 14.77 | | | 12.02 | | | 37.61 |
|  |  |  |  | 12.95 | 5.62 | | 8.39 | 9.55 | 4.67 | 13.04 |  |  |  |  | 8.76 | | | 4.97 | | 12.68 | | | | | 4.84 | | | 8.45 | | | 5.29 |
| **Biofilm Inhibition Concentration Antibiotics** | | | | | | | | | | | | | | | | | | | | | | | | | | | | | | | |
| **Microorganism** | | | **Control 24 H 24°C** | | | | | | | **Microorganism** | | | | **Control 72 H 30°C** | | | | | | | | | | | | | | | | | |
| ***Vibrio parahaemolyticus* (VP-87)** | | | **Abs 630**  **SD** | | | 2.90  (0.19) | | | | ***Vibrio parahemolyticus* (VP-87)** | | | | **Abs 630**  **SD** | | | | | | | | 2.54  (0.23) | | | | | | | | | |
|  |  |  | **% Inhibition Abs (%SD)** | | | -  (6.40) | | | |  |  |  |  | **% Inhibition Abs (%SD)** | | | | | | | | -  (9.23) | | | | | | | | | |
|  |  |  | **Mean CFU**  **SD** | | | 2.05E+09  (2.51E+08) | | | |  |  |  |  | **Mean CFU**  **SD** | | | | | | | | 1.38E+09  (1.65E+08) | | | | | | | | | |
|  |  |  | **% Inhibition CFU**  **%SD** | | | -  (12.27) | | | |  |  |  |  | **% Inhibition CFU**  **%SD** | | | | | | | | -  (11.97) | | | | | | | | | |
| ***Vibrio parahaemolyticus* (VP-275)** | | | **Abs 630**  **SD** | | | 2.85  (0.33) | | | | ***Vibrio parahemolyticus* (VP-275)** | | | | **Abs 630**  **SD** | | | | | | | | 2.91  (0.23) | | | | | | | | | |
|  |  |  | **% Inhibition Abs (%SD)** | | | -  (11.55) | | | |  |  |  |  | **% Inhibition Abs (%SD)** | | | | | | | | -  (8.05) | | | | | | | | | |
|  |  |  | **Mean CFU**  **SD** | | | 2.27E+09  (2.63E+08) | | | |  |  |  |  | **Mean CFU**  **SD** | | | | | | | | 1.57E+09  (1.60E+08) | | | | | | | | | |
|  |  |  | **% Inhibition CFU**  **%SD** | | | -  (11.60) | | | |  |  |  |  | **% Inhibition CFU**  **%SD** | | | | | | | | -  (10.21) | | | | | | | | | |
| ***Vibrio cholerae* (VC-112)** | | | **Abs 630**  **SD** | | | 1.14  (0.11) | | | | ***Vibrio cholerae* (VC-112)** | | | | **Abs 630**  **SD** | | | | | | | | 2.59  (0.19) | | | | | | | | | |
|  |  |  | **% Inhibition Abs (%SD)** | | | -  (9.73) | | | |  |  |  |  | **% Inhibition Abs (%SD)** | | | | | | | | -  (7.26) | | | | | | | | | |
|  |  |  | **Mean CFU**  **SD** | | | 1.59E+09  (7.83E+07) | | | |  |  |  |  | **Mean CFU**  **SD** | | | | | | | | 1.82E+09  (2.47E+08) | | | | | | | | | |
|  |  |  | **% Inhibition CFU**  **%SD** | | | -  (4.92) | | | |  |  |  |  | **% Inhibition CFU**  **%SD** | | | | | | | | -  (13.59) | | | | | | | | | |
| **Tetracycline** | | | | | | | | | | | | | | | | | | | | | | | | | | | | | | | |
| **Microorganism** | **Concentration (μg/ml)** | | | **2** | **5** | | **10** | **20** | **40** | **80** | **Concentration (μg/ml)** | | | | **2** | | **5** | | **10** | | | | | **20** | | | **40** | | | **80** | |
| ***Vibrio parahaemolyticus* (VP-87)** | **24**  **H**  **24°C** | **Abs 630**  **SD** | | 0.89 | 0.75 | | 0.24 | 0.14 | 0.12 | 0.06 | **72 H**  **30°C** | | **Abs 630**  **SD** | | 1.30 | | 1.33 | | 1.03 | | | | | 0.92 | | | 0.77 | | | 0.50 | |
|  |  |  |  | 0.02 | 0.05 | | 0.02 | 0.01 | 0.01 | 0.01 |  |  |  |  | 0.06 | | 0.06 | | 0.10 | | | | | 0.08 | | | 0.04 | | | 0.03 | |
|  |  | **% Inhibition %SD** | | 69.39 | 74.09 | | 91.70 | 95.06 | 95.81 | 97.92 |  |  | **% Inhibition %SD** | | 48.58 | | 47.53 | | 59.61 | | | | | 63.77 | | | 69.57 | | | 80.33 | |
|  |  |  |  | 2.61 | 6.38 | | 6.96 | 4.66 | 9.12 | 13.90 |  |  |  |  | 4.79 | | 4.17 | | 9.48 | | | | | 9.00 | | | 4.77 | | | 6.05 | |
|  |  | **Mean CFU**  **SD** | | 1.81E+09 | 1.38E+09 | | 6.67E+08 | 2.55E+08 | 1.28E+08 | 5.90E+08 |  |  | **Mean CFU**  **SD** | | 1.15E+09 | | 8.55E+08 | | 1.12E+09 | | | | | 9.22E+08 | | | 7.95E+08 | | | 1.59E+09 | |
|  |  |  |  | 1.38E+08 | 1.77E+08 | | 2.67E+07 | 4.17E+07 | 8.33E+06 | 1.50E+08 |  |  |  |  | 1.60E+08 | | 1.50E+07 | | 1.34E+08 | | | | | 4.17E+07 | | | 8.33E+06 | | | 1.83E+08 | |
|  |  | **% Inhibition CFU**  **%SD** | | 11.45 | 32.57 | | 67.43 | 87.54 | 93.73 | 71.17 |  |  | **% Inhibition CFU**  **%SD** | | 16.79 | | 37.89 | | 18.81 | | | | | 33.05 | | | 42.25 | | | -15.50 | |
|  |  |  |  | 7.61 | 12.80 | | 4.00 | 16.34 | 6.49 | 25.42 |  |  |  |  | 13.98 | | 1.75 | | 11.95 | | | | | 4.52 | | | 1.05 | | | 11.53 | |
| ***Vibrio parahaemolyticus* (VP-275)** |  | **Abs 630**  **SD** | | 1.96 | 1.77 | | 1.75 | 1.70 | 1.66 | 1.62 |  |  | **Abs 630**  **SD** | | 1.35 | | 1.31 | | 1.25 | | | | | 1.22 | | | 1.20 | | | 1.09 | |
|  |  |  |  | 0.11 | 0.14 | | 0.13 | 0.12 | 0.12 | 0.12 |  |  |  |  | 0.07 | | 0.04 | | 0.07 | | | | | 0.06 | | | 0.05 | | | 0.06 | |
|  |  | **% Inhibition %SD** | | 31.22 | 37.92 | | 38.55 | 40.29 | 41.58 | 43.08 |  |  | **% Inhibition %SD** | | 53.60 | | 55.14 | | 57.00 | | | | | 57.94 | | | 58.84 | | | 62.51 | |
|  |  |  |  | 5.65 | 8.02 | | 7.46 | 6.85 | 7.32 | 7.66 |  |  |  |  | 5.07 | | 2.81 | | 5.52 | | | | | 4.97 | | | 4.02 | | | 5.13 | |
|  |  | **Mean CFU**  **SD** | | 2.23E+09 | 1.86E+09 | | 2.15E+09 | 2.14E+09 | 1.91E+09 | 1.91E+09 |  |  | **Mean CFU**  **SD** | | 1.56E+09 | | 1.44E+09 | | 1.48E+09 | | | | | 1.02E+09 | | | 1.53E+09 | | | 1.50E+09 | |
|  |  |  |  | 1.92E+08 | 2.19E+08 | | 1.45E+08 | 1.95E+08 | 2.78E+08 | 3.45E+08 |  |  |  |  | 1.92E+08 | | 2.22E+08 | | 1.45E+08 | | | | | 2.50E+07 | | | 2.27E+08 | | | 1.62E+08 | |
|  |  | **% Inhibition CFU**  **%SD** | | 1.98 | 18.11 | | 5.36 | 5.95 | 15.91 | 16.05 |  |  | **% Inhibition CFU**  **%SD** | | 0.53 | | 8.19 | | 5.43 | | | | | 34.79 | | | 2.55 | | | 4.57 | |
|  |  |  |  | 8.61 | 11.78 | | 6.75 | 9.13 | 14.59 | 18.10 |  |  |  |  | 12.30 | | 15.41 | | 9.79 | | | | | 2.45 | | | 14.85 | | | 10.81 | |
| ***Vibrio cholerae* (VC-112)** |  | **Abs 630**  **SD** | | 0.38 | 0.15 | | 0.11 | 0.10 | 0.06 | 0.05 |  |  | **Abs 630**  **SD** | | 1.09 | | 0.90 | | 0.26 | | | | | 0.17 | | | 0.11 | | | 0.09 | |
|  |  |  |  | 0.027 | 0.011 | | 0.006 | 0.008 | 0.005 | 0.004 |  |  |  |  | 0.06 | | 0.06 | | 0.04 | | | | | 0.01 | | | 0.01 | | | 0.01 | |
|  |  | **% Inhibition %SD** | | 66.56 | 87.05 | | 90.21 | 91.14 | 94.39 | 95.71 |  |  | **% Inhibition %SD** | | 57.77 | | 65.24 | | 90.00 | | | | | 93.47 | | | 95.83 | | | 96.55 | |
|  |  |  |  | 6.99 | 7.51 | | 5.64 | 8.20 | 7.44 | 7.77 |  |  |  |  | 5.84 | | 6.75 | | 14.16 | | | | | 4.08 | | | 6.11 | | | 6.54 | |
|  |  | **Mean CFU**  **SD** | | 7.69E+08 | 3.97E+08 | | 3.85E+08 | 2.48E+08 | 1.23E+08 | 1.24E+09 |  |  | **Mean CFU**  **SD** | | 1.22E+09 | | 1.68E+09 | | 1.43E+09 | | | | | 7.30E+08 | | | 6.60E+08 | | | 3.52E+08 | |
|  |  |  |  | 1.08E+08 | 2.67E+07 | | 1.17E+07 | 8.33E+06 | 6.67E+06 | 1.30E+08 |  |  |  |  | 6.00E+07 | | 3.08E+08 | | 3.92E+08 | | | | | 1.40E+08 | | | 1.57E+08 | | | 3.83E+07 | |
|  |  | **% Inhibition CFU**  **%SD** | | 51.69 | 75.08 | | 75.81 | 84.40 | 92.25 | 21.88 |  |  | **% Inhibition CFU**  **%SD** | | 32.66 | | 7.61 | | 21.19 | | | | | 59.82 | | | 63.67 | | | 80.64 | |
|  |  |  |  | 14.09 | 6.72 | | 3.03 | 3.36 | 5.41 | 10.46 |  |  |  |  | 4.90 | | 18.37 | | 27.36 | | | | | 19.18 | | | 23.74 | | | 10.90 | |
| **Ceftriaxone** | | | | | | | | | | | | | | | | | | | | | | | | | | | | | | | |
| **Microorganism** | **Concentration (μg/ml)** | | | **2** | **5** | | **10** | **20** | **40** | **80** | **Concentration (μg/ml)** | | | | **2** | | **5** | | **10** | | | | | **20** | | | **40** | | | **80** | |
| ***Vibrio parahaemolyticus* (VP-87)** | **24**  **H**  **24°C** | **Abs 630**  **SD** | | 0.27 | 0.26 | | 0.16 | 0.11 | 0.08 | 0.07 | **72 H**  **30°C** | | **Abs 630**  **SD** | | 0.30 | | 0.29 | | 0.27 | | | | | 0.25 | | | 0.21 | | | 0.19 | |
|  |  |  |  | 0.009 | 0.007 | | 0.014 | 0.002 | 0.005 | 0.006 |  |  |  |  | 0.007 | | 0.006 | | 0.004 | | | | | 0.007 | | | 0.005 | | | 0.005 | |
|  |  | **% Inhibition %SD** | | 90.54 | 90.96 | | 94.62 | 96.35 | 97.40 | 97.52 |  |  | **% Inhibition %SD** | | 88.36 | | 88.76 | | 89.46 | | | | | 90.23 | | | 91.87 | | | 92.50 | |
|  |  |  |  | 3.21 | 2.65 | | 8.83 | 1.91 | 6.99 | 7.73 |  |  |  |  | 2.26 | | 2.03 | | 1.56 | | | | | 2.82 | | | 2.22 | | | 2.56 | |
|  |  | **Mean CFU**  **SD** | | 4.98E+08 | 5.22E+08 | | 8.40E+08 | 5.40E+08 | 7.82E+08 | 7.78E+08 |  |  | **Mean CFU**  **SD** | | 4.12E+08 | | 4.78E+08 | | 4.23E+08 | | | | | 2.40E+08 | | | 6.83E+08 | | | 3.12E+08 | |
|  |  |  |  | 2.83E+07 | 1.50E+07 | | 2.00E+07 | 2.67E+07 | 4.50E+07 | 3.83E+07 |  |  |  |  | 1.81E+07 | | 3.85E+07 | | 3.33E+06 | | | | | 2.67E+07 | | | 1.22E+08 | | | 6.17E+07 | |
|  |  | **% Inhibition**  **CFU**  **%SD** | | 75.65 | 74.51 | | 58.96 | 73.62 | 61.81 | 61.97 |  |  | **% Inhibition CFU**  **%SD** | | 70.06 | | 65.29 | | 69.25 | | | | | 82.57 | | | 50.36 | | | 77.36 | |
|  |  |  |  | 5.69 | 2.88 | | 2.38 | 4.94 | 5.76 | 4.93 |  |  |  |  | 4.40 | | 8.06 | | 0.79 | | | | | 11.11 | | | 17.91 | | | 19.79 | |
| ***Vibrio parahaemolyticus* (VP-275)** |  | **Abs 630**  **SD** | | 0.31 | 0.29 | | 0.19 | 0.08 | 0.08 | 0.07 |  |  | **Abs 630**  **SD** | | 0.23 | | 0.22 | | 0.21 | | | | | 0.21 | | | 0.19 | | | 0.11 | |
|  |  |  |  | 0.03 | 0.01 | | 0.01 | 0.01 | 0.01 | 0.01 |  |  |  |  | 0.007 | | 0.008 | | 0.010 | | | | | 0.003 | | | 0.004 | | | 0.009 | |
|  |  | **% Inhibition %SD** | | 88.99 | 89.71 | | 93.47 | 97.22 | 97.20 | 97.65 |  |  | **% Inhibition %SD** | | 91.94 | | 92.30 | | 92.62 | | | | | 92.94 | | | 93.51 | | | 96.34 | |
|  |  |  |  | 9.45 | 3.84 | | 6.87 | 7.97 | 9.62 | 9.38 |  |  |  |  | 2.95 | | 3.56 | | 4.67 | | | | | 1.24 | | | 2.21 | | | 8.06 | |
|  |  | **Mean CFU**  **SD** | | v | 5.58E+08 | | 6.86E+08 | 6.02E+08 | 6.09E+08 | 5.30E+08 |  |  | **Mean CFU**  **SD** | | 3.33E+08 | | 5.97E+08 | | 3.93E+08 | | | | | 3.72E+08 | | | 3.64E+08 | | | 3.83E+08 | |
|  |  |  |  | 2.50E+07 | 8.33E+06 | | 8.33E+07 | 5.00E+06 | 4.94E+07 | 3.00E+07 |  |  |  |  | 6.67E+07 | | 1.30E+08 | | 0.00E+00 | | | | | 5.28E+07 | | | 2.27E+07 | | | 3.09E+07 | |
|  |  | **% Inhibition CFU**  **%SD** | | 76.87 | 75.40 | | 69.80 | 73.49 | 73.18 | 76.65 |  |  | **% Inhibition CFU**  **%SD** | | 78.72 | | 61.91 | | 74.89 | | | | | 76.24 | | | 76.74 | | | 75.53 | |
|  |  |  |  | 4.76 | 1.49 | | 12.15 | 0.83 | 8.11 | 5.66 |  |  |  |  | 20.00 | | 21.79 | | 0.00 | | | | | 14.18 | | | 6.22 | | | 8.06 | |
| ***Vibrio cholerae* (VC-112)** |  | **Abs 630**  **SD** | | 0.20 | 0.19 | | 0.17 | 0.08 | 0.07 | 0.06 |  |  | **Abs 630**  **SD** | | 0.17 | | 0.17 | | 0.17 | | | | | 0.16 | | | 0.16 | | | 0.15 | |
|  |  |  |  | 0.008 | 0.008 | | 0.013 | 0.007 | 0.004 | 0.003 |  |  |  |  | 0.005 | | 0.005 | | 0.003 | | | | | 0.003 | | | 0.002 | | | 0.005 | |
|  |  | **% Inhibition %SD** | | 82.18 | 83.19 | | 85.50 | 92.75 | 93.60 | 94.70 |  |  | **% Inhibition %SD** | | 93.27 | | 93.23 | | 93.37 | | | | | 93.61 | | | 93.79 | | | 94.21 | |
|  |  |  |  | 4.07 | 3.95 | | 7.68 | 8.81 | 4.86 | 5.28 |  |  |  |  | 2.90 | | 2.99 | | 1.67 | | | | | 1.86 | | | 1.29 | | | 3.04 | |
|  |  | **Mean CFU**  **SD** | | 4.40E+08 | 3.30E+08 | | 4.20E+08 | 4.72E+08 | 7.93E+08 | 3.82E+08 |  |  | **Mean CFU**  **SD** | | 5.57E+08 | | 7.05E+08 | | 3.85E+08 | | | | | 5.67E+08 | | | 6.43E+08 | | | 7.15E+08 | |
|  |  |  |  | 2.33E+07 | 4.00E+07 | | 5.04E+07 | 6.50E+07 | 4.33E+07 | 8.33E+06 |  |  |  |  | 6.00E+07 | | 5.17E+07 | | 1.17E+07 | | | | | 2.23E+07 | | | 2.67E+07 | | | 8.33E+06 | |
|  |  | **% Inhibition CFU**  **%SD** | | 72.36 | 79.27 | | 73.61 | 70.37 | 50.16 | 76.02 |  |  | **% Inhibition CFU**  **%SD** | | 69.36 | | 61.19 | | 78.81 | | | | | 68.81 | | | 64.59 | | | 60.64 | |
|  |  |  |  | 5.30 | 12.12 | | 12.00 | 13.78 | 5.46 | 2.18 |  |  |  |  | 10.78 | | 7.33 | | 3.03 | | | | | 3.93 | | | 4.15 | | | 1.17 | |
| **Biofilm Eradication concentration Plants Extracts** | | | | | | | | | | | | | | | | | | | | | | | | | | | | | | | |
| **Microorganism** | | | **Control 24 H 24°C** | | | | | | | **Microorganism** | | | | **Control 72 H 30°C** | | | | | | | | | | | | | | | | | |
| ***Vibrio parahaemolyticus* (VP-87)** | | | **Abs 630**  **SD** | | | 2.84  (0.14) | | | | ***Vibrio parahemolyticus* (VP-87)** | | | | **Abs 630**  **SD** | | | | | | | 2.46  (0.19) | | | | | | | | | | |
|  |  |  | **% Eradication Abs (%SD)** | | | -  (4.98) | | | |  |  |  |  | **% Eradication Abs (%SD)** | | | | | | | -  (7.64) | | | | | | | | | | |
|  |  |  | **Mean CFU**  **SD** | | | 2.05E+09  (2.51E+08) | | | |  |  |  |  | **Mean CFU**  **SD** | | | | | | | 1.64E+09  (1.15E+08) | | | | | | | | | | |
|  |  |  | **% Eradication CFU**  **%SD** | | | -  (12.27) | | | |  |  |  |  | **% Eradication CFU**  **%SD** | | | | | | | -  (6.98) | | | | | | | | | | |
| ***Vibrio parahaemolyticus* (VP-275)** | | | **Abs 630**  **SD** | | | 2.85  (0.15) | | | | ***Vibrio parahemolyticus* (VP-275)** | | | | **Abs 630**  **SD** | | | | | | | 2.86  (0.13) | | | | | | | | | | |
|  |  |  | **% Eradication Abs (%SD)** | | | -  (5.15) | | | |  |  |  |  | **% Eradication Abs (%SD)** | | | | | | | -  (4.66) | | | | | | | | | | |
|  |  |  | **Mean CFU**  **SD** | | | 1.87E+09  (1.31E+08) | | | |  |  |  |  | **Mean CFU**  **SD** | | | | | | | 1.61E+09  (1.71E+08) | | | | | | | | | | |
|  |  |  | **% Eradication CFU**  **%SD** | | | -  (7.04) | | | |  |  |  |  | **% Eradication CFU**  **%SD** | | | | | | | -  (10.61) | | | | | | | | | | |
| ***Vibrio cholerae* (VC-112)** | | | **Abs 630**  **SD** | | | 1.61  (0.10) | | | | ***Vibrio cholerae* (VC-112)** | | | | **Abs 630**  **SD** | | | | | | | 2.52  (0.15) | | | | | | | | | | |
|  |  |  | **% Eradication Abs (%SD)** | | | -  (8.58) | | | |  |  |  |  | **% Eradication Abs (%SD)** | | | | | | | -  (5.98) | | | | | | | | | | |
|  |  |  | **Mean CFU**  **SD** | | | 1.18E+09  (1.90E+08) | | | |  |  |  |  | **Mean CFU**  **SD** | | | | | | | 2.19E+09  (2.93E+08) | | | | | | | | | | |
|  |  |  | **% Eradication CFU**  **%SD** | | | -  (16.15) | | | |  |  |  |  | **% Eradication CFU**  **%SD** | | | | | | | -  (13.36) | | | | | | | | | | |
| **Eucalyptus** | | | | | | | | | | | | | | | | | | | | | | | | | | | | | | | |
| **Microorganism** | **Concentration (μg/ml)** | | | **200** | **400** | | **800** | **1600** | **3200** | **6400** | **Concentration (μg/ml)** | | | | **200** | | **400** | | **800** | | | | | **1600** | | **3200** | | | **6400** | | |
| ***Vibrio parahaemolyticus* (VP-87)** | **24**  **H**  **24°C** | **Abs 630**  **SD** | | 1.59 | 1.12 | | 0.98 | 0.78 | 0.94 | 0.92 | **72 H**  **30°C** | **Abs 630**  **SD** | | | 1.03 | | 0.86 | | 0.71 | | | | | 0.53 | | 0.51 | | | 0.43 | | |
|  |  |  |  | 0.03 | 0.07 | | 0.04 | 0.06 | 0.10 | 0.09 |  |  |  |  | 0.03 | | 0.05 | | 0.03 | | | | | 0.04 | | 0.04 | | | 0.01 | | |
|  |  | **% Eradication %SD** | | 43.90 | 60.60 | | 65.47 | 72.68 | 67.04 | 67.72 |  | **% Eradication %SD** | | | 57.93 | | 64.85 | | 70.89 | | | | | 78.29 | | 79.35 | | | 82.30 | | |
|  |  |  |  | 2.00 | 6.19 | | 4.38 | 8.35 | 10.68 | 9.55 |  |  |  |  | 2.50 | | 5.39 | | 4.31 | | | | | 8.35 | | 7.99 | | | 2.55 | | |
|  |  | **Mean CFU**  **SD** | | 1.00E+09 | 3.32E+08 | | 1.90E+08 | 1.41E+08 | 7.11E+07 | 3.78E+07 |  | **Mean CFU**  **SD** | | | 8.17E+08 | | 1.67E+08 | | 5.58E+08 | | | | | 1.25E+08 | | 8.78E+07 | | | 5.17E+07 | | |
|  |  |  |  | 1.65E+08 | 2.88E+07 | | 3.86E+07 | 1.34E+07 | 4.16E+06 | 4.16E+06 |  |  |  |  | 2.03E+08 | | 6.67E+06 | | 4.50E+07 | | | | | 1.67E+06 | | 1.23E+07 | | | 1.67E+06 | | |
|  |  | **% Eradication CFU**  **%SD** | | 51.14 | 83.77 | | 90.72 | 93.11 | 96.53 | 98.15 |  | **% Eradication CFU**  **%SD** | | | 50.30 | | 89.86 | | 66.02 | | | | | 92.39 | | 94.66 | | | 96.86 | | |
|  |  |  |  | 16.52 | 8.68 | | 20.31 | 9.51 | 5.85 | 11.00 |  |  |  |  | 24.90 | | 4.00 | | 8.06 | | | | | 1.33 | | 13.98 | | | 3.23 | | |
| ***Vibrio parahaemolyticus* (VP-275)** |  | **Abs 630**  **SD** | | 0.82 | 0.71 | | 0.68 | 0.60 | 0.58 | 0.57 |  | **Abs 630**  **SD** | | | 1.01 | | 0.90 | | 0.79 | | | | | 0.75 | | 0.71 | | | 0.60 | | |
|  |  |  |  | 0.04 | 0.05 | | 0.04 | 0.04 | 0.03 | 0.06 |  |  |  |  | 0.00 | | 0.03 | | 0.04 | | | | | 0.04 | | 0.06 | | | 0.10 | | |
|  |  | **% Eradication %SD** | | 71.10 | 75.01 | | 76.14 | 78.79 | 79.72 | 80.12 |  | **% Eradication %SD** | | | 64.69 | | 68.50 | | 72.47 | | | | | 73.76 | | 75.08 | | | 79.14 | | |
|  |  |  |  | 4.77 | 6.75 | | 6.44 | 6.39 | 4.59 | 9.91 |  |  |  |  | 0.37 | | 3.47 | | 5.39 | | | | | 5.17 | | 7.92 | | | 16.86 | | |
|  |  | **Mean CFU**  **SD** | | 7.97E+08 | 4.80E+08 | | 8.00E+07 | 8.83E+07 | 4.67E+07 | 5.00E+07 |  | **Mean CFU**  **SD** | | | 5.27E+08 | | 1.83E+07 | | 1.11E+07 | | | | | 1.67E+07 | | 6.17E+07 | | | 1.09E+08 | | |
|  |  |  |  | 9.33E+07 | 1.00E+07 | | 6.67E+06 | 5.00E+06 | 1.00E+07 | 3.33E+06 |  |  |  |  | 3.00E+07 | | 1.67E+06 | | 1.57E+06 | | | | | 0.00E+00 | | 1.67E+06 | | | 1.23E+07 | | |
|  |  | **% Eradication CFU**  **%SD** | | 57.30 | 74.27 | | 95.71 | 95.27 | 97.50 | 97.32 |  | **% Eradication CFU**  **%SD** | | | 67.36 | | 98.86 | | 99.31 | | | | | 98.97 | | 96.18 | | | 93.25 | | |
|  |  |  |  | 11.72 | 2.08 | | 8.33 | 5.66 | 21.43 | 6.67 |  |  |  |  | 5.70 | | 9.09 | | 14.14 | | | | | 0.00 | | 2.70 | | | 11.27 | | |
| ***Vibrio cholerae* (VC-112)** |  | **Abs 630**  **SD** | | 0.72 | 0.69 | | 0.62 | 0.60 | 0.67 | 0.79 |  | **Abs 630**  **SD** | | | 2.17 | | 1.97 | | 1.88 | | | | | 1.56 | | 1.54 | | | 1.24 | | |
|  |  |  |  | 0.07 | 0.06 | | 0.06 | 0.07 | 0.07 | 0.07 |  |  |  |  | 0.04 | | 0.08 | | 0.07 | | | | | 0.07 | | 0.06 | | | 0.12 | | |
|  |  | **% Eradication %SD** | | 54.99 | 57.36 | | 61.68 | 62.49 | 58.59 | 51.19 |  | **% Eradication %SD** | | | 13.66 | | 21.78 | | 25.32 | | | | | 38.07 | | 38.95 | | | 50.69 | | |
|  |  |  |  | 9.81 | 8.79 | | 9.35 | 10.81 | 11.24 | 8.65 |  |  |  |  | 1.85 | | 4.29 | | 3.55 | | | | | 4.27 | | 4.04 | | | 9.30 | | |
|  |  | **Mean CFU**  **SD** | | 7.72E+08 | 3.38E+08 | | 1.02E+08 | 3.00E+07 | 4.83E+07 | 6.17E+07 |  | **Mean CFU**  **SD** | | | 7.53E+08 | | 2.54E+08 | | 2.12E+08 | | | | | 2.25E+08 | | 1.32E+08 | | | 8.44E+07 | | |
|  |  |  |  | 7.17E+07 | 3.68E+07 | | 1.83E+07 | 3.33E+06 | 1.67E+06 | 1.67E+06 |  |  |  |  | 1.34E+08 | | 3.36E+07 | | 1.75E+07 | | | | | 1.17E+07 | | 1.64E+07 | | | 1.34E+07 | | |
|  |  | **% Eradication CFU**  **%SD** | | 34.54 | 71.35 | | 91.38 | 97.46 | 95.90 | 94.77 |  | **% Eradication CFU**  **%SD** | | | 65.67 | | 88.41 | | 90.33 | | | | | 89.75 | | 93.97 | | | 96.15 | | |
|  |  |  |  | 9.29 | 10.88 | | 18.03 | 11.11 | 3.45 | 2.70 |  |  |  |  | 17.84 | | 13.20 | | 8.25 | | | | | 5.19 | | 12.41 | | | 15.90 | | |
| **Guava** | | | | | | | | | | | | | | | | | | | | | | | | | | | | | | | |
| **Microorganism** | **Concentration (μg/ml)** | | | **1600** | **3200** | | **6400** | **12800** | **25600** | **51200** | **Concentration (μg/ml)** | | | | **1600** | | **3200** | | **6400** | | | | | **12800** | | **25600** | | | **51200** | | |
| ***Vibrio parahaemolyticus* (VP-87)** | **24**  **H**  **24°C** | **Abs 630**  **SD** | | 1.35 | 1.19 | | 0.99 | 0.87 | 0.82 | 0.84 | **72 H**  **30°C** | **Abs 630**  **SD** | | | 0.96 | | 0.92 | | 0.72 | | | | | 0.68 | | 0.53 | | | 0.55 | | |
|  |  |  |  | 0.06 | 0.11 | | 0.05 | 0.08 | 0.04 | 0.11 |  |  |  |  | 0.07 | | 0.07 | | 0.05 | | | | | 0.04 | | 0.04 | | | 0.07 | | |
|  |  | **% Eradication %SD** | | 52.64 | 58.25 | | 65.25 | 69.50 | 71.28 | 70.35 |  | **% Eradication %SD** | | | 61.04 | | 62.74 | | 70.87 | | | | | 72.46 | | 78.26 | | | 77.77 | | |
|  |  |  |  | 4.18 | 9.48 | | 4.73 | 8.78 | 4.94 | 12.71 |  |  |  |  | 6.95 | | 7.97 | | 6.68 | | | | | 6.07 | | 8.03 | | | 12.61 | | |
|  |  | **Mean CFU**  **SD** | | 1.08E+09 | 5.33E+08 | | 6.17E+08 | 1.73E+08 | 1.36E+08 | 1.92E+08 |  | **Mean CFU**  **SD** | | | 8.75E+08 | | 5.58E+08 | | 2.87E+08 | | | | | 5.53E+08 | | 1.68E+08 | | | 2.62E+08 | | |
|  |  |  |  | 1.60E+08 | 2.05E+07 | | 2.33E+07 | 2.67E+07 | 1.97E+07 | 1.81E+07 |  |  |  |  | 1.92E+08 | | 7.50E+07 | | 7.00E+07 | | | | | 7.33E+07 | | 2.83E+07 | | | 2.17E+07 | | |
|  |  | **% Eradication CFU**  **%SD** | | 47.29 | 73.94 | | 69.87 | 91.53 | 93.38 | 90.61 |  | **% Eradication CFU**  **%SD** | | | 46.75 | | 66.02 | | 82.56 | | | | | 66.33 | | 89.76 | | | 84.08 | | |
|  |  |  |  | 14.85 | 3.85 | | 3.78 | 15.38 | 14.52 | 9.43 |  |  |  |  | 21.90 | | 13.43 | | 24.42 | | | | | 13.25 | | 16.83 | | | 8.28 | | |
| ***Vibrio parahaemolyticus* (VP-275)** |  | **Abs 630**  **SD** | | 0.90 | 0.74 | | 0.70 | 0.71 | 0.70 | 0.62 |  | **Abs 630**  **SD** | | | 1.00 | | 0.91 | | 0.87 | | | | | 0.70 | | 0.67 | | | 0.57 | | |
|  |  |  |  | 0.04 | 0.07 | | 0.02 | 0.06 | 0.06 | 0.06 |  |  |  |  | 0.02 | | 0.04 | | 0.06 | | | | | 0.05 | | 0.06 | | | 0.03 | | |
|  |  | **% Eradication %SD** | | 68.42 | 74.00 | | 75.53 | 75.18 | 75.39 | 78.40 |  | **% Eradication %SD** | | | 65.09 | | 68.02 | | 69.62 | | | | | 75.42 | | 76.65 | | | 80.11 | | |
|  |  |  |  | 4.65 | 9.21 | | 2.17 | 9.00 | 7.95 | 9.28 |  |  |  |  | 2.49 | | 4.77 | | 6.54 | | | | | 7.70 | | 8.65 | | | 6.09 | | |
|  |  | **Mean CFU**  **SD** | | 1.23E+09 | 8.72E+08 | | 8.34E+08 | 2.94E+08 | 1.92E+08 | 8.22E+07 |  | **Mean CFU**  **SD** | | | 1.14E+09 | | 5.87E+08 | | 5.08E+08 | | | | | 5.87E+08 | | 6.10E+08 | | | 4.98E+08 | | |
|  |  |  |  | 1.83E+08 | 1.17E+07 | | 2.91E+07 | 2.53E+07 | 2.44E+07 | 1.50E+07 |  |  |  |  | 1.45E+08 | | 4.67E+07 | | 3.65E+07 | | | | | 6.67E+07 | | 4.67E+07 | | | 8.33E+06 | | |
|  |  | **% Eradication CFU**  **%SD** | | 33.89 | 53.28 | | 55.27 | 84.22 | 89.70 | 95.59 |  | **% Eradication CFU**  **%SD** | | | 29.24 | | 63.64 | | 68.53 | | | | | 63.64 | | 62.19 | | | 69.11 | | |
|  |  |  |  | 14.84 | 1.34 | | 3.49 | 8.59 | 12.69 | 18.23 |  |  |  |  | 12.70 | | 7.95 | | 7.20 | | | | | 11.36 | | 7.65 | | | 1.67 | | |
| ***Vibrio cholerae* (VC-112)** |  | **Abs 630**  **SD** | | 1.30 | 1.23 | | 1.21 | 1.13 | 1.10 | 1.15 |  | **Abs 630**  **SD** | | | 2.07 | | 1.93 | | 1.64 | | | | | 1.54 | | 1.18 | | | 1.09 | | |
|  |  |  |  | 0.03 | 0.16 | | 0.10 | 0.08 | 0.19 | 0.02 |  |  |  |  | 0.09 | | 0.08 | | 0.08 | | | | | 0.03 | | 0.03 | | | 0.04 | | |
|  |  | **% Eradication %SD** | | 19.39 | 23.87 | | 24.97 | 29.97 | 31.80 | 28.52 |  | **% Eradication %SD** | | | 17.71 | | 23.21 | | 34.75 | | | | | 38.98 | | 53.00 | | | 56.76 | | |
|  |  |  |  | 2.15 | 13.41 | | 8.65 | 7.31 | 17.10 | 2.08 |  |  |  |  | 4.57 | | 4.29 | | 4.60 | | | | | 2.19 | | 2.93 | | | 3.67 | | |
|  |  | **Mean CFU**  **SD** | | 5.80E+08 | 3.71E+08 | | 2.98E+08 | 1.89E+08 | 1.24E+08 | 6.22E+07 |  | **Mean CFU**  **SD** | | | 1.19E+09 | | 1.38E+09 | | 1.18E+09 | | | | | 1.22E+09 | | 1.27E+09 | | | 8.00E+08 | | |
|  |  |  |  | 6.50E+07 | 3.65E+07 | | 2.04E+07 | 1.23E+07 | 2.11E+07 | 4.16E+06 |  |  |  |  | 1.34E+08 | | 8.50E+07 | | 1.77E+08 | | | | | 7.50E+07 | | 1.35E+08 | | | 6.00E+07 | | |
|  |  | **% Eradication CFU**  **%SD** | | 50.80 | 68.52 | | 74.74 | 83.98 | 89.44 | 94.72 |  | **% Eradication CFU**  **%SD** | | | 45.62 | | 37.19 | | 46.38 | | | | | 44.63 | | 42.35 | | | 63.54 | | |
|  |  |  |  | 11.21 | 9.85 | | 6.86 | 6.50 | 16.99 | 6.68 |  |  |  |  | 11.21 | | 6.17 | | 15.01 | | | | | 6.17 | | 10.67 | | | 7.50 | | |
| **Biofilm Eradication Concentration Antibiotics** | | | | | | | | | | | | | | | | | | | | | | | | | | | | | | | |
| **Microorganism** | | | **Control 24 H 24°C** | | | | | | | **Microorganism** | | | | **Control 72 H 30°C** | | | | | | | | | | | | | | | | | |
| ***Vibrio parahaemolyticus* (VP-87)** | | | **Abs 630**  **SD** | | | 2.84  (0.14) | | | | **Vibrio parahemolyticus (VP-87)** | | | | **Abs 630**  **SD** | | | | | | | 2.46  (0.19) | | | | | | | | | | |
|  |  |  | **% Eradication Abs (%SD)** | | | -  (4.98) | | | |  |  |  |  | **% Eradication Abs (%SD)** | | | | | | | -  (7.64) | | | | | | | | | | |
|  |  |  | **Mean CFU**  **SD** | | | 2.05E+09  (2.51E+08) | | | |  |  |  |  | **Mean CFU**  **SD** | | | | | | | 1.64E+09  (1.15E+08) | | | | | | | | | | |
|  |  |  | **% Eradication CFU**  **%SD** | | | -  (12.27) | | | |  |  |  |  | **% Eradication CFU**  **%SD** | | | | | | | -  (6.98) | | | | | | | | | | |
| ***Vibrio parahaemolyticus* (VP-275)** | | | **Abs 630**  **SD** | | | 2.85  (0.15) | | | | **Vibrio parahemolyticus (VP-275)** | | | | **Abs 630**  **SD** | | | | | | | 2.86  (0.13) | | | | | | | | | | |
|  |  |  | **% Eradication Abs (%SD)** | | | -  (5.15) | | | |  |  |  |  | **% Eradication Abs (%SD)** | | | | | | | -  (4.66) | | | | | | | | | | |
|  |  |  | **Mean CFU**  **SD** | | | 1.87E+09  (1.31E+08) | | | |  |  |  |  | **Mean CFU**  **SD** | | | | | | | 1.61E+09  (1.71E+08) | | | | | | | | | | |
|  |  |  | **% Eradication CFU**  **%SD** | | | -  (7.04) | | | |  |  |  |  | **% Eradication CFU**  **%SD** | | | | | | | -  (10.61) | | | | | | | | | | |
| ***Vibrio cholerae* (VC-112)** | | | **Abs 630**  **SD** | | | 1.61  (0.10) | | | | **Vibrio cholerae (VC-112)** | | | | **Abs 630**  **SD** | | | | | | | 2.52  (0.15) | | | | | | | | | | |
|  |  |  | **% Eradication Abs (%SD)** | | | -  (8.58) | | | |  |  |  |  | **% Eradication Abs (%SD)** | | | | | | | -  (5.98) | | | | | | | | | | |
|  |  |  | **Mean CFU**  **SD** | | | 1.18E+09  (1.90E+08) | | | |  |  |  |  | **Mean CFU**  **SD** | | | | | | | 2.19E+09  (2.93E+08) | | | | | | | | | | |
|  |  |  | **% Eradication CFU**  **%SD** | | | -  (16.15) | | | |  |  |  |  | **% Eradication CFU**  **%SD** | | | | | | | -  (13.36) | | | | | | | | | | |
| **Tetracycline** | | | | | | | | | | | | | | | | | | | | | | | | | | | | | | | |
| **Microorganism** | **Concentration (μg/ml)** | | | **2** | **5** | | **10** | **20** | **40** | **80** | **Concentration (μg/ml)** | | | | **2** | | **5** | | **10** | | | | | **20** | | **40** | | | **80** | | |
| ***Vibrio parahaemolyticus* (VP-87)** | **24**  **H**  **24°C** | **Abs 630**  **SD** | | 1.73 | 1.74 | | 1.56 | 1.59 | 1.63 | 1.45 | **72 H**  **30°C** | **Abs 630**  **SD** | | | 1.41 | | 1.24 | | 0.60 | | | | | 0.27 | | 0.15 | | | 0.13 | | |
|  |  |  |  | 0.06 | 0.09 | | 0.10 | 0.15 | 0.16 | 0.09 |  |  |  |  | 0.07 | | 0.16 | | 0.05 | | | | | 0.03 | | 0.02 | | | 0.01 | | |
|  |  | **% Eradication %SD** | | 39.10 | 38.82 | | 44.96 | 44.17 | 42.47 | 49.04 |  | **% Eradication %SD** | | | 42.65 | | 49.51 | | 75.62 | | | | | 89.19 | | 94.06 | | | 94.88 | | |
|  |  |  |  | 3.55 | 5.23 | | 6.19 | 9.22 | 10.01 | 6.41 |  |  |  |  | 5.28 | | 12.52 | | 9.00 | | | | | 12.85 | | 11.57 | | | 5.86 | | |
|  |  | **Mean CFU**  **SD** | | 1.81E+09 | 1.38E+09 | | 6.67E+08 | 2.55E+08 | 1.28E+08 | 9.45E+08 |  | **Mean CFU**  **SD** | | | 1.15E+09 | | 8.55E+08 | | 1.12E+09 | | | | | 1.04E+09 | | 7.95E+08 | | | 1.59E+09 | | |
|  |  |  |  | 1.38E+08 | 1.77E+08 | | 2.67E+07 | 4.17E+07 | 8.33E+06 | 2.05E+08 |  |  |  |  | 1.60E+08 | | 1.50E+07 | | 1.34E+08 | | | | | 1.66E+08 | | 8.33E+06 | | | 1.83E+08 | | |
|  |  | **% Eradication CFU**  **%SD** | | 11.45 | 32.57 | | 67.43 | 87.54 | 93.73 | 53.83 |  | **% Eradication CFU**  **%SD** | | | 30.29 | | 47.97 | | 31.98 | | | | | 36.92 | | 51.62 | | | 3.25 | | |
|  |  |  |  | 7.61 | 12.80 | | 4.00 | 16.34 | 6.49 | 21.69 |  |  |  |  | 13.98 | | 1.75 | | 11.95 | | | | | 16.03 | | 1.05 | | | 11.53 | | |
| ***Vibrio parahaemolyticus* (VP-275)** |  | **Abs 630**  **SD** | | 1.96 | 1.59 | | 1.41 | 1.49 | 1.40 | 1.34 |  | **Abs 630**  **SD** | | | 1.85 | | 1.86 | | 1.84 | | | | | 1.76 | | 1.81 | | | 1.66 | | |
|  |  |  |  | 0.02 | 0.17 | | 0.11 | 0.09 | 0.09 | 0.08 |  |  |  |  | 0.05 | | 0.04 | | 0.08 | | | | | 0.10 | | 0.07 | | | 0.08 | | |
|  |  | **% Eradication %SD** | | 31.23 | 44.16 | | 50.43 | 47.72 | 50.76 | 53.19 |  | **% Eradication %SD** | | | 35.37 | | 34.97 | | 35.73 | | | | | 38.20 | | 36.51 | | | 41.83 | | |
|  |  |  |  | 1.16 | 10.90 | | 7.53 | 6.04 | 6.20 | 5.75 |  |  |  |  | 2.78 | | 2.34 | | 4.33 | | | | | 5.85 | | 3.81 | | | 5.09 | | |
|  |  | **Mean CFU**  **SD** | | 1.56E+09 | 1.53E+09 | | 1.82E+09 | 1.80E+09 | 1.58E+09 | 1.16E+09 |  | **Mean CFU**  **SD** | | | 1.56E+09 | | 1.44E+09 | | 1.48E+09 | | | | | 1.33E+09 | | 1.53E+09 | | | 1.50E+09 | | |
|  |  |  |  | 1.42E+08 | 2.19E+08 | | 1.45E+08 | 1.95E+08 | 2.78E+08 | 1.62E+08 |  |  |  |  | 1.92E+08 | | 2.22E+08 | | 1.45E+08 | | | | | 2.80E+08 | | 2.27E+08 | | | 1.62E+08 | | |
|  |  | **% Eradication CFU**  **%SD** | | 16.47 | 18.23 | | 2.71 | 3.42 | 15.54 | 37.73 |  | **% Eradication CFU**  **%SD** | | | 3.41 | | 10.85 | | 8.16 | | | | | 17.77 | | 5.37 | | | 7.33 | | |
|  |  |  |  | 9.09 | 14.36 | | 7.99 | 10.82 | 17.67 | 13.92 |  |  |  |  | 12.30 | | 15.41 | | 9.79 | | | | | 21.11 | | 14.85 | | | 10.81 | | |
| ***Vibrio cholerae* (VC-112)** |  | **Abs 630**  **SD** | | 0.82 | 0.76 | | 0.80 | 0.75 | 0.74 | 0.74 |  | **Abs 630**  **SD** | | | 1.95 | | 1.73 | | 1.43 | | | | | 1.14 | | 0.97 | | | 0.80 | | |
|  |  |  |  | 0.10 | 0.06 | | 0.12 | 0.06 | 0.07 | 0.09 |  |  |  |  | 0.02 | | 0.07 | | 0.06 | | | | | 0.05 | | 0.02 | | | 0.05 | | |
|  |  | **% Eradication %SD** | | 49.13 | 53.00 | | 50.44 | 53.14 | 53.86 | 54.22 |  | **% Eradication %SD** | | | 22.70 | | 31.21 | | 43.06 | | | | | 54.65 | | 61.36 | | | 68.07 | | |
|  |  |  |  | 12.50 | 7.38 | | 15.19 | 8.60 | 9.60 | 11.99 |  |  |  |  | 1.09 | | 4.08 | | 3.92 | | | | | 4.29 | | 2.26 | | | 6.29 | | |
|  |  | **Mean CFU**  **SD** | | 7.69E+08 | 3.97E+08 | | 4.20E+08 | 2.48E+08 | 1.52E+08 | 9.10E+08 |  | **Mean CFU**  **SD** | | | 1.22E+09 | | 1.68E+09 | | 1.60E+09 | | | | | 7.30E+08 | | 6.60E+08 | | | 3.52E+08 | | |
|  |  |  |  | 1.08E+08 | 2.67E+07 | | 5.04E+07 | 8.33E+06 | 3.50E+07 | 2.03E+08 |  |  |  |  | 6.00E+07 | | 3.08E+08 | | 2.25E+08 | | | | | 1.40E+08 | | 1.57E+08 | | | 3.83E+07 | | |
|  |  | **% Eradication CFU**  **%SD** | | 34.78 | 66.35 | | 64.37 | 78.93 | 87.13 | 22.81 |  | **% Eradication CFU**  **%SD** | | | 44.25 | | 23.52 | | 27.16 | | | | | 66.73 | | 69.92 | | | 83.97 | | |
|  |  |  |  | 14.09 | 6.72 | | 12.00 | 3.36 | 23.08 | 22.34 |  |  |  |  | 4.90 | | 18.37 | | 14.08 | | | | | 19.18 | | 23.74 | | | 10.90 | | |
| **Ceftriaxone** | | | | | | | | | | | | | | | | | | | | | | | | | | | | | | | |
| **Microorganism** | **Concentration (μg/ml)** | | | **2** | **5** | | **10** | **20** | **40** | **80** | **Concentration (μg/ml)** | | | | **2** | | **5** | | **10** | | | | | **20** | | **40** | | | **80** | | |
| ***Vibrio parahaemolyticus* (VP-87)** | **24**  **H**  **24°C** | **Abs 630**  **SD** | | 0.33 | 0.27 | | 0.24 | 0.21 | 0.20 | 0.45 | **72 H**  **30°C** | **Abs 630**  **SD** | | | | 1.08 | 0.85 | | 0.68 | | | | | 0.60 | | 0.51 | | | 0.36 | | |
|  |  |  |  | 0.01 | 0.01 | | 0.01 | 0.01 | 0.00 | 0.02 |  |  |  |  |  | 0.11 | 0.08 | | 0.04 | | | | | 0.05 | | 0.04 | | | 0.04 | | |
|  |  | **% Eradication %SD** | | 88.51 | 90.37 | | 91.58 | 92.49 | 93.10 | 84.12 |  | **% Eradication %SD** | | | | 55.93 | 65.53 | | 72.30 | | | | | 75.47 | | 79.38 | | | 85.29 | | |
|  |  |  |  | 3.77 | 3.07 | | 5.41 | 4.26 | 1.58 | 4.53 |  |  |  |  |  | 10.38 | 8.90 | | 6.56 | | | | | 8.77 | | 8.15 | | | 11.88 | | |
|  |  | **Mean CFU**  **SD** | | 5.01E+08 | 5.33E+08 | | 5.07E+08 | 5.40E+08 | 4.17E+08 | 4.43E+08 |  | **Mean CFU**  **SD** | | | | 4.12E+08 | 4.78E+08 | | 4.07E+08 | | | | | 2.59E+08 | | 2.39E+08 | | | 3.12E+08 | | |
|  |  |  |  | 2.35E+07 | 2.05E+07 | | 1.63E+07 | 2.67E+07 | 5.79E+07 | 3.14E+07 |  |  |  |  |  | 1.81E+07 | 3.85E+07 | | 2.37E+07 | | | | | 3.45E+07 | | 4.36E+07 | | | 6.17E+07 | | |
|  |  | **% Eradication CFU**  **%SD** | | 75.52 | 73.94 | | 75.24 | 73.62 | 79.64 | 78.34 |  | **% Eradication CFU**  **%SD** | | | | 74.92 | 70.93 | | 75.25 | | | | | 84.25 | | 85.46 | | | 81.03 | | |
|  |  |  |  | 4.68 | 3.85 | | 3.22 | 4.94 | 13.90 | 7.08 |  |  |  |  |  | 4.40 | 8.06 | | 5.83 | | | | | 13.31 | | 18.24 | | | 19.79 | | |
| ***Vibrio parahaemolyticus* (VP-275)** |  | **Abs 630**  **SD** | | 0.24 | 0.23 | | 0.21 | 0.18 | 0.17 | 0.14 |  | **Abs 630**  **SD** | | | | 1.22 | 1.03 | | 0.87 | | | | | 0.85 | | 0.75 | | | 0.55 | | |
|  |  |  |  | 0.00 | 0.01 | | 0.01 | 0.01 | 0.01 | 0.01 |  |  |  |  |  | 0.05 | 0.04 | | 0.05 | | | | | 0.02 | | 0.02 | | | 0.02 | | |
|  |  | **% Eradication %SD** | | 91.45 | 91.82 | | 92.64 | 93.64 | 93.89 | 95.01 |  | **% Eradication %SD** | | | | 57.29 | 64.08 | | 69.38 | | | | | 70.28 | | 73.73 | | | 80.60 | | |
|  |  |  |  | 1.99 | 2.18 | | 3.36 | 6.32 | 4.70 | 7.01 |  |  |  |  |  | 3.98 | 4.34 | | 5.77 | | | | | 2.05 | | 2.43 | | | 3.27 | | |
|  |  | **Mean CFU**  **SD** | | 5.50E+08 | 5.37E+08 | | 5.74E+08 | 6.06E+08 | 6.09E+08 | 5.17E+08 |  | **Mean CFU**  **SD** | | | | 3.50E+08 | 4.30E+08 | | 3.97E+08 | | | | | 3.72E+08 | | 3.64E+08 | | | 3.83E+08 | | |
|  |  |  |  | 4.08E+07 | 3.14E+07 | | 7.39E+07 | 6.85E+06 | 4.94E+07 | 3.09E+07 |  |  |  |  |  | 5.93E+07 | 3.67E+07 | | 4.71E+06 | | | | | 5.28E+07 | | 2.27E+07 | | | 3.09E+07 | | |
|  |  | **% Eradication CFU**  **%SD** | | 70.52 | 71.23 | | 69.21 | 67.54 | 67.36 | 72.30 |  | **% Eradication CFU**  **%SD** | | | | 78.31 | 73.35 | | 75.41 | | | | | 76.93 | | 77.41 | | | 76.24 | | |
|  |  |  |  | 7.42 | 5.85 | | 12.86 | 1.13 | 8.11 | 5.98 |  |  |  |  |  | 16.95 | 8.53 | | 1.19 | | | | | 14.18 | | 6.22 | | | 8.06 | | |
| ***Vibrio cholerae* (VC-112)** |  | **Abs 630**  **SD** | | 0.28 | 0.28 | | 0.28 | 0.28 | 0.28 | 0.24 |  | **Abs 630**  **SD** | | | | 1.87 | 1.43 | | 1.14 | | | | | 1.05 | | 0.86 | | | 0.64 | | |
|  |  |  |  | 0.02 | 0.02 | | 0.01 | 0.01 | 0.01 | 0.03 |  |  |  |  |  | 0.03 | 0.05 | | 0.02 | | | | | 0.04 | | 0.02 | | | 0.03 | | |
|  |  | **% Eradication %SD** | | 82.43 | 82.55 | | 82.74 | 82.44 | 82.73 | 85.00 |  | **% Eradication %SD** | | | | 25.84 | 43.18 | | 54.63 | | | | | 58.46 | | 65.97 | | | 74.76 | | |
|  |  |  |  | 5.80 | 6.25 | | 5.05 | 4.25 | 3.72 | 10.39 |  |  |  |  |  | 1.62 | 3.25 | | 1.52 | | | | | 3.86 | | 1.78 | | | 4.48 | | |
|  |  | **Mean CFU**  **SD** | | 4.24E+08 | 3.60E+08 | | 4.20E+08 | 2.30E+08 | 1.78E+08 | 3.59E+08 |  | **Mean CFU**  **SD** | | | | 5.47E+08 | 3.71E+08 | | 4.20E+08 | | | | | 2.33E+08 | | 3.24E+08 | | | 3.59E+08 | | |
|  |  |  |  | 2.91E+07 | 5.35E+07 | | 5.04E+07 | 2.67E+07 | 8.33E+06 | 3.29E+07 |  |  |  |  |  | 5.10E+07 | 4.22E+07 | | 5.04E+07 | | | | | 2.23E+07 | | 2.99E+07 | | | 3.29E+07 | | |
|  |  | **% Eradication CFU**  **%SD** | | 64.00 | 69.46 | | 64.37 | 80.49 | 84.87 | 69.56 |  | **% Eradication CFU**  **%SD** | | | | 75.09 | 83.09 | | 80.86 | | | | | 89.37 | | 85.22 | | | 83.65 | | |
|  |  |  |  | 6.86 | 14.87 | | 12.00 | 11.59 | 4.67 | 9.17 |  |  |  |  |  | 9.33 | 11.37 | | 12.00 | | | | | 9.55 | | 9.20 | | | 9.17 | | |

Legend- Summary table with the absorbance values at 630 nm, CFU, percentage of inhibition and eradication, and their respective standard deviation values obtained in the Biofilm tests carried out on 2 species of *Vibrio* (*Vibrio parahaemolyticus* and *Vibrio cholerae*), carried out with 2 commercial antibiotics from different chemical families (1. Tetracycline, 2. Ceftriaxone) and 2 plant extracts (Eucalyptus and Guava).
