# Supplementary material for: Eco-alternative treatments for Vibrio parahaemolyticus and V. cholerae biofilms from shrimp industry through Eucalyptus (Eucalyptus globulus) and Guava (Psidium guajava) extracts: A road for an Ecuadorian sustainable economy
Source: PLoS One. 2024 Aug 13;19(8):e0304126. doi: 10.1371/journal.pone.0304126 (PMC11321589; doi:10.1371/journal.pone.0304126)
Supplement: Attachment — Submitted filename: PONE-D-24-02362.pdf_Review comments.pdf [file pone.0304126.s010.pdf]

PLOS ONE

Eco-alternative treatments for Vibrio parahaemolyticus and V. cholerae biofilms from shrimp industry through Eucalyptus and Guava extracts: A road for an Ecuadorian sustainable economy

--Manuscript Draft--

**Manuscript Number:**	PONE-D-24-02362
**Article Type:**	Research Article
**Full Title:**	Eco-alternative treatments for Vibrio parahaemolyticus and V. cholerae biofilms from shrimp industry through Eucalyptus and Guava extracts: A road for an Ecuadorian sustainable economy
**Short Title:**	Eucalyptus and Guava eco-alternative treatments against Vibrio biofilms
**Corresponding Author:**	António Machado, PhD<br>Universidad San Francisco de Quito<br>Quito, Pichincha ECUADOR
**Keywords:**	Biofilms; Vibrio parahaemolyticus; Vibrio cholerae; Biomass assays; Fluorescence Microscopy; colony-forming units; Desirability analysis; alternative treatments; Green chemistry; sustainable economy
**Abstract:**	Understanding how environmental variables influence biofilm formation becomes relevant for managing Vibrio biofilm-related infections in shrimp production. Therefore, we evaluated the impact of temperature, time, and initial inoculum in the biofilm development of these two Vibrio species using a multifactorial experimental design. Planktonic growth inhibition and inhibition/eradication of Vibrio biofilms, more exactly V. parahaemolyticus (VP87 and VP275) and V. cholerae (VC112), were evaluated by Eucalyptus and Guava aqueous leaf extracts and compared to tetracycline and ceftriaxone. Preliminary results showed that the best growth conditions of biofilm development for V.parahaemolyticus were 24 h and 24 °C ($p <0.001$), while V.cholerae biofilms were 72 h and 30 °C ($p <0.001$). Multivariate linear regression ANOVA was applied using colony-forming unit (CFU) counting assays as a reference, and R-squared values were applied as goodness-of-fit measurements for biofilm analysis. Then, both plant extracts were analyzed with HPLC using double online detection by diode array detector (DAD) and mass spectrometry (MS) for the evaluation of their chemical composition, where the main identified compounds for Eucalyptus extract were cypellogin A, cypellogin B, and cypellocarpin C, while guavinoside A, B, and C compounds were the main compounds for Guava extract. For planktonic growth inhibition, Eucalyptus extract showed its maximum effect at 200 µg/mL with an inhibition of 75% ($p < 0.0001$) against all Vibrio strains, while Guava extract exhibited its maximum inhibition at 1600 µg/mL with an inhibition of 70% ($p < 0.0001$). Both biofilm inhibition and eradication assays were performed by the two conditions (24 h at 24 °C and 72 h at 30 °C) on Vibrio strains according to desirability analysis. Regarding 24 h at 24 °C, differences were observed in the CFU counting between antibiotics and plant extracts, where both extract plants demonstrated a higher reduction of viable cells when compared with both antibiotics at 8x, 16x, and 32x MIC values (Eucalyptus extract: 1600, 3200, and 6400 µg/mL; while Guava extract: 12800, 25600, and 52000 µg/mL). Concerning 72 h at 30 °C, results showed a less notorious biomass inhibition by Guava leaf extract and tetracycline. However, Eucalyptus extract significantly reduced the total number of viable cells within Vibrio biofilms from 2x to 32x MIC values (400-6400 µg/mL) when compared to the same MIC values of ceftriaxone (5-80 µg/mL), which was not able to reduce viable cells. Eucalyptus extract demonstrated similar results at both growth conditions, showing an average inhibition of approximately 80% at 400 µg/mL concentration for all Vibrio isolates ($p < 0.0001$). Moreover, eradication biofilm assays demonstrated significant eradication against all Vibrio strains at both growth conditions, but biofilm eradication values were substantially lower by all antibiotics and plant extracts. Both extract plants demonstrated higher reduction of viable cells when compared with both antibiotics at 8x, 16x, and 32x MIC values at both growth sets, where Eucalyptus extract at 800 µg/mL reduced 70% of biomass and 90% of viable cells for all Vibrio strains ($p < 0.0001$). Overall results suggested a viable alternative against vibriosis in the shrimp industry in Ecuador.

Powered by Editorial Manager® and ProduXion Manager® from Aries Systems Corporation

Order of Authors:	Nicolás Renato Jara-Medina
	Darío Fernando Cueva
	Ariana Cecibel Cedeño-Pinargote
	Arleth Gualle
	Daniel Aguilera-Pesantes
	Miguel Ángel Méndez, Phd
	Lourdes Orejuela-Escobar, PhD
	Eduardo Tejera, PhD
	António Machado, PhD
Additional Information:	
Question	Response
**Financial Disclosure**<br><br>Enter a financial disclosure statement that describes the sources of funding for the work included in this submission. Review the submission guidelines for detailed requirements. View published research articles from *PLOS ONE* for specific examples.<br><br>This statement is required for submission and **will appear in the published article** if the submission is accepted. Please make sure it is accurate.<br><br>**Funded studies**<br>Enter a statement with the following details:<br>• Initials of the authors who received each award<br>• Grant numbers awarded to each author<br>• The full name of each funder<br>• URL of each funder website<br>• Did the sponsors or funders play any role in the study design, data collection and analysis, decision to publish, or preparation of the manuscript?<br><br>Did you receive funding for this work?	Yes
Please add funding details.<br>as follow-up to "**Financial Disclosure**<br><br>Enter a financial disclosure statement that describes the sources of funding for the work included in this submission. Review	This work is supported by the COCIBA research budget to António Machado from Universidad San Francisco de Quito, under the Project ID: 17357 entitled "Alternative approaches for eliminating Biofilms" and the Project ID: 16801 entitled ''Characterization of single and mixed Biofilms''. The funders had no role in the study design, data collection, analysis, decision to publish, or preparation of the manuscript. The APC was funded by the Research Office of Universidad San Francisco de Quito

Powered by Editorial Manager® and ProduXion Manager® from Aries Systems Corporation

the submission guidelines for detailed requirements. View published research articles from *PLOS ONE* for specific examples.<br><br>This statement is required for submission and **will appear in the published article** if the submission is accepted. Please make sure it is accurate.<br><br>**Funded studies**<br>Enter a statement with the following details:<br>• Initials of the authors who received each award<br>• Grant numbers awarded to each author<br>• The full name of each funder<br>• URL of each funder website<br>• Did the sponsors or funders play any role in the study design, data collection and analysis, decision to publish, or preparation of the manuscript?<br><br>Did you receive funding for this work?"	(USFQ).
Please select the country of your main research funder (please select carefully as in some cases this is used in fee calculation).<br>as follow-up to "**Financial Disclosure**<br><br>Enter a financial disclosure statement that describes the sources of funding for the work included in this submission. Review the submission guidelines for detailed requirements. View published research articles from *PLOS ONE* for specific examples.<br><br>This statement is required for submission and **will appear in the published article** if the submission is accepted. Please make sure it is accurate.	ECUADOR - EC

Powered by Editorial Manager® and ProduXion Manager® from Aries Systems Corporation

**Funded studies**<br>Enter a statement with the following details:<br>• Initials of the authors who received each award<br>• Grant numbers awarded to each author<br>• The full name of each funder<br>• URL of each funder website<br>• Did the sponsors or funders play any role in the study design, data collection and analysis, decision to publish, or preparation of the manuscript?<br><br>Did you receive funding for this work?"	
**Competing Interests**<br><br>Use the instructions below to enter a competing interest statement for this submission. On behalf of all authors, disclose any competing interests that could be perceived to bias this work—acknowledging all financial support and any other relevant financial or non-financial competing interests.<br><br>This statement is required for submission and **will appear in the published article** if the submission is accepted. Please make sure it is accurate and that any funding sources listed in your Funding Information later in the submission form are also declared in your Financial Disclosure statement.<br><br>View published research articles from *PLOS ONE* for specific examples.	The authors have declared that no competing interests exist.

Powered by Editorial Manager® and ProduXion Manager® from Aries Systems Corporation

**NO authors have competing interests**<br><br>Enter: *The authors have declared that no competing interests exist.*<br><br>**Authors with competing interests**<br><br>Enter competing interest details beginning with this statement:<br><br>*I have read the journal's policy and the authors of this manuscript have the following competing interests: [insert competing interests here]*<br><br>* typeset	
**Ethics Statement**<br><br>Enter an ethics statement for this submission. This statement is required if the study involved:<br><br>• Human participants<br>• Human specimens or tissue<br>• Vertebrate animals or cephalopods<br>• Vertebrate embryos or tissues<br>• Field research<br><br>Write "N/A" if the submission does not require an ethics statement.<br><br>General guidance is provided below. Consult the submission guidelines for detailed instructions. **Make sure that all information entered here is included in the Methods section of the manuscript.**	N/A

**Format for specific study types**<br><br>**Human Subject Research (involving human participants and/or tissue)**<br>• Give the name of the institutional review board or ethics committee that approved the study<br>• Include the approval number and/or a statement indicating approval of this research<br>• Indicate the form of consent obtained (written/oral) or the reason that consent was not obtained (e.g. the data were analyzed anonymously)<br><br>**Animal Research (involving vertebrate animals, embryos or tissues)**<br>• Provide the name of the Institutional Animal Care and Use Committee (IACUC) or other relevant ethics board that reviewed the study protocol, and indicate whether they approved this research or granted a formal waiver of ethical approval<br>• Include an approval number if one was obtained<br>• If the study involved *non-human primates*, add *additional details* about animal welfare and steps taken to ameliorate suffering<br>• If anesthesia, euthanasia, or any kind of animal sacrifice is part of the study, include briefly which substances and/or methods were applied<br><br>**Field Research**<br><br>Include the following details if this study involves the collection of plant, animal, or other materials from a natural setting:<br>• Field permit number<br>• Name of the institution or relevant body that granted permission	
**Data Availability**<br><br>Authors are required to make all data underlying the findings described fully available, without restriction, and from the time of publication. PLOS allows rare exceptions to address legal and ethical concerns. See the PLOS Data Policy and FAQ for detailed information.	Yes - all data are fully available without restriction

A Data Availability Statement describing where the data can be found is required at submission. Your answers to this question constitute the Data Availability Statement and **will be published in the article**, if accepted.<br><br>**Important:** Stating 'data available on request from the author' is not sufficient. If your data are only available upon request, select 'No' for the first question and explain your exceptional situation in the text box.<br><br>Do the authors confirm that all data underlying the findings described in their manuscript are fully available without restriction?	
**Describe where the data may be found in full sentences. If you are copying our sample text, replace any instances of XXX with the appropriate details.**<br><br>• If the data are **held or will be held in a public repository**, include URLs, accession numbers or DOIs. If this information will only be available after acceptance, indicate this by ticking the box below. For example: *All XXX files are available from the XXX database (accession number(s) XXX, XXX.*).<br>• If the data are all contained **within the manuscript and/or Supporting Information files**, enter the following: *All relevant data are within the manuscript and its Supporting Information files.*<br>• If neither of these applies but you are able to provide **details of access elsewhere**, with or without limitations, please do so. For example:<br><br>*Data cannot be shared publicly because of [XXX]. Data are available from the XXX Institutional Data Access / Ethics Committee (contact via XXX) for researchers who meet the criteria for access to confidential data.*<br><br>*The data underlying the results presented in the study are available from (include the name of the third party*	All data may be found in the manuscript and supplementary section.

and contact information or URL).<br>• This text is appropriate if the data are owned by a third party and authors do not have permission to share the data.<br><br>* typeset	
Additional data availability information:	

Powered by Editorial Manager® and ProduXion Manager® from Aries Systems Corporation

Manuscript

Click here to access/download;Manuscript;Manuscript_Vibrio_PlosONE-

Eco-alternative treatments for *Vibrio parahaemolyticus* and *V. cholerae* biofilms from shrimp industry through Eucalyptus and Guava extracts: A road for an Ecuadorian sustainable economy

Nicolás Renato Jara-Medina[1], Dario F. Cueva[1], Ariana Cecibel Cedeño-Pinargote[1], Arleth Gualle[2], Daniel Aguilera-Pesantes[3], Miguel Ángel Méndez[2], Lourdes Orejuela-Escobar[2,4,5], Eduardo Tejera[6]*, and António Machado[1]*

[1] Universidad San Francisco de Quito USFQ, Colegio de Ciencias Biológicas y Ambientales COCIBA, Instituto de Microbiología, Laboratorio de Bacteriología, Calle Diego de Robles y Pampite, Quito, 170901, Ecuador. E-mail: nrjara@alumni.usfq.edu.ec (N.R.J.-M.); dcueva@estud.usfq.edu.ec (D.F.C.);); accedenop@alumni.usfq.edu.ec (A.C.C.-P.); amachado@usfq.edu.ec (A.M.)

[2] Department of Chemical Engineering, Universidad San Francisco de Quito USFQ, Diego de Robles s/n y Av. Interoceánica, Quito 170157, Ecuador. E-mail: arleth.gualle@alumni.usfq.edu.ec (A.G.); mmendez@usfq.edu.ec (M.A.-M.); lorejuela@usfq.edu.ec (L.O.-E.)

[3] Clydent S.A, Research, Development and Strategic Planning Department, Mirtos 617 entre Ficus y Av. De Las Monjas, Guayaquil 090507, Ecuador. E-mail: proyectos.clydent@gmail.com (D.A.-P.)

[4] Instituto de Investigaciones Biológicas y Ambientales BIOSFERA, Universidad San Francisco de Quito, Diego de Robles s/n y Av. Interoceánica, Quito 170157, Ecuador.

[5] Instituto de Investigaciones en Biomedicina, Universidad San Francisco de Quito, Diego de Robles s/n y Av. Interoceánica, Quito 170157, Ecuador.

[6] Facultad de Ingeniería y Ciencias Agropecuarias Aplicadas, Grupo de Bioquimioinformática, Universidad de Las Américas (UDLA), De Los Colimes esq, Quito 170513, Ecuador. Email: eduardo.tejera@udla.edu.ec (E.T.)

*** Correspondence:**
Corresponding author
amachado@usfq.edu.ec

Co-corresponding author
eduardo.tejera@udla.edu.ec

Running title: Eucalyptus and Guava eco-alternative treatments against *Vibrio* biofilms

Keywords: Biofilms1; *Vibrio parahaemolyticus*2; *Vibrio cholerae*3; Biomass assays4; Fluorescence microscopy5; Colony-forming Units6; Desirability analysis7; Alternative treatments8; Green chemistry9; Sustainable economy10.

Words: 8,874
Figures: 8
Tables: 1
Supplementary files: 11

Abstract

Understanding how environmental variables influence biofilm formation becomes relevant for managing *Vibrio* biofilm-related infections in shrimp production. Therefore, we evaluated the impact of temperature, time, and initial inoculum in the biofilm development of these two *Vibrio* species using a multifactorial experimental design. Planktonic growth inhibition and inhibition/eradication of *Vibrio* biofilms, more exactly *V. parahaemolyticus* (VP87 and VP275) and *V. cholerae* (VC112), were evaluated by Eucalyptus and Guava aqueous leaf extracts and compared to tetracycline and ceftriaxone. Preliminary results showed that the best growth conditions of biofilm development for *V. parahaemolyticus* were 24 h and 24 °C (p <0.001), while *V. cholerae* biofilms were 72 h and 30 °C (p <0.001). Multivariate linear regression ANOVA was applied using colony-forming unit (CFU) counting assays as a reference, and R-squared values were applied as goodness-of-fit measurements for biofilm analysis. Then, both plant extracts were analyzed with HPLC using double online detection by diode array detector (DAD) and mass spectrometry (MS) for the evaluation of their chemical composition, where the main identified compounds for Eucalyptus extract were cypellogin A, cypellogin B, and cypellocarpin C, while guavinoside A, B, and C compounds were the main compounds for Guava extract. For planktonic growth inhibition, Eucalyptus extract showed its maximum effect at 200 µg/mL with an inhibition of 75% (p < 0.0001) against all *Vibrio* strains, while Guava extract exhibited its maximum inhibition at 1600 µg/mL with an inhibition of 70% (p < 0.0001). Both biofilm inhibition and eradication assays were performed by the two conditions (24 h at 24 °C and 72 h at 30 °C) on *Vibrio* strains according to desirability analysis. Regarding 24 h at 24 °C, differences were observed in the CFU counting between antibiotics and plant extracts, where both extract plants demonstrated a higher reduction of viable cells when compared with both antibiotics at 8x, 16x, and 32x MIC values (Eucalyptus extract: 1600, 3200, and 6400 µg/mL; while Guava extract: 12800, 25600, and 52000 µg/mL). Concerning 72 h at 30 °C, results showed a less notorious biomass inhibition by Guava leaf extract and tetracycline. However, Eucalyptus extract significantly reduced the total number of viable cells within *Vibrio* biofilms from 2x to 32x MIC values (400-6400 µg/mL) when compared to the same MIC values of ceftriaxone (5-80 µg/mL), which was not able to reduce viable cells. Eucalyptus extract demonstrated similar results at both growth conditions, showing an average inhibition of approximately 80% at 400 µg/mL concentration for all *Vibrio* isolates (p < 0.0001). Moreover, eradication biofilm assays demonstrated significant eradication against all *Vibrio* strains at both growth conditions, but biofilm eradication values were substantially lower by all antibiotics and plant extracts. Both extract plants demonstrated higher reduction of viable cells when compared with both antibiotics at 8x, 16x, and 32x MIC values at both growth sets, where Eucalyptus extract at 800 µg/mL reduced 70% of biomass and 90% of viable cells for all *Vibrio* strains (p < 0.0001). Overall results suggested a viable alternative against vibriosis in the shrimp industry in Ecuador.

1 Introduction

The aquaculture industry according to the Food and Agricultural Organization (FAO) is one of the industries with the highest production growth with 9.4% per year [1]. However, it has been affected by aquatic diseases mainly produced by various species of *Vibrio* [2], which generates economic losses for all crustacean-producing countries. According to Global Outlook for Aquaculture Leadership (GOAL) of 2019, Ecuador is the country with the largest production and export of shrimp in the world, and, in the last decade, it has had a rapid growth of approximately 16% [3]. Additionally, it represents 19% of the country's exports, generating 5 billion dollars in 2021. The shrimp industry is located in

the coastal zone of the country, distributed mainly in 5 provinces, with Guayas being the province with the highest production (60%), followed by El Oro, Esmeraldas, Manabí, and Santa Elena [4]. However, one of the main draw flaws of shrimp aquaculture industry is *Vibrio*-related infections [5]. Probiotics are usually chosen as the best alternatives to these antimicrobial agents, and they act as natural immune enhancers, which provoke the disease resistance in shrimp aquaculture [6,7]. However, green chemistry constitutes nowadays a pathway for a sustainable economy and future to the efficient and clean transformation of renewable raw materials into functional chemicals [8]. Eucalyptus and Guava leaves constitute raw materials with well-known antimicrobial activity [9,10] and a new potential sustainable economy for Ecuadorian market.

Vibrio spp. is a curved rod gram-negative bacterium, facultative aerobic and facultative anaerobic rods, which has a single flagellum [11]. It is generally found in warm salty aquatic environments such as seawater and estuaries. This genus is also found in different conditions, survives at 5-45 °C, and achieves substantial growth when seawater temperatures are over 14-19 °C [12,13]. It is an opportunistic pathogenic bacterium for marine animals as well as for people [14]. In aquaculture, this pathogen can be a big issue causing a deadly disease for shrimp and an opportunistic gastrointestinal infection in humans [15]. In nature, *Vibrio* species can usually be found in multispecies communities that form biofilms, which gives them greater resilience and adaptability [16]. Biofilms are microbial consortia that are enveloped and protected by an extracellular polymeric substance (EPS) matrix, being the most common bacterial community found in nature. Biofilms are formed due to quorum sensing (QS), which is a communication system used by bacteria that produce changes in gene expression driven by a message (c-di-GMP) [17]. This causes a phenotypic shift with the reduction of the motor activity of the flagella and regulates the expression of surface proteins for the union and synthesis of the extracellular matrix [18[illegible]

The main diseases that affect shrimp are early mortality shrimp-acute hepatopancreatic necrosis disease (EMS – AHPND) and causes 100% of mortality, while vibriosis causes 70% of mortality but damages the shrimp´s oral cavity and appendages [19]. These diseases are caused by pathogenic *Vibrio* species, being *Vibrio alginolyticus*, *V. parahaemolyticus*, *V. cholerae*, and *V. vulnificus* the main pathogens isolated [16[illegible]n marine biotic or abiotic surface under appropriate growth conditions, *Vibrio* sp. biofilms can function as a source of pathogenic bacteria with 10 – 1000 times more resistance against the hygiene treatment than planktonic counter parts. In a study reported by Odeyemi (2016), the authors evaluated the prevalence of *V. parahaemolyticus* based on 48 published studies and reported a 48.3% of its presence in shrimp, especially in uneven seafood surface areas where pits and edges provided protection for biofilm communities. According to international standards, there are certain antibiotics such as chloramphenicol, tetracyclines, sulphonamides, and quinolones, which cannot be used due to the presence of antibiotic residues in the shrimp that could be ingested by the consumers and therefore they are not allowed in the shrimp production [11,20]. Some alternative strategies to control *Vibrio* species and their biofilm formation is using probiotics and natural products in mature ecosystems [2,6[illegible] In addition to preventing the growth and spread of *Vibrio* species due to competition, certain probiotic bacteria are also able to perform quorum quenching, which is the QS inhibition and thus inhibiting biofilm formation [7]. However, when biofilms have already been established, these strategies don´t work well. Therefore, it is important to analyze the biofilm life cycle of the main pathogenic *Vibrio* species identifying the best growth conditions to further evaluate suitable treatments against *Vibrio*-related biofilms.

So, the present work aimed to evaluate the biofilm formation of *V. parahaemolyticus* and *V. cholerae* and characterize their biofilm production through different *in vitro* conditions. This study characterized

Vibrio sp. biofilms at different temperature conditions (24 and 30 °C), during a range of time (24, 48, and 72 h), and at different concentrations of initial inoculum (1E+7 and 1E+8 colony-forming units (CFU)/mL); biomass growth assays (optical density measurement at 630 nm using crystal violet staining and PBS suspension); CFU counting assay; and, fluorescence microscopy (FM) analysis. After the establishment of the best experimental conditions for *Vibrio*-related biofilms, Guava (*Psidium guajava L.)* and Eucalyptus (*Eucalyptus* sp.*)* aqueous leaf extracts were further evaluated as eco-alternative treatments for *Vibrio parahaemolyticus* and *V. cholerae* biofilms through biofilm inhibition and eradication assays and compared to standard antibiotic treatments, more exactly tetracycline and ceftriaxone.

2 Materials and Methods

2.1 Bacterial isolates and growth conditions

Vibrio cholerae and *V. parahaemolyticus* isolates from the microbial collection of the Institute of Microbiology Universidad San Francisco de Quito (IM-USFQ), designated as IMUSFQ-VC112 and IMUSFQ-VP 87, respectively, were selected for the present study. These *Vibrio* isolates were previously recovered from El Oro, Santa Elena, and Guayas and identified through DNA sequences at multiple loci and biochemical properties at the Clydent S.A laboratory (https://clydent.com/) and IM-USFQ. These isolates were stored at -80 °C or -20 °C, and 24 h before each assay, a new culture in trypticase soy agar (TSA; Dipco Cía. Ltda., Quito, Ecuador) supplemented with NaCl 1% (w/v; see Supplemental Material) was made to avoid natural mutants [21,22]. After 24 h of growth culture at 30 °C, bacterial cells were harvested and suspended in phosphate-buffered saline (PBS) to obtain a cellular density equal to 1E+8 colony-forming units (CFU) per mL using 0.5 McFarland turbidity standard and then a second dilution 1:10 was prepared to obtain another concentration of 1E+7 CFU/mL gathering two initial inoculum concentrations for biofilm assays. The optical density of both *Vibrio* inoculums was measured at 630 nm using a UV-Vis spectrometer (GENESYS™ 20 Thermo Scientific™, Waltham, Massachusetts, USA), ensuring the identical concentration in all assays (see Supplemental Material).

2.2 Plant extract and chemical analysis

Fresh guava (*Psidium guajava L.)* and eucalyptus (*Eucalyptus* sp.*)* leaf samples were obtained from the local market in Quito, Ecuador. The raw materials were washed and dried in an oven at 40 ºC for 24 h. The dry leaves were then ground and sieved to a particle size of less than 250 microns. Soxhlet extractions were carried out with 10 grams of dry biomass and 300 mL of distilled water for 3 h. The liquid extracts were concentrated in a rotary evaporator and stored at -20ºC and then lyophilized. The dry extracts were dissolved in 0.5 mL of 0.1 % formic acid/acetonitrile (70:30, v/v), filtered through a 0.22 µm disposable LC filter disk (RephiLe Bioscience Ltd) and analyzed with HPLC using double online detection by diode array detector (DAD) and mass spectrometry (MS) following a previously described methodology [23]. The system consisted in a Vanquish (Thermo Fisher Scientific) fitted with a binary pump and DAD coupled with an LTQ-XL (Thermo Fisher Scientific) controlled by Xcalibur Software (Thermo Scientific™, Waltham, Massachusetts, USA). An Accucore Vanquish C18 column (1.5 µm, 100 × 2.1 mm) thermostated at 35 °C was used as stationary phase, while for elution a solution of 0.1% formic acid and acetonitrile was used in gradient. Double line detection was carried out in DAD at 280, 330 and 370 nm and MS operated in positive ion mode. The dependent data analysis (DDA) was done on the 5 most intense ions with normalized collision energy of 35. Spectra between m/z 100 and m/z 1500 were recorded. The Electrospray Ionization (ESI) conditions were as follow:

capillary temperature of 275 °C, source voltage and capillary voltage of 5Kv and -35 V, respectively and tubelens –200. The peak tentative identification was done using MzCloud (https://www.mzcloud.org/) and Global Natural Products Social molecular networking (GNPS, http://gnps.ucsd.edu) databases as well as the scientific literature.

2.3 Biofilm assays

The two previous inoculums were evaluated in the present assays of *Vibrio*-related biofilm formation. Each inoculum of *V. cholerae* or *V. parahaemolyticus* was centrifuged at 10000 rpm, for 20 minutes and the pellet was resuspended in sterile trypticase soy broth (TSB; Dipco Cía. Ltda., Quito, Ecuador) supplemented with NaCl 1% (w/v). In each well of the 6-well plate containing a sterile coverslip, 3 mL of primary biofilm inoculum was added. Also, blank or sterility control was prepared on the same plate, which only contained a cover slip in a sterile medium [21]. Plates containing different inoculums and *Vibrio* species were also incubated at two different temperatures (24 and 30 °C) and for different periods (24, 48, and 72 h) under static conditions, replacing the medium in each well with 3 mL of fresh medium every 24 h after the biofilm samples were washed with 3 mL of PBS [24]. Each condition setting (*Vibrio* species, initial inoculum, temperature, and growth culture period) was performed with at least six assays, and, in each assay, five biofilm samples were separately prepared. All biofilm assays were performed on different days

2.4 Biomass quantification

To screen the ability of each strain of *V. cholerae* and *V. parahaemolyticus* to form a biofilm, we evaluated biomass formation using an optical density (OD) assay with crystal violet (CV) staining and phosphate-buffered saline (PBS) suspension, as prepared in our previous study [21]. Briefly, each optical density assay is described below.

2.4.1 Crystal violet staining

After a certain period of growth (24, 48, and 72 h), the biofilm samples on coverslips were carefully washed four times by submerging each coverslip in a well with 3 mL of sterile PBS. Then, the coverslips containing the biofilm sample were transferred to a clean 6-well plate and stained with 3 mL of crystal violet 1% (v/v) for 30 minutes, and the excess staining was carefully removed from the wells. 3 mL of alcohol 96% (v/v) were placed into each well for 5 minutes for fixation and then each coverslip containing the fixed biofilm sample was transferred into a sterile plastic flask with 3 mL of alcohol 96% (v/v), and vortexed at maximum velocity for 15 minutes to ensure the biofilm removal of the coverslip into the alcohol solution. Finally, 200 µL of each biofilm sample was placed in a 96-well plate and read in the ELISA Elx808 spectrophotometer (BioTek, Winooski, USA) at 630 nm. All biofilm samples and sterility controls (coverslips in medium with no inoculum) were included in the OD measurements on the 96-well plate.

2.4.2 Phosphate-buffered saline suspension

Likewise, the second set of biofilm samples was carefully washed four times with 3 mL of sterile PBS. Then each coverslip containing the biofilm sample was transferred in a sterile plastic flask with 3 mL of sterile PBS, and vortexed at maximum velocity for 15 minutes to ensure the biofilm removal of the coverslip into the PBS solution. For each sample, 200 µL of the previous suspension were added in a 96-well plate and read in the ELISA Elx808 spectrophotometer at 630 nm. As previously mentioned, all biofilm samples and sterility controls (coverslips in medium with no inoculum) were measured. The remaining PBS suspension was used for viability quantification assays.

2.5 Viability quantification

2.5.1 Colony-forming units counting assays

To enumerate culturable sessile cells, a colony-forming unit (CFU) counting assay was used. At least three individual PBS suspensions of each biofilm sample (resuspended *Vibrio* sp. biofilm of the initial coverslip) were used in a serial tenfold dilution, by adding 100 µL of sample to 900 µL of sterile PBS supplemented with NaCl 1% (w/v). Each dilution was thoroughly vortexed and pipette tips were changed before the next dilution or experimental step. The tested dilutions included (1E-5 –1E-7) and each dilution was plated on TSA supplemented with NaCl 1% (w/v) by triplicate results per biofilm sample. All plates were incubated for 24 h at 30 °C, after which colonies were counted. The experiments were performed at the same time as biomass experiments; thus, three CFU assays per dilution were available for analysis, and data was collected. For statistical analysis, the dilution with a growth between 25 and 150 CFU was chosen according to our previous study [21].

2.5.2 LIVE/DEAD assays

After the evaluation of CFU counting and biomass quantification assays, all time samples (24, 48, and 72 h) were chosen to be analyzed by fluorescence staining. After each biofilm formation assay in 6-well plates, any remaining medium in the wells was removed and coverslips were transferred to a new and sterile 6-well plate. A working solution of fluorescent stains was prepared by adding 1.0 mL of SYTO® 9 stain and 10 µL of propidium iodide (PI) stain (FilmTracer™ LIVE/DEAD® Biofilm Viability Kit, Invitrogen, Carlsbad, California, USA), mixed in the proportion 1:100 of PI/SYTO-9, into 10 mL of filter-sterilized water in a foil-covered container. This live/dead working solution was stored at -20 °C. 200 µL of the live/dead working solution were added onto each coverslip (biofilm sample), gently so it would not disturb the biofilm. All samples were then incubated for 15-30 min at room temperature, protected from light, before being rinsed with 200 µL of PBS. Finally, after the water evaporation of the coverslips, each coverslip was then placed face up onto a clean, dry microscope slide and a drop of mounting medium was added (ProLong Gold Antifade, ThermoFisher Scientific, Massachusetts, USA). An autoclavable 22-mm diameter glass coverslip (Dipco Cía. Ltda., Quito, Ecuador) was used to fix the sample in place. Samples were stored protected from light at room temperature (25 °C) until epifluorescence microscopy analysis, which it was performed within the first 3 h [21,25,26].

2.5.3 Fluorescence microscopy

Fluorescence microscopy (FM) was carried out using an Olympus BX50 microscope (Olympus Corporation, Tokyo, Japan) equipped with a 100x oil immersion objective. Images were captured with AmScope Digital Camera MU633-FL (AmScope, California, USA) and digitalized with AmScope software version 1.2.2.10. As previously described by Cabezas-Mera and colleagues [27], for counting purposes at least 12 images were taken per sample on the 22-mm diameter glass coverslip at random locations. In addition, both *V. parahaemolyticus* and *V. cholerae* exhibited mature biofilms with multilayered growth over time, due to this problem the biofilm sample was resuspended in 10 mL of PBS and vortexed at maximum velocity for 5 minutes. Then, a 30 µL aliquot was placed on a coverslip, thus facilitating the cell area count in each biofilm and comparing it between *Vibrio* species under different growth conditions, as previously reported [28]. For more reproducible result presentation, cell counts were given per cm^2. The number of *Vibrio* cells was counted from each field to obtain the average number of cells over the total area of the abiotic surface. Briefly, the coverslip area (4.84E +

8 µm^2) was divided by the area of the photograph picture (12880 µm^2) and the average of live and dead cells from microscopic fields was multiplied by the previous ratio, obtaining the total number of cells over the abiotic glass surface, as previously described [29]. These results were expressed as the number of cells ± standard deviation per cm^2 (N. of cells/cm^2 ± SD). In FM analysis, the percentages of dead and alive cells within images were measured through ImageJ version 1.57 by Fiji [30] using the macros Biofilms Viability checker proposed by Mountcastle and colleagues [31] and the plugin MorphoLibJ [32]; while, the total cells counting images were processed by a sequence of modules forming a pipeline designed for this purpose in Cell Profiler software [33] an open-source software version 4.2.1 (available from the Broad Institute at www.cellprofiler.org), the applied pipeline of which can be revised in Supplemental Information.

2.6 Antimicrobial activity

After the optimization and evaluation of biofilm growth conditions, the antimicrobial activity of the plant extracts (Eucalyptus and Guava leaves) was evaluated and compared with well-known antibiotics (tetracycline and ceftriaxone) against *Vibrio* pathogens through the Microdilution method for minimum inhibitory concentration (MIC) assays and biofilm inhibition and eradication assays. The antimicrobial activity was evaluated by biomass quantification and CFU counting assays.

2.6.1 Minimum inhibitory concentration (MIC)

Microdilution method for minimum inhibitory concentration (MIC) assays were conducted accordingly to Wiegand and colleagues [34], under the Clinical Laboratory Standards Institute (CLSI) guidelines. In MIC assays, serial dilution was prepared starting with 80 µg/mL antibiotics (tetracycline and ceftriaxone) and 1600 µg/mL plant extracts (Eucalyptus and Guava leaves) powder in 190 µl of TSB (Dipco Cía. Ltda., Quito, Ecuador) supplemented with NaCl 1% (w/v) in 96-well plates [35]. Then, the remaining wells of the plate were set up with the diluted concentrations of 40, 20, 10, 5, and 2 µg/mL of tetracycline and ceftriaxone, while 800, 400, 200, 100, and 50 µg/mL of Eucalyptus and Guava extracts within the total volume of 200 µL of TSB broth with 1%(w/v) NaCl in the 96-well plate. All wells were prepared with a final concentration of 1E+5 CFU/mL of *Vibrio* inoculum, excepting negative controls, and the 96-wells plate was incubated for 18-24 h at 37°C [34]. A row of TSB with 1% (w/v) NaCl plus pathogen inoculum was used as culture growth control (or positive control), and a row of wells were filled with medium without inoculum was applied to sterility control (negative control). Tetracycline and ceftriaxone (Sigma-Aldrich, St. Louis, MO, USA) were used as a reference control [36]. The MIC values were firstly evaluated by the unaided eye and then measured with the spectrophotometer Biotek Instruments ELx808IU at 630 nm (OD630) by comparing the results obtained with the negative control. The MIC was defined as the lowest concentration of antibiotic or plant extract that inhibited the *Vibrio* growth after overnight incubation. All the assays were independently performed in triplicate in different days.

2.6.2 Biofilm inhibition and eradication assays

An overnight culture of the *Vibrio* species was grown and an inoculum of 1E+7 CFU/mL in sterile TSB supplemented with NaCl 1% (w/v) was prepared as previously described and 190 µL were added to the 96-well plate. Next, the wells were supplemented with 10 µL of concentrated 20x antibiotics (tetracycline and ceftriaxone) or plant extracts (Eucalyptus and Guava) achieving 200 µL of the final volume in each well setting up 1x to 32x MIC values [37]. Plates were then incubated under two different conditions at 24 h at 24 °C and 72 h at 30 °C under aerobic conditions to evaluate the biofilm inhibition. Meanwhile, in biofilm eradication assays, 200 µL of media plus inoculum was added in each well, as well as the negative control (only media), and the 96-well plate was incubated under two

different conditions at 24 h at 24 °C and 72 h at 30 °C under aerobic conditions for the biofilm formation. After the establishment of mature biofilms, the TSB with NaCl 1% (w/v) was gently removed from the wells, followed by a gentle wash with PBS (pH 7.4), and biofilm samples were then supplemented with 1x to 32 x MIC values of antibiotics or plant extracts (Eucalyptus and Guava leaves). Plates were again incubated for an additional 24 h at the same previous temperature (24 or 30 °C) under aerobic conditions. Again, in both biofilm assays, a row of TSB with 1% (w/v) NaCl plus pathogen inoculum without any antibiotic or plant extract was used as growth control (or positive control), and a row of wells were filled with medium without inoculum and antibiotic/plant extract was applied to control of sterility (negative control). After finishing the biofilm inhibition and eradication assays, all samples were evaluated for the antimicrobial activity of the antibiotics and plant extracts through the methodologies previously described (biomass quantification and CFU counting assays). Likewise, all the assays were independently performed in triplicate in different days.

2.7 Statistical analysis

2.7.1 Response surface methodology: Experimental design of biofilm formation

A multilevel multifactorial model was chosen due to the difference in the number of levels between factors to be analyzed. The present model was composed of a 2 x 2 x 2 x 3 factorial design without midpoints. In the experiments, the levels of the factors for the formation of biomass and viability varied, these were carried out according to the multifactorial model. Therefore, only six tests were necessary to determine the influence of the factors on the formation of biomass and viability. In the multifactorial model, there were their levels to be studied (2 *Vibrio* species, 2 temperatures, 2 initial inoculums and 3 times). The obtained data allow us to evaluate a second order response surface with an adjusted model illustrating the curvature of the effects of the factors and their interactions (Equation 1).

$$y = \beta_0 + \sum_{i=1}^{k} \beta_i x_i + \sum_{i=1}^{k} \sum_{j=1}^{k} \beta_{ij} x_i x_j + \sum_{i=1}^{k} \beta_{ij}\, x_i^2 + \in$$

(Equation 1)

2.7.2 Response surface methodology: Experimental design of biofilm treatment with antibiotics and plant extracts

For the experimental design of the treatments against biofilms, the two best conditions for biofilm formation were selected. Subsequently, the analysis of composite desirability was carried out on the experimental design of the formation of biofilms. A multilevel multifactor model also performed. This model was composed of a 2 x 3 x 4 factorial design without midpoints. In the experiments, the levels of the factors for the formation of biomass and viability varied and so these data were carried out according to the multifactorial model. In the multifactorial model, 2 optimal training conditions, 3 *Vibrio* species and 4 treatments were evaluated. The obtained data allow us again to analyze a second order response surface with an adjusted model evidencing the curvature of the effects of the factors and their interactions (Equation 1).

2.7.3 ANOVA Multifactorial statistical analysis

All data in the present study was obtained from at least triplicate assays done on different days. In the case of biomass growth, CFU counting, and total cell or live/dead cell counting, each assay was performed using five samples per assay. All data results were carried out to obtain the mean, minimum, maximum, and standard deviation (SD). The normality data set was analyzed using the Anderson-Darling, Ryan-Joiner, and Kolmogorov-Smirnov normality tests. If the data had a non-normal distribution, nonparametric tests were used. The Johnson data transformation method determines the best value for lambda (λ), thus obtaining the best type of transformation from non-parametric to parametric data. Once the data were transformed, a multivariate ANOVA analysis was performed and p-values <0.05 were considered significant. To compare the means between the factors of each variable or condition, the Tukey and Fisher tests were performed with an alpha of 0.05 ($\alpha = 0.05$). Likewise, the values obtained between the factors of each variable with $p < 0.05$ are considered significant. Finally, an optimization analysis of the model was carried out based on the composite desirability function to obtain the best combinations of levels and variables evaluating the best growth conditions for the development of biofilms. All data analysis was performed in Minitab version 20 [38].

2.7.4 Multi-objective optimization and general desirability function

The desirability model states that for each response (Y_i), a desirability function ($d_i(Y_i)$) is necessary considering whether to maximize, minimize or achieve an objective [39,40]. This approach was originally proposed by Harrington (1965) but further improved by Derringer and Suich (1980), where an estimated response is transformed into an unscaled value known as desirability [41]. The data values are assigned between 0 and 1 of these transformed responses d_i, which can have many different forms. Regardless of the form, d_i=0 represents a completely undesirable value of and d_i=1 symbolizes a completely desirable or ideal response value.

For the desirability transformation, values can be used unilaterally to maximize or minimize Y_i (Equation 2), being the unilateral transformation of maximization used for the desirability analysis; meanwhile, the two-sided transformation is applied to obtain the target value T_i for Y_i where L_i and U_i correspond to the lower and upper limits of the studied response, respectively.

$$d_i = \begin{cases} 0 & \text{if} \quad \hat{Y}_i \leq L_i \\ \left[\dfrac{\hat{Y}_i - L_i}{T_i - L_i}\right]^s & \text{if} \quad L_i < \hat{Y}_i < T_i \\ 1 & \text{if} \quad \hat{Y}_i \geq T_i = U_i \end{cases}$$

(Equation 2)

The exponent s corresponds to the weighted factor, which indicate the importance of each response to reach the target value T_i. For s = 1, the stated desirability function increases linearly up to Ti.

Therefore, individual desirability can be matched by means of the observed general desirability function $D(Y_i)$. It is defined as the geometric average of the individual desirability functions of each

response d_i (Yi) (Equation 3), where n is the number of responses, instead of directly using the measured (Y_i) and predicted (i) response values.

$$D = \left(\prod_{i=1}^{n} d_i(Y_i) \right)^{\frac{1}{n}}$$

(Equation 3)

The optimal solutions are determined by maximizing the observed general desirability D(Yi) through means of the Levenberg-Marquardt or NelderMead algorithm [39], considering the control limits for each variable (Supplementary Table S1).

3 Results

3.1 Identification of chemical compounds in Guava and Eucalyptus leaf extracts

Guava and Eucalyptus were identified as the plants with the best antibacterial activity in preliminary analyses (data not shown). Therefore, these plant extracts were selected, and their chemical composition were analyzed by HPLC-DAD-MS/MS, being the tentative identification shown in Table 1. The ESI was performed in positive ionization mode and several molecules were detected in both extracts. Regarding Guava extract, the identified molecules correspond to the predominant peaks observed in the obtained chromatogram. The first peak at 13.78 was identified as reynoutrin or avicularin (ID1). The secondary rupture of 303 is associated with quercetin and the loss of 132 uma correspond with a pentoside anglicone. However, it is not possible to differentiate both alternatives with the detected rupture even when the MzCloud provide a higher confidence for reynoutrin (94.4%) than avicularin (88.6%). On the other hand, the 162 loss (ID3) as well as the m/z 303 is consistent with a quercetin glucoside. In the literature, both compounds had been reported in guava leaf extracts [42,43]. The second compound, puerarin (ID2) is an isoflavone and was identified by MzCloud with 92.4% of similarity. Two anthocyanins were identified, more exactly, cyaniding 3-O-pentoside (ID4), which is characterized by the presence of 287 m/z, and petunidin 3-O-pentoside (ID6), which is identified by the primary product ion at 317 m/z [44]. Finally, for the identification of guavinoside compounds (ID5, ID7, and ID10), we based on previous studies [42,45]. Moreover, the secondary rupture of 259 m/z in 181 m/z and 105 m/z was identified in MzCloud as 4-benzoylbiphenyl (85.3). The rupture of compounds with ID8 and ID9 were not identified.

Meanwhile, the identified molecules in Eucalyptus leaf extract also correspond to the predominant peaks observed in the chromatogram (Table 1). Its first peak at 14.45 (ID1) was identified as rutin with a similarity in MzCloud of 88.7%. Moreover, the neutral loss of 308 uma is consistent with a loss of glucose and rhamnose as present in rutin. The ID2 and ID9 were identified as luteolin-7-O-betaglucuronide and resinoside by comparing similar ruptures to those presented in Okhlopkova and colleagues' study [46]. Moreover, resinoside A had been previously described in Eucalyptus [47]. The ID4, ID5 and ID6 compounds were identified as quercetin 3-O-glucuronide, 6-O-methylscutellarin, and glycitin by similarity in MzCloud database with the following scores of 87.9%, 75.8%, and 90.7%, respectively. The ID7 compound was tentatively identified as cypellogin A or B by similar ruptures in

Eucalyptus plant as reported by Boulekbache-Makhlouf and colleagues [48]. The ID8 compound is similar to cypellocarpin C (ID10), where the neutral loss of water (18 uma) produces the product ion at 469 m/z with a further loss of 158 uma probably associated with a consecutive loss of water (leading to 451 m/z), and 140 uma producing the ion at 311 m/z. This rupture could suggest a ring with 3-hydroxyl substitution like in the gallic acid [49]. All these compounds are common in Eucalyptus extracts by previous studies [50,51], as well as cypellocarpin C (ID10) that was identified in MzCloud database with the score of 73.7%. It is important to mention that the reason of the "low" similarity could be due to the consequence of the second rupture. Therefore, the MzCloud peaks were mainly identified through the second rupture derived from the first one. Finally, the chemical compounds labelled as ID11 and ID3 were not identified based on their rupture patterns.

3.2 Quantification of *V. parahaemolyticus* and *V. cholerae* biofilms and their normality assessment

The ability of *V. parahaemolyticus* strain VP 87 and *V. cholerae* strain VC 112 to develop a biofilm was determined by comparing biomass, cell viability, and total cell and live/dead cell counting assays (Figure 1 and Supplementary Tables S2 and S3). An assessment of normality was also applied to the obtained data, using the Anderson-Darling, Ryan-Joiner, and Kolmogorov-Smirnov normality test and the Johnson data transformation method. Subsequently, a reduction of the variable interaction model was performed, eliminating interactions that showed a $p > 0.05$ (Supplementary Table S4). Finally, the same statistical analysis was repeated in the new data set.

As shown in Figure 1 and Supplementary Table S3, *V. parahaemolyticus* biofilms showed a higher ability to produce biomass and culturable sessile cells (CFU counting) during the first 24 h when compared to *V. cholerae* biofilms ($p < 0.001$). However, at 72 h, *V. cholerae* biofilms had a higher ability to produce biomass and culturable sessile cells. Additionally, *V. cholerae* biofilms showed a greater ability to produce biomass and culturable sessile cells at 30 ºC when compared to 24 °C ($p < 0.001$). Normality tests showed normal distribution after the reduction of the model in biomass and viability tests, but a non-normal distribution in the total cell count test, so a data transformation by Johnson's method was performed. Consequently, a parametric statistical analysis was selected for future evaluation.

3.3 Comparison between growth conditions in *Vibrio*-associated biofilms

Biomass and viable cells within the biofilm were quantified demonstrating statistical differences between species at different temperatures, times, and initial inoculums (see Figure 2 and Supplementary Table S4). Multivariate ANOVA analysis showed a significant effect of the variables and the interactions with a p <0.05, to determine the biomass growth and biofilm viability in both *Vibrio* species (see Supplementary Table S4). Both *Vibrio* biofilms had a significant increase in biomass and cell viability at 24 h of growth when compared to 48 h with a p <0.001 in both species by Tukey and Fisher tests, where the biomass and cell viability of *Vibrio*-related biofilms significantly decreased by 43% and 0.5 log (see Figure 2), respectively. Interestingly, *Vibrio*-associated biofilms showed a significant increase in biomass and cell viability at 72 h (see Supplementary Table S4). However, biomass and cell viability had a higher growth of *V. parahaemolyticus* biofilm at 24°C, while *V. cholerae* biofilm showed a higher growth at 30°C, as previously observed through desirability analysis. It is worth mentioning that the initial inoculum was not significant for cell viability (p =0.45), but it was significant for biomass growth (p <0.001) with a slight augmentation of almost 10% at 1E+8 CFU (0.5 MacFarland) in both *Vibrio* species biofilms, therefore it was selected for further biofilm inhibition and eradication assays.

The statistical analysis of biofilm growth by variables comparison method with Tukey and Fisher tests showed a significant difference between *V. parahaemolyticus* and *V. cholerae* biofilms in the biomass assays with crystal violet staining (p <0.001; Supplementary Table S5), but the biomass assays with PBS suspension and cell viability assay did not show any significant differences (p = 0.20 - 0.26), so biomass assays with PBS suspension was chosen for further biofilm inhibition and eradication assays. Likewise, in both *Vibrio* species, Tukey and Fisher tests confirmed statistical differences in all biofilm samples during time in the biomass growth and cell viability assays, in particular between 24 and 48 h (p < 0.001). Contrasting with the previous multivariate ANOVA analysis, Tukey and Fisher tests did not show any significant difference for the initial inoculum variation in the biomass growth and cell viability assays for both species (p = 0.10 - 0.86). Although the temperature in the biomass assays did not evidence significant differences (p = 0.51 - 0.99), Tukey and Fisher tests demonstrated a significant difference for the cell viability assays (p = 0.02). It is interesting to observe that biomass growth trends demonstrating their highest biomass growth and cell viability in the first 24 h at 24°C (Figure 2), where *V. cholerae* reached close to the same level of biomass at 72 h and 30°C (Supplementary Table S5) when compared to the conditions of 24 h at 24 ºC, which contrasted with multivariate ANOVA and desirability analyses.

3.4 Total cell and Live/Dead cells count in *V. parahaemolyticus* and *V. cholerae* biofilms

Next, we analyzed the number of total cells and live/dead cells within biofilms among *Vibrio* species under different growth conditions. As shown in Supplementary Table S5, the fluorescence microscopy (FM) analysis showed a significant effect between samples according to temperature, time, and initial inoculum in both *Vibrio* species through the evaluation of total count, live and dead cells within biofilm samples. When comparing species, statistical differences were observed between *Vibrio* species. More exactly, the dead cells count at 48 h was statistically different by Tukey and Fisher tests (p <0.001), showing a decrease in both species of 43%. Over time *V. parahaemolyticus* showed a higher number of dead cells within the biofilm (around 44%) when compared to *V. cholerae* (p <0.001). Although no statistical differences were found between *Vibrio* species in the total count and live cells, both biofilms evidenced significant differences in their intrinsic growth between 24 and 48 h (p <0.001), as well as 48 and 72 h (p <0.001). Furthermore, concerning temperature variation between 24 and 30 ºC, both *Vibrio*-related biofilm species did not show statistical differences. Interestingly, in FM analysis, the initial inoculum of both *Vibrio* species for biofilm formation showed significant differences for total cell and live/dead cells, where the initial inoculum of 1E+8 CFU (0.5 MacFarland) demonstrated a higher number of live cells and consequently a higher number of dead cells for the initial inoculum of 1E+7 CFU (0.05 MacFarland) was observed (p <0.05). Consequently, it was also observed that *V. parahaemolyticus* and *V. cholerae* demonstrated a higher number of total cells and live cells within the biofilms at 24 h, 24°C, and 1E+8 CFU (0.5 McFarland), as shown in Figure 3.

Although a thoughtful optimization of the FM analysis was performed during the present study, the pictures of live/dead cells within the biofilms of both species did not show the best clarity. However, the merged images showed a greater clarity of the biofilms' development, and the macros Biofilms Viability checker software provided a reliable assessment of the data set. Visual differences in the percentage of dead and alive cells were detected between *Vibrio* species during time, temperature, and initial inoculum. These differences are also validated by multivariate ANOVA analysis and the optimized growth conditions were provided by desirability analysis (Figure 1) through the weighted geometric mean of the individual desirability of the results by PBS at 630 nm, CFU counting and total cell count responses (see Supplementary Table S4). Minitab evaluation determines the optimal settings

for the variables by maximizing the composite desirability, showing a homogeneous distribution of dead cells within the biofilms of both *Vibrio* species (see merged images in Supplementary Figure S1).

3.5 Comparison of the four methodologies to assess biofilm development

After the statistical analysis of the data set, we compared the data accuracy of these different and classical methodologies for *Vibrio*-related biofilm characterization. Therefore, multivariate linear regression ANOVA was applied using CFU counting assays as a reference due to its well-known reliability, and R-squared values were observed as a goodness-of-fit measure for biofilm analysis. As expected, fluorescence microscopy analysis revealed the lowest R-squared values for both *Vibrio*-related biofilms (R^2 = 0.22 - 0.45[illegible]ollowed by biomass assays with CV staining (R^2 = 0.84 - 0.85), and finally biomass assays with PBS suspension (R^2 = 0.86 - 0.92). It is important to mention again that all assays were analyzed at least six assays with five biofilm samples, under the same conditions, to prevent data variability, bias, and manual errors by the researcher during biofilm analysis.

3.6 Antibacterial planktonic activity of Eucalyptus and Guava leaf extracts

As shown in Figure 4, the inhibition of the planktonic growth of *Vibrio parahaemolyticus* (VP87 and tetracycline resistant VP275) and *V. cholerae* strains (VC112) by Eucalyptus and Guava leaf extracts was studied using broth microdilution method during 24 h of incubation at 37 °C and compared to tetracycline and ceftriaxone antibiotics. These assays allow us to determine the lowest inhibitory concentration of the antimicrobial agent (minimal inhibitory concentration, MIC) and to further evaluate other extract concentrations gradually inhibiting bacterial growth through bacterial turbidity. Visible inhibition growth of all *Vibrio* isolates was definitively observed at 1600 µg/mL of Eucalyptus extract and at 200 µg/mL of Guava extract, being these concentrations considered as MIC values for further biofilm inhibition and eradication assays. To better analyze the variables influencing the planktonic growth of *Vibrio* isolates, statistical comparison tests were conducted using the Tukey and Fisher methods between the positive control (medium plus bacterial inoculum) and the different treatment concentrations.

Eucalyptus leaf extract evidenced statistically significant inhibition at a concentration of 50 µg/mL with an average inhibition of 35% against all *Vibrio* isolates ($p < 0.0001$) and reaching its maximum inhibition at 200 µg/mL with an average inhibition of 75% ($p < 0.0001$). Meanwhile, guava leaf extract started exhibiting a more significant inhibition at concentration of 400 µg/mL with an average inhibition of 45% against all *Vibrio* isolates ($p < 0.0001$) demonstrating its maximum inhibition at 1600 µg/mL with an average inhibition of 70% ($p < 0.0001$). On the other hand, antibiotic assays exhibited greater inhibition in the growth of planktonic *Vibrio* isolates as expected due to the purified active compounds, and both antibiotics confirmed their MIC values at 2 µg/mL with an average inhibition of 85% against VP87 and VC112 isolates ($p < 0.0001$). However, as previously reported, VP275 isolate exhibited resistance to all tetracycline concentrations but showing a MIC value of 2 µg/mL by ceftriaxone. These results allowed us to further evaluate the antimicrobial activity through biofilm inhibition and eradication assays with these plant extracts and compared with these standard antibiotics using 1x MIC values as starting point of evaluation.

3.7 Biofilm inhibition and eradication assays

Both biofilm inhibition and eradication assays were performed by the two set of conditions found for the *V. parahaemolyticus* (VP87 and VP275) and *V. cholerae* (VC112) following the desirability analysis for optimal biofilm growth conditions (Figure 1), more exactly, 24 h at 24 °C and 72 h at 30

°C, respectively, before the antibiofilm evaluation by antibiotic and plant extracts. According to the data accuracy analysis of methodologies for *Vibrio*-related biofilm characterization, CFU counting assays and biomass assays with PBS suspension were selected. As shown by Figure 5, both antibiotics and plant extracts were able to inhibit biofilm biomass development starting by 1x MIC values during 24 h at 24 °C, excepting tetracycline against *V. parahaemolyticus* VP275 due to its intrinsic antimicrobial resistance but still showing statistical inhibition when compared to control ($p < 0.0001$). Meanwhile, Guava extract showed its higher biomass inhibition (87%) at the concentration of 12800 µg/mL (8x MIC value) and 90% of inhibition in viable cells at the concentration of 25600 µg/mL (16x MIC value) against all *Vibrio* sp. biofilms ($p < 0.0001$; see Supplementary Table S6). However, differences were observed in the CFU counting between antibiotics and plant extracts, where both extract plants demonstrated higher reduction of viable cells when compared with both antibiotics at 8x, 16x, and 32x MIC values (Eucalyptus extract: 1600, 3200, and 6400 µg/mL; while Guava extract: 12800, 25600, and 52000 µg/mL). Concerning the biofilm growth conditions of 72 h at 30 °C, different results were obtained as shown in Figure 6, where the inhibition of biofilm biomass formation was less notorious by Guava leaf extract and tetracycline although still maintaining statistical differences when compared to control ($p < 0.0001$). When looking to the biomass formation, ceftriaxone apparently evidenced a higher *Vibrio*-related biofilm inhibition when compared to the Eucalyptus extract at all MIC values. However, CFU counting assays demonstrated that, despite a higher biomass presence within the biofilm samples, Eucalyptus extract notoriously reduced the total number of viable cells within *Vibrio*-biofilm samples since 2x until 32x MIC values (400-6400 µg/mL) when compared to the same MIC values of ceftriaxone (5-80 µg/mL), which was not able to reduce the number of viable cells after 1x MIC value (2 µg/mL). Moreover, Eucalyptus extract demonstrated similar results with no significant differences in terms of biomass and CFU reduction in biofilm inhibition assays at both sets of growth conditions (24 h at 24°C and 72 h at 30°C), showing an average inhibition of approximately 80% at concentration of 400 µg/mL for all *Vibrio* isolates ($p < 0.0001$; see Supplementary Table S7).

Further antibiofilm evaluation was studied through eradication biofilm assays and remarkably the same trend of results was observed, i.e., significant eradication values were obtained against all *Vibrio* species or strains starting by 1x MIC values at both sets of growth conditions (24 h at 24°C and 72 h at 30°C) when compared to control ($p < 0.0001$), as shown in Figures 7 and 8. However, as expected, biofilm eradication values were substantially lower by all antibiotics and plant extracts when compared to biofilm inhibition assays. Once again, differences were detected in the CFU counting between antibiotics and plant extracts, where both extract plants demonstrated higher reduction of viable cells when compared with both antibiotics at 8x, 16x, and 32x MIC values at both sets of growth conditions except for *V. cholerae* (VC112) biofilms of 72 h at 30 °C (Figure 8), which demonstrated resistance against Guava extract in both biomass and cell viability evaluation thus confirming its optimal growth conditions previously assessed. Nonetheless, Guava extract demonstrated excellent eradication values in biofilms of 24 h at 24°C reducing 60% of biomass and 90% of viable cells at the concentration of 25600 µg/mL for all *Vibrio* isolates ($p < 0.0001$ see Supplementary Table S7, while 72 h and 30°C exhibit its greater effectiveness with an average inhibition of 72% in biomass and viable cells at the concentration of 51200 µg/mL against *Vibrio* isolates ($p < 0.0001$; see Supplementary Table S7. Meanwhile, Eucalyptus extract at 800 µg/mL reduced approximately 70% of biomass and 90% of viable cells for all *Vibrio* isolates ($p < 0.0001$). It is important to mention that no significant difference was observed in the eradication ability of Eucalyptus extract at both sets of growth conditions apart from the biomass eradication of *V. cholerae* (VC112) biofilms of 72 h at 30 °C (Figure 8). Therefore,

the overall results of the present study demonstrated the higher effectiveness of Eucalyptus extract to inhibit and eradicate *Vibrio*-related biofilms independently of time and temperature conditions.

4 Discussion

The *Vibrio* genus possesses several pathogens that directly affect the aquaculture industry, according to the Food and Agriculture Organization (FAO), such as the shrimp industry [2]. Nowadays, the shrimp industry is experiencing the highest growth rate with production increasing by approximately 9.4% annually [1]. However, chemical products have been used for decades to treat *Vibrio*-associated infections, leading to an increase in antimicrobial resistance [52]. These *Vibrio*-associated infections result in economic losses of up to 3 billion US dollars in global production, affecting all countries that produce crustaceans [53,54]. In Ecuador, the shrimp industry represents 19% of its exportation market and 5 billion US dollars. According to the Global Outlook for Aquaculture Leadership (GOAL) in 2019, Ecuador is the leading shrimp producer and exporter in the world [3]. As a result, shrimp production losses have a significant impact on the Ecuadorian economy. Therefore, efforts have been made to find an eco-friendly solution using plant extracts to counteract the problems of resistance and environmental impact associated with antibiotic use, which is prohibited in Ecuador and other countries, or other chemical compounds.

In the present study, we first characterized the main variables (temperature, time, and initial inoculum) influencing the development of biofilms by *Vibrio parahaemolyticus* and *V. cholerae*. The initial inoculum was not significant for cell viability, but it was significant for biomass growth with a slight augmentation at 1E+8 CFU in both *Vibrio* species biofilms, as expected from studies with different biofilm-forming microorganisms [21,55,56]. However, our results evidenced that optimized *V. parahaemolyticus* biofilms were obtained at 24ºC during 24 h while optimized *V. cholerae* biofilms were observed at 30 ºC during 72h. This characterization agreed with previous studies [17,57], confirming that certain growth variables could influence the *Vibrio* species' dominance in the aquatic environment and *Vibrio*-associated biofilm infections. Furthermore, several authors already reported the great diversity of *Vibrio* species in seafood, including shrimp, but also in different geographical areas [58–60]. In fact, a systematic review and meta-analysis demonstrated that *V. parahaemolyticus* could only be isolated around 47.5 % of the 5811 seafood samples analyzed [14], evidencing the heterogeneity and prevalence of *Vibrio* species among seafood. Therefore, the present work also showed the importance of expanding surveillance on different *Vibrio* species among seafood types and industrial aquaculture areas to identify critical control points (such as temperature and time) for the seafood production system and avoid public health impact.

Next, the planktonic antibacterial activity of Eucalyptus and Guava extracts was evaluated against *V. parahaemolyticus* and *V. cholerae* strains and compared to antibiotic treatments. Our results revealed that Eucalyptus extract exhibited a significant planktonic growth inhibition on these *Vibrio* species at concentrations ranging from (50 to 200 µg/mL), being similar values to those reported by Sivaraj and colleagues [61] for *V. parahaemolyticus* (31.25 to 125 µg/mL), as well as Nwabor and colleagues [62] for *Listeria monocytogenes* (64 to 128 µg/mL). On the other hand, the Guava extract displayed a planktonic growth inhibition in the range of 400 to 1600 µg/mL in this study, which are lower values than the values reported by Rahim et al. [63] and Hoque et al. [64], more exactly concentrations between 1250 and 3000 µg/mL for *V. cholerae* and *V. parahaemolyticus*. In comparison, tetracycline and ceftriaxone exhibited higher inhibition at 2 µg/mL, as purified compounds, in sensitive strains (VP87 and VC112) but were unable to inhibit resistant *Vibrio* strains such as tetracycline-resistant VP275.

Further evaluation was performed through biofilm inhibition and eradication assays using biomass and colony-forming unit (CFU) counting methodologies. To the authors' best knowledge, the present study is the first study to report the biofilm inhibition and eradication assays against *Vibrio* species with both methodologies, allowing thus to characterize biofilm reduction and cell viability within biofilms. Given the absence of literature about cell viability assays, our results were compared with those studies evaluating biomass reduction. However, it is important to note that plant extracts revealed equal (Guava extract) or greater (Eucalyptus extract) biofilm inhibition and eradication values by CFU counting evaluation when compared to tetracycline and ceftriaxone, evidencing greater antibiofilm activities than the initial biomass evaluation and also suggesting synergy between multiple compounds in both plant extracts. Regarding biomass evaluation, Eucalyptus extract achieved a 90% of biofilm inhibition at 400 µg/mL (2x MIC value). Although no studies were found regarding biofilm inhibition and eradication in *Vibrio* species, several studies reported biofilm inhibition levels up to 95.9% with Eucalyptus extract ranging from 250 to 393 µg/mL against other bacterial species such as *L. monocytogenes*, *Escherichia coli*, *Staphylococcus aureus*, and *Streptococcus pyogenes* [62,65,66]. Concerning biofilm eradication assays, Eucalyptus extract at 800 µg/mL (4x MIC value) was able to eradicate more than 70% of all *Vibrio* biofilms in agreement with Tsukatani and colleagues where Eucalyptus extract eradicated 99% of *Porphyromonas gingivalis* JCM12257, *S. aureus* NBRC13276, and *Streptococcus mutans* NBRC13955 biofilms showing minimum biofilm eradication concentration (MBEC) values of 49.1 µg/mL for *P. gingivalis* and 393 µg/mL for *S. aureus* and *S. mutans* [67]. However, in the same study, Eucalyptus extract at superior concentrations of 1572 µg/mL was only able to eradicate 21.0% of *E. coli* NBRC15034 biofilms [67]. Meanwhile, in the present study, the Guava extract showed a higher biomass inhibition at concentrations of 12800 µg/mL (8x MIC value), evidencing an average of 80% biomass inhibition among *Vibrio* isolates except for VC112 at 30 ºC. Further evaluation demonstrated the Guava extract's ability at 25600 µg/mL (16x MIC value) to eradicate an average of 70% of the biomass in all *Vibrio* sp. excepting once again VC112 at 30 ºC. In terms of eradication, other studies only evaluated Guava extract against different pathogenic species, such as *S. aureus* [68,69]. These studies reported eradication levels of up to 44% and 59% in *S. aureus* biofilms when applying Guava extracts from 440 to 870 µg/mL and 1100 to 2100 µg/mL [68,69], respectively. These lower eradication values when compared to the present study could be due to the lower applied concentrations of Guava extract as well as the nature of *S. aureus* biofilms virulence mechanisms. Despite the use of different measurement methods, our MIC values agree with the values reported by Sivaraj et al. and Nwabor et al. showing MIC values for Eucalyptus extract of 31.25 to 125 µg/mL and 64 to 128 µg/mL [61,62], respectively. Likewise, Rahim et al. and Hoque et al. also reported similar MIC values for Guava extract stating concentration ranges of 400 to 1600 µg/mL and 1250 to 3000 µg/mL [63,64], respectively. However, little is still known about the potential of plant extracts against *Vibrio* species. In fact, there is a dearth of literature related to the cellular viability results (CFU/mL) in biofilm inhibition and eradication assays. This becomes a challenge when comparing with the existing literature and demonstrates the importance of the present study, where the reduction in the cellular viability of *Vibrio* biofilms is equal to or even greater than tetracycline and ceftriaxone antibiotics even though the extracts are diluted in an aqueous solvent and their compounds were not purified. Future studies should evaluate the antibiofilm activity of the identified compounds of the present study.

Moreover, Guava and Eucalyptus extracts contain a variety of compounds already reported in the literature [70–72], including flavonoids, isoflavonoids, and anthocyanins. In the present study, we found cypellogin A or B and cypellocarpin C in Eucalyptus extract in agreement the literature [73]. Likewise, our identification of guavinoside C, guavinoside A isomer, and guavinoside B in Guava

extract is consistent with Rojas-Garbanzo and colleagues' study [74]. Regarding these molecules, few specific antimicrobial assays using purified compounds were found, reporting mainly antimicrobial activities by guavinoside-like compounds [75]. On the other hand, other identified molecules in the present study were widely found in other plants and already demonstrated antimicrobial properties in previous studies [76,77]. More exactly, puerarin, quercetin, quercetin glucoside, and rutin showed specific antimicrobial activity against *Vibrio* species including *V. cholerae* [76,78–80]. The antimicrobial activity could be attributed to various mechanisms and the presence of multiple compounds in these plant extracts could lead to synergistic effects that enhance their antibiofilm effects. The mechanisms involving flavonoids have been discussed by other authors [27,81–83]. In general, flavonoids are able to suppress nucleic acid synthesis and affect the cytoplasmic membrane as well as other possible effects. Considering the identified flavonoids in the present study, quercetin, rutin, and luteolin can induce a membrane disruption and even affect biofilm formation, while quercetin and similar flavonoids can actually disrupt the bacterial fatty acid metabolism [27,82]. Similarly, anthocyanins (like cyanidin derivates found in Guava) can also affect biofilm formation probably by their chelating properties and interfere with the bacterial ATP synthesis [82,84]. For many other flavonoids or polyphenols, no specific data is known regarding effects or mechanisms, but it is safe to assume that will share similar effects as observed in other flavonoids. The combination of these effects could weaken bacteria and lead to their lysis and death. Moreover, considering the low resistance of bacteria to flavonoids [27,85,86] as well as the increment in antibiotic effects when combined with flavonoids [83,87], it is important to explore further these and others natural product extracts to reduce the industrial use of conventional antibiotics or other chemical compounds with great impact on the environment. Therefore, it is important to carry out other studies to fully understand the precise mechanisms behind the antimicrobial activity of these extracts and their ability to eliminate *Vibrio*-associated biofilms.

Another shortcoming of the present study was the lack of *in vitro* tests for toxicological evaluation on hemolytic shrimp cells and *in vivo* assessments within the shrimp industry in Ecuador to verify the effectiveness and define optimal treatment ranges against *Vibrio*-associated biofilms to eliminate their continuous persistence in aquaculture. Therefore, future research should explore the practical applications of these identified compounds, more exactly cypellogin A, cypellogin B, and cypellocarpin C from Eucalyptus extract, as well as guavinoside A, B, and C compounds from Guava extract. Several studies tried to develop alternative treatments to fight vibriosis and antibiotic resistance [88]. However, there is still a need to focus on their practical application. The use of natural compounds from plant extracts is one of the most promising alternatives for the treatment and prevention of diseases in aquaculture. These compounds are eco-friendly allowing them to support the economy and reducing food spoilage. Overall, our results from aqueous extracts of Guava and Eucalyptus plants suggested a viable alternative in the ongoing quest for new alternative treatments against vibriosis in the shrimp industry serving as natural antimicrobial agents, cost-effective, sustainable, and environmentally friendly approach in Ecuador.

5 Conflict of Interest

The authors declare that the research was conducted in the absence of any commercial or financial relationships that could be construed as a potential conflict of interest.

6 Author Contributions

Experimental research: N.R.J.-M., D.F.C., A.C.C.-P., A.G., and D.A.-P. Methodology: D.A.-P., M.A.-M., L.O.-E., E.T., and A.M. Validation: D.A.-P., M.A.-M., L.O.-E., E.T., and A.M. Formal Analysis:

N.R.J.-M., D.F.C., A.C.C.-P., A.G., D.A.-P., M.A.-M., L.O.-E., E.T., and A.M. Resources: D.A.-P., M.A.-M., L.O.-E., E.T., and A.M. Data curation: D.A.-P., M.A.-M., L.O.-E., E.T., and A.M. Writing-Original Draft Preparation: N.R.J.-M., A.C.C.-P., A.G., L.O.-E., E.T., and A.M. Writing-Review, and Editing: L.O.-E., E.T., and A.M. Supervision: A.M. Project Administration and Funding: A.M. with USFQ Chancellor Grants.

7 Funding

This work is supported by the COCIBA research budget to António Machado from Universidad San Francisco de Quito, under the Project ID: 17357 entitled "Alternative approaches for eliminating Biofilms" and the Project ID: 16801 entitled ''Characterization of single and mixed Biofilms''. The funders had no role in the study design, data collection, analysis, decision to publish, or preparation of the manuscript. The APC was funded by the Research Office of Universidad San Francisco de Quito (USFQ).

8 Acknowledgments

Special recognition deserves all colleagues of the Microbiology Institute of Universidad San Francisco de Quito (IM-USFQ), COCIBA, and Research Office of USFQ for their support in this study. We are grateful to David Valencia and Darío Cueva for their assistance in fluorescence microscopy assays.

9 References

1. Arunkumar M, LewisOscar F, Thajuddin N, Pugazhendhi A, Nithya C. In vitro and in vivo biofilm forming Vibrio spp: A significant threat in aquaculture. Process Biochemistry. 2020;94: 213–223. doi:10.1016/j.procbio.2020.04.029

2. Sheikh H, John A, Musa N, abdulrazzak LA, Alfatama M, Fadhlina A. Vibrio spp. and Their Vibriocin as a Vibriosis Control Measure in Aquaculture. Appl Biochem Biotechnol. 2022;194: 4477–4491. doi:10.1007/s12010-022-03919-3

3. Anderson BJL, Valderrama D, Jory DE. GOAL 2019: Revisión de la producción mundial de camarones. Global Aquaculture Advocate. 2019; 7–11.

4. ISSUU. The Ecuadorian shrimp industry and its market opportunities - Issuu. 2021.

5. Sanches-Fernandes GMM, Sá-Correia I, Costa R. Vibriosis Outbreaks in Aquaculture: Addressing Environmental and Public Health Concerns and Preventive Therapies Using Gilthead Seabream Farming as a Model System. Front Microbiol. 2022;13. doi:10.3389/fmicb.2022.904815

6. Machado A, Zamora-Mendoza L, Alexis F, Álvarez-Suarez JM. Use of Plant Extracts, Bee-Derived Products, and Probiotic-Related Applications to Fight Multidrug-Resistant Pathogens in the Post-Antibiotic Era. Future Pharmacology. 2023;3: 535–567. doi:10.3390/futurepharmacol3030034

7. Kewcharoen W, Srisapoome P. Probiotic effects of Bacillus spp. from Pacific white shrimp (Litopenaeus vannamei) on water quality and shrimp growth, immune responses, and resistance

to Vibrio parahaemolyticus (AHPND strains). Fish Shellfish Immunol. 2019;94: 175–189. doi:10.1016/j.fsi.2019.09.013

8. Ganesh KN, Zhang D, Miller SJ, Rossen K, Chirik PJ, Kozlowski MC, et al. Green Chemistry: A Framework for a Sustainable Future. ACS Omega. 2021;6: 16254–16258. doi:10.1021/acsomega.1c03011

9. Salvatori ES, Morgan L V., Ferrarini S, Zilli GAL, Rosina A, Almeida MOP, et al. Anti-Inflammatory and Antimicrobial Effects of Eucalyptus spp. Essential Oils: A Potential Valuable Use for an Industry Byproduct. Evidence-Based Complementary and Alternative Medicine. 2023;2023: 1–13. doi:10.1155/2023/2582698

10. Díaz-de-Cerio E, Verardo V, Gómez-Caravaca A, Fernández-Gutiérrez A, Segura-Carretero A. Health Effects of Psidium guajava L. Leaves: An Overview of the Last Decade. Int J Mol Sci. 2017;18: 897. doi:10.3390/ijms18040897

11. Roy PK, Mizan MFR, Hossain MI, Han N, Nahar S, Ashrafudoulla M, et al. Elimination of Vibrio parahaemolyticus biofilms on crab and shrimp surfaces using ultraviolet C irradiation coupled with sodium hypochlorite and slightly acidic electrolyzed water. Food Control. 2021;128: 108179. doi:10.1016/j.foodcont.2021.108179

12. Wang D, Flint SH, Palmer JS, Gagic D, Fletcher GC, On SLW. Global expansion of Vibrio parahaemolyticus threatens the seafood industry: Perspective on controlling its biofilm formation. LWT. Academic Press; 2022. doi:10.1016/j.lwt.2022.113182

13. Teschler JK, Zamorano-Sánchez D, Utada AS, Warner CJA, Wong GCL, Linington RG, et al. Living in the matrix: Assembly and control of Vibrio cholerae biofilms. Nature Reviews Microbiology. Nature Publishing Group; 2015. pp. 255–268. doi:10.1038/nrmicro3433

14. Odeyemi OA. Incidence and prevalence of Vibrio parahaemolyticus in seafood: a systematic review and meta-analysis. Springerplus. 2016;5. doi:10.1186/s40064-016-2115-7

15. Vestby LK, Grønseth T, Simm R, Nesse LL. Bacterial biofilm and its role in the pathogenesis of disease. Antibiotics. 2020;9. doi:10.3390/ANTIBIOTICS9020059

16. Flemming H-C, van Hullebusch ED, Neu TR, Nielsen PH, Seviour T, Stoodley P, et al. The biofilm matrix: multitasking in a shared space. Nature Reviews Microbiology 2022. 2022; 1–17. doi:10.1038/s41579-022-00791-0

17. Loni Townsley, Yildiz nd FH. Temperature affects c-di-GMP signaling and biofilm formation in Vibrio cholerae. Physiol Behav. 2018;176: 139–148. doi:10.1111/1462-2920.12799.Temperature

18. Abe K, Nomura N, Suzuki S. Biofilms: hot spots of horizontal gene transfer (HGT) in aquatic environments, with a focus on a new HGT mechanism. FEMS Microbiol Ecol. 2020;96: fiaa031. doi:10.1093/femsec/fiaa031

19. Sajali USBA, Atkinson NL, Desbois AP, Little DC, Murray FJ, Shinn AP. Prophylactic properties of biofloc- or Nile tilapia-conditioned water against Vibrio parahaemolyticus

infection of whiteleg shrimp (Penaeus vannamei). Aquaculture. 2019;498: 496–502. doi:10.1016/j.aquaculture.2018.09.002

20. INEN. Camarones o Langostinos congelados. 2013; 3–4.

21. Atiencia-Carrera MB, Cabezas-Mera FS, Vizuete K, Debut A, Tejera E, Machado A. Evaluation of the biofilm life cycle between Candida albicans and Candida tropicalis. Front Cell Infect Microbiol. 2022;12: 953168. doi:10.3389/fcimb.2022.953168

22. Soll DR, Daniels KJ. Plasticity of Candida albicans Biofilms. Microbiology and Molecular Biology Reviews. 2016;80: 565–595. doi:10.1128/mmbr.00068-15

23. Beltrán-Noboa A, Proaño-Ojeda J, Guevara M, Gallo B, Berrueta LA, Giampieri F, et al. Metabolomic profile and computational analysis for the identification of the potential anti-inflammatory mechanisms of action of the traditional medicinal plants Ocimum basilicum and Ocimum tenuiflorum. Food and Chemical Toxicology. 2022;164: 113039. doi:10.1016/j.fct.2022.113039

24. Lohse MB, Gulati M, Johnson AD, Nobile CJ. Development and regulation of single-and multi-species Candida albicans biofilms. Nat Rev Microbiol. 2018;16: 19–31. doi:10.1038/NRMICRO.2017.107

25. Rosenberg M, Azevedo NF, Ivask A. Propidium iodide staining underestimates viability of adherent bacterial cells. Sci Rep. 2019;9: 1–12. doi:10.1038/s41598-019-42906-3

26. Mountcastle SE, Vyas N, Villapun VM, Cox SC, Jabbari S, Sammons RL, et al. Biofilm viability checker: An open-source tool for automated biofilm viability analysis from confocal microscopy images. NPJ Biofilms Microbiomes. 2021;7: 1–12. doi:10.1038/s41522-021-00214-7

27. Cabezas-Mera FS, Atiencia-Carrera MB, Villacrés-Granda I, Proaño AA, Debut A, Vizuete K, et al. Evaluation of the polyphenolic profile of native Ecuadorian stingless bee honeys (Tribe: Meliponini) and their antibiofilm activity on susceptible and multidrug-resistant pathogens: An exploratory analysis. Curr Res Food Sci. 2023;7: 100543. doi:10.1016/j.crfs.2023.100543

28. Castro J, Lima Â, Sousa LGV, Rosca AS, Muzny CA, Cerca N. Crystal Violet Staining Alone Is Not Adequate to Assess Synergism or Antagonism in Multi-Species Biofilms of Bacteria Associated With Bacterial Vaginosis. Front Cell Infect Microbiol. 2022;11: 1375. doi:10.3389/FCIMB.2021.795797/BIBTEX

29. Rodríguez-Arias RJ, Guachi-Álvarez BO, Montalvo-Vivero DE, Machado A. Lactobacilli displacement and Candida albicans inhibition on initial adhesion assays: a probiotic analysis. BMC Res Notes. 2022;15: 1–7. doi:10.1186/s13104-022-06114-z

30. Schindelin J, Arganda-carreras I, Frise E, Kaynig V, Longair M, Pietzsch T, et al. Fiji : an open-source platform for biological-image analysis. Nat Methods. 2012;9: 676–682. doi:10.1038/nmeth.2019

31. Mountcastle SE, Vyas N, Villapun VM, Cox SC, Jabbari S, Sammons RL, et al. Biofilm viability checker: An open-source tool for automated biofilm viability analysis from confocal microscopy images. NPJ Biofilms Microbiomes. 2021;7: 1–12. doi:10.1038/s41522-021-00214-7

32. Legland D, Arganda-Carreras I, Andrey P. MorphoLibJ: integrated library and plugins for mathematical morphology with ImageJ. Bioinformatics. 2016;32: 3532–3534. doi:10.1093/bioinformatics/btw413

33. Mcquin C, Goodman A, Chernyshev V, Kamentsky L, Cimini A, Karhohs KW, et al. CellProfiler 3.0: Next-generation image processing for biology. PLoS Biol. 2018;16: e2005970. doi:10.1371/journal.pbio.2005970

34. Wiegand I, Hilpert K, Hancock REW. Agar and broth dilution methods to determine the minimal inhibitory concentration (MIC) of antimicrobial substances. Nat Protoc. 2008;3: 163–175. doi:10.1038/nprot.2007.521

35. Patel B, Siddiqui A, Hamadou W, Awadelkareem A, Ashraf S, Alreshidi M, et al. Inhibition of Bacterial Adhesion and Antibiofilm Activities of a Glycolipid Biosurfactant from Lactobacillus rhamnosus with Its Physicochemical and Functional Properties. Antibiotics. 2021;10: 25.

36. Fernandez-Soto P, Celi D, Tejera E, Alvarez-Suarez JM, Machado A. Cinnamomum sp. and Pelargonium odoratissimum as the Main Contributors to the Antibacterial Activity of the Medicinal Drink Horchata: A Study Based on the Antibacterial and Chemical Analysis of 21 Plants. Molecules. 2023;28. doi:10.3390/molecules28020693

37. Sornsenee P, Chatatikun M, Mitsuwan W, Kongpol K, Kooltheat N, Sohbenalee S, et al. Lyophilized cell-free supernatants of Lactobacillus isolates exhibited antibiofilm, antioxidant, and reduces nitric oxide activity in lipopolysaccharide- stimulated RAW 264.7 cells. PeerJ. 2021;9. doi:10.7717/peerj.12586

38. Minitab Statical Software. Minitab Version 20. 2022.

39. Machado A, Tejera E, Cruz-Monteagudo M, Rebelo I. Application of desirability-based multi(bi)-objective optimization in the design of selective arylpiperazine derivates for the 5-HT1A serotonin receptor. Eur J Med Chem. 2009;44: 5045–5054. doi:10.1016/j.ejmech.2009.09.008

40. Tejera E, Machado A, Rebelo I, Nieto-Villar J. Fractal protein structure revisited: Topological, kinetic and thermodynamic relationships. Physica A: Statistical Mechanics and its Applications. 2009;388: 4600–4608. doi:10.1016/j.physa.2009.07.015

41. Amdoun R, Khelifi L, Khelifi-Slaoui M, Amroune S, Asch M, Assaf-ducrocq C, et al. The Desirability Optimization Methodology; a Tool to Predict Two Antagonist Responses in Biotechnological Systems: Case of Biomass Growth and Hyoscyamine Content in Elicited Datura starmonium Hairy Roots. Iran J Biotechnol. 2018;16: 11–19. doi:10.21859/ijb.1339

42. Matsuzaki K, Ishii R, Kobiyama K, Kitanaka S. New benzophenone and quercetin galloyl glycosides from Psidium guajava L. J Nat Med. 2010;64: 252–256. doi:10.1007/s11418-010-0400-2

43. Kumar M, Tomar M, Amarowicz R, Saurabh V, Nair MS, Maheshwari C, et al. Guava (Psidium guajava L.) Leaves: Nutritional Composition, Phytochemical Profile, and Health-Promoting Bioactivities. Foods. 2021;10: 752. doi:10.3390/foods10040752

44. Yoshimura Y, Zaima N, Moriyama T, Kawamura Y. Different Localization Patterns of Anthocyanin Species in the Pericarp of Black Rice Revealed by Imaging Mass Spectrometry. PLoS One. 2012;7: e31285. doi:10.1371/journal.pone.0031285

45. Díaz-de-Cerio E, Gómez-Caravaca AM, Verardo V, Fernández-Gutiérrez A, Segura-Carretero A. Determination of guava (Psidium guajava L.) leaf phenolic compounds using HPLC-DAD-QTOF-MS. J Funct Foods. 2016;22: 376–388. doi:10.1016/j.jff.2016.01.040

46. Okhlopkova ZM, Razgonova MP, Pikula KS, Zakharenko AM, Piekoszewski W, Manakov YA, et al. Dracocephalum palmatum S. and Dracocephalum ruyschiana L. Originating from Yakutia: A High-Resolution Mass Spectrometric Approach for the Comprehensive Characterization of Phenolic Compounds. Applied Sciences. 2022;12: 1766. doi:10.3390/app12031766

47. Heskes AM, Goodger JQD, Tsegay S, Quach T, Williams SJ, Woodrow IE. Localization of Oleuropeyl Glucose Esters and a Flavanone to Secretory Cavities of Myrtaceae. PLoS One. 2012;7: e40856. doi:10.1371/journal.pone.0040856

48. Boulekbache-Makhlouf L, Meudec E, Mazauric J-P, Madani K, Cheynier V. Qualitative and Semi-quantitative Analysis of Phenolics in *Eucalyptus globulus* Leaves by High-performance Liquid Chromatography Coupled with Diode Array Detection and Electrospray Ionisation Mass Spectrometry. Phytochemical Analysis. 2013;24: 162–170. doi:10.1002/pca.2396

49. Hamed AI, Al-Ayed AS, Moldoch J, Piacente S, Oleszek W, Stochmal A. Profiles analysis of proanthocyanidins in the argun nut (*Medemia argun* -an ancient Egyptian palm) by LC-ESI-MS/MS. Journal of Mass Spectrometry. 2014;49: 306–315. doi:10.1002/jms.3344

50. Ito H, Koreishi M, Tokuda H, Nishino H, Yoshida T. Cypellocarpins A−C, Phenol Glycosides Esterified with Oleuropeic Acid, from *Eucalyptus cypellocarpa*. J Nat Prod. 2000;63: 1253–1257. doi:10.1021/np0001981

51. Tian L-W, Zhang Y-J, Wang Y-F, Lai C-C, Yang C-R. Eucalmaidins A−E, (+)-Oleuropeic Acid Derivatives from the Fresh Leaves of *Eucalyptus maideni*. J Nat Prod. 2009;72: 1608–1611. doi:10.1021/np900290s

52. Ghosh AK, Panda SK, Luyten W. Anti-vibrio and immune-enhancing activity of medicinal plants in shrimp: A comprehensive review. Fish Shellfish Immunol. 2021;117: 192–210. doi:10.1016/J.FSI.2021.08.006

53. Istiqomah I, Sukardi, Murwantoko, Isnansetyo A. Review Vibriosis Management in Indonesian Marine Fish Farming. E3S Web of Conferences. 2020;147. doi:10.1051/E3SCONF/202014701001

54. de Souza Valente C, Wan AHL. Vibrio and major commercially important vibriosis diseases in decapod crustaceans. J Invertebr Pathol. 2021;181. doi:10.1016/j.jip.2020.107527

55. Cangui-Panchi SP, Ñacato-Toapanta AL, Enríquez-Martínez LJ, Reyes J, Garzon-Chavez D, Machado A. Biofilm-forming microorganisms causing hospital-acquired infections from intravenous catheter: A systematic review. Curr Res Microb Sci. 2022;3: 100175. doi:10.1016/j.crmicr.2022.100175

56. Cangui-Panchi SP, Ñacato-Toapanta AL, Enríquez-Martínez LJ, Salinas-Delgado GA, Reyes J, Garzon-Chavez D, et al. Battle royale: Immune response on biofilms – host-pathogen interactions. Current Research in Immunology. 2023;4. doi:10.1016/j.crimmu.2023.100057

57. Yildiz FH, Visick KL. Vibrio biofilms: so much the same yet so different. Trends Microbiol. 2009;17: 109–118. doi:10.1016/j.tim.2008.12.004

58. Janecko N, Bloomfield SJ, Palau R, Mather AE. Whole genome sequencing reveals great diversity of Vibrio spp in prawns at retail. Microb Genom. 2021;7. doi:10.1099/mgen.0.000647

59. Abdalla T, Al-Rumaithi H, Osaili TM, Hasan F, Obaid RS, Abushelaibi A, et al. Prevalence, Antibiotic-Resistance, and Growth Profile of Vibrio spp. Isolated From Fish and Shellfish in Subtropical-Arid Area. Front Microbiol. 2022;13. doi:10.3389/fmicb.2022.861547

60. Elbashir S, Jahncke M, DePaola A, Bowers J, Schwarz J, Punchihewage-Don AJ, et al. Prevalence and Abundance of Bacterial Pathogens of Concern in Shrimp, Catfish and Tilapia Obtained at Retail Stores in Maryland, USA. Pathogens. 2023;12: 187. doi:10.3390/pathogens12020187

61. Sivaraj A, Devagnanaroopan J, Palani B, Senthilkumar B. Antibacterial activity of Eucalyptus oblique aqueous leaf extract on selective Vibrio species of Penaeus monodon culture hatchery. World Journal of Pharmaceutical Sciences. 2017. Available: http://www.wjpsonline.org/

62. Nwabor OF, Vongkamjan K, Voravuthikunchai SP. Antioxidant Properties and Antibacterial Effects of Eucalyptus camaldulensis Ethanolic Leaf Extract on Biofilm Formation, Motility, Hemolysin Production, and Cell Membrane of the Foodborne Pathogen Listeria monocytogenes. Foodborne Pathog Dis. 2019;16: 581–589. doi:10.1089/fpd.2019.2620

63. Rahim N, Gomes DJ, Watanabe H, Rahman SR, Chomvarin C, Endtz HP, et al. Antibacterial Activity of Psidium guajava Leaf and Bark against Multidrug-Resistant Vibrio cholerae: Implication for Cholera Control. Jpn J Infect Dis. 2010.

64. Hoque MDM, Bari ML, Inatsu Y, Juneja VK, Kawamoto S. Antibacterial Activity of Guava (Psidium guajava L.) and Neem (Azadirachta indica A. Juss.) Extracts Against Foodborne Pathogens and Spoilage Bacteria. 2007.

65. Okba MM, El-Shiekh RA, Abu-Elghait M, Sobeh M, Ashour RMS. Hplc-pda-esi-ms/ms profiling and anti-biofilm potential of eucalyptus sideroxylon flowers. Antibiotics. 2021;10. doi:10.3390/antibiotics10070761

66. Vijayakumar K, Manigandan V, Jeyapragash D, Bharathidasan V, Anandharaj B, Sathya M. Eucalyptol inhibits biofilm formation of Streptococcus pyogenes and its mediated virulence factors. J Med Microbiol. 2020;69: 1308–1318. doi:10.1099/jmm.0.001253

67. Tsukatani T, Sakata F, Kuroda R, Akao T. Biofilm Eradication Activity of Herb and Spice Extracts Alone and in Combination Against Oral and Food-Borne Pathogenic Bacteria. Curr Microbiol. 2020;77: 2486–2495. doi:10.1007/s00284-020-02017-z

68. Dutta K, Karmakar A, Jana D, Ballav S, Shityakov S, Panda AK, et al. Benzyl isocyanate isolated from the leaves of Psidium guajava inhibits Staphylococcus aureus biofilm formation. Biofouling. 2020;36: 1000–1017. doi:10.1080/08927014.2020.1842877

69. dos Santos RM, Costa G, Cerávolo IP, Dias-Souza MV. Antibiofilm potential of Psidium guajava and Passiflora edulis pulp extracts against Staphylococcus aureus, cytotoxicity, and interference on the activity of antimicrobial drugs. Futur J Pharm Sci. 2020;6. doi:10.1186/s43094-020-00056-8

70. Shahidi F, Varatharajan V, Oh WY, Peng H. Phenolic compounds in agri-food by-products, their bioavailability and health effects. Journal of Food Bioactives. 2019;5. doi:10.31665/jfb.2019.5178

71. Kim YS, Hwang CS, Shin DH. Volatile constituents from the leaves of Polygonum cuspidatum S. et Z. and their anti-bacterial activities. Food Microbiol. 2005;22: 139–144. doi:10.1016/j.fm.2004.01.016

72. Badria FA, Abu Habib MM, Abdel Bar FM, Badria Antioxidant FA. compounds from the leaves of Psidium guajava. World J Pharm Sci. 2017;5: 56–63. Available: http://www.wjpsonline.org/

73. Ito H, Koreishi M, Tokuda H, Nishino H, Yoshida T. Cypellocarpins A−C, Phenol Glycosides Esterified with Oleuropeic Acid, from *Eucalyptus cypellocarpa*. J Nat Prod. 2000;63: 1253–1257. doi:10.1021/np0001981

74. Rojas-Garbanzo C, Zimmermann BF, Schulze-Kaysers N, Schieber A. Characterization of phenolic and other polar compounds in peel and flesh of pink guava (Psidium guajava L. cv. 'Criolla') by ultra-high performance liquid chromatography with diode array and mass spectrometric detection. Food Research International. 2017;100: 445–453. doi:10.1016/j.foodres.2016.12.004

75. Arima H, Danno G. Isolation of Antimicrobial Compounds from Guava (*Psidium guajava* L.) and their Structural Elucidation. Biosci Biotechnol Biochem. 2002;66: 1727–1730. doi:10.1271/bbb.66.1727

76. Manso T, Lores M, de Miguel T. Antimicrobial Activity of Polyphenols and Natural Polyphenolic Extracts on Clinical Isolates. Antibiotics. MDPI; 2022. doi:10.3390/antibiotics11010046

77. Bouarab-Chibane L, Forquet V, Lantéri P, Clément Y, Léonard-Akkari L, Oulahal N, et al. Antibacterial properties of polyphenols: Characterization and QSAR (Quantitative structure-activity relationship) models. Front Microbiol. 2019;10. doi:10.3389/fmicb.2019.00829

78. Daglia M. Polyphenols as antimicrobial agents. Current Opinion in Biotechnology. 2012. pp. 174–181. doi:10.1016/j.copbio.2011.08.007

79. Ivanov M, Novović K, Malešević M, Dinić M, Stojković D, Jovčić B, et al. Polyphenols as Inhibitors of Antibiotic Resistant Bacteria—Mechanisms Underlying Rutin Interference with Bacterial Virulence. Pharmaceuticals. 2022;15. doi:10.3390/ph15030385

80. Kim D-H. Metabolism of Puerarin and Daidzin by Human Intestinal Bacteria and Their Relation to in Vitro Cytotoxity. 1998.

81. Xie Y, Yang W, Tang F, Chen X, Ren L. Antibacterial Activities of Flavonoids: Structure-Activity Relationship and Mechanism. Curr Med Chem. 2015;22: 132–149. doi:10.2174/0929867321666140916113443

82. Górniak I, Bartoszewski R, Króliczewski J. Comprehensive review of antimicrobial activities of plant flavonoids. Phytochemistry Reviews. 2019;18: 241–272. doi:10.1007/s11101-018-9591-z

83. Shamsudin NF, Ahmed QU, Mahmood S, Ali Shah SA, Khatib A, Mukhtar S, et al. Antibacterial Effects of Flavonoids and Their Structure-Activity Relationship Study: A Comparative Interpretation. Molecules. 2022;27: 1149. doi:10.3390/molecules27041149

84. Ulrey RK, Barksdale SM, Zhou W, van Hoek ML. Cranberry proanthocyanidins have anti-biofilm properties against Pseudomonas aeruginosa. BMC Complement Altern Med. 2014;14: 499. doi:10.1186/1472-6882-14-499

85. Song M, Liu Y, Li T, Liu X, Hao Z, Ding S, et al. Plant Natural Flavonoids Against Multidrug Resistant Pathogens. Advanced Science. 2021;8: 1–11. doi:10.1002/advs.202100749

86. Biharee A, Sharma A, Kumar A, Jaitak V. Antimicrobial flavonoids as a potential substitute for overcoming antimicrobial resistance. Fitoterapia. 2020;146: 104720. doi:10.1016/j.fitote.2020.104720

87. Amin MU, Khurram M, Khattak B, Khan J. Antibiotic additive and synergistic action of rutin, morin and quercetin against methicillin resistant Staphylococcus aureus. BMC Complement Altern Med. 2015;15: 59. doi:10.1186/s12906-015-0580-0

88. Haque E, Hussain J, Shankar S, Haldar S, Chatterjee S. Implication of Vibrio biofilms in human and seafood sector. Understanding Microbial Biofilms: Fundamentals to Applications. 2023; 247–260. doi:10.1016/B978-0-323-99977-9.00038-7

10 Figures

Figure 1. The response surfaces and estimated contours of the desirability value of *Vibrio spp* biofilm formation under different growth conditions to determine MIC.

Response surface for the interaction between the desirability of biofilm formation, based on the 3D plot of all combinations of four variables keeping two fixed variables (Vibrio species and initial inoculum) and two continuous variables (temperature and time) for evaluation. (a) Desirability of *Vibrio cholerae* biofilm formation with an initial inoculum of 0.05 McFarland versus temperature (24°C and 30°C) and time (24, 48 and 72 h). (b) Desirability of *Vibrio cholerae* biofilm formation with an initial inoculum of 0.50 McFarland versus temperature (24°C and 30°C) and time (24, 48 and 72 h). (c) Desirability of *Vibrio parahaemolyticus* biofilm formation with an initial inoculum of 0.05 McFarland versus temperature (24°C and 30°C) and time (24, 48 and 72 h). (d) Desirability of *Vibrio parahaemolyticus* biofilm formation with an initial inoculum of 0.50 McFarland versus temperature (24°C and 30°C) and time (24, 48 and 72 h).

Figure 2. Statistical analysis of the growth conditions involved in the biofilm formation of *Vibrio parahaemolyticus* and *Vibrio cholerae*.

The variables determined for biofilm formation of *Vibrio parahaemolyticus* (VP) and *Vibrio cholerae* (VC) were-temperature (24 and 30°C), initial inoculums (0.05 and 0.5 McFarland), and time (24, 48 and 72h). The biomass of the biofilm was analyzed by two methodologies measuring the optical density by absorbance at 630 nm with crystal violet and PBS suspension, while cell viability analysis was evaluated by colony-forming counting assays, and biofilm quantification was evaluated by total cell and live/dead cells assays. These results were analyzed using the Tukey and Fisher tests methodology for growth conditions comparison, where the bars show the mean of the variables for each result, in order to determine whether or not there is a significant difference between the variables (growth conditions). (a) Biofilm biomass by crystal violet (CV) staining method, (b) Biofilm biomass by phosphate-buffered saline (PBS) suspension method, (c) Biofilm viability by colony-forming (CFU) counting assays, (d) Biofilm quantification by live cell count with Live/Dead staining, (e) Biofilm quantification by dead cell count with Live/Dead staining, and (f) Biofilm quantification by total cell count with Live/Dead staining. * $p < 0.05$; ** $p < 0.01$; *** $p < 0.001$; **** $p < 0.0001$, - non–significant.

Figure 3. Illustration of fluorescence microscopy analysis in the biofilm formation of *Vibrio parahaemolyticus* and *Vibrio cholerae* at different growth conditions.

Biofilms of *Vibrio parahaemolyticus* and *Vibrio cholerae* at 24 and 30°C during time (24, 48, and 72h) by fluorescence microscopy using Live/Dead staining assays. The illustrated biofilm samples with an initial inoculum of 1E+8 CFU (0.5 McFarland) were used to compare the total number of live and dead cells. An Olympus BX50 microscope with 100X magnification was used (scale bar of 5 µm), images were obtained with AmScope software, and images were merged with Fiji-ImageJ software.

Figure 4. Inhibition of growth of *Vibrio parahaemolyticus* and *V. cholerae* strains (VP87, tetracycline resistant VP275, and VC112) by Eucalyptus (*a*) and Guava (*b*) extracts as well as tetracycline (*c*) and ceftriaxone (*d*) antibiotics at six given concentrations using micro-broth dilution assay in TSB supplemented with NaCl 1% after 24 h of incubation at 37 °C.

Bacterial turbidity was measured at OD = 630 nm and Minimum inhibitory concentration (MIC) values were determined for each plant extract and antibiotic against these planktonic Vibrio strains. The illustrated statistical analysis between biomass and treatment concentrations, for each Vibrio species. Using the Tukey and Fisher variable comparison methods to create confidence intervals for all pairwise differences in the means of the variable levels, where the bars show the average of the variables for each outcome, in order to determine whether or not there is a significant difference between variables (Control against treatment concentrations), more specifically *p < 0.05; ** p < 0.01; ***p < 0.001; ****p < 0.0001.

Figure 5. Illustration of the biomass evaluation at 630 nm and colony-forming units (CFU) counting in the biofilm inhibition assays of *Vibrio parahaemolyticus* and *V. cholerae* strains (VP87, tetracycline-resistant VP275, and VC112) by Eucalyptus (*a* and *e*) and Guava (*b* and *f*) extracts as well as tetracycline (*c* and *g*) and ceftriaxone (*d* and *h*) antibiotics at six given concentrations in TSB supplemented with NaCl 1% during 24 h of incubation at 24 °C.

Bacterial turbidity was measured at OD = 630 nm and Minimum inhibitory concentration (MIC) values were determined for each plant extract and antibiotic against these planktonic Vibrio strains. The illustrated statistical analysis between biomass and treatment concentrations, for each Vibrio species. Using the Tukey and Fisher variable comparison methods to create confidence intervals for all pairwise differences in the means of the variable levels, where the bars show the average of the variables for each outcome, in order to determine whether or not there is a significant difference between variables (Control against treatment concentrations), more specifically *p < 0.05; ** p < 0.01; ***p < 0.001; ****p < 0.0001.

Figure 6. Illustration of the biomass evaluation at 630 nm and colony-forming units (CFU) counting in the biofilm inhibition assays of *Vibrio parahaemolyticus* and *V. cholerae* strains (VP87, tetracycline-resistant VP275, and VC112) by Eucalyptus (*a* and *e*) and Guava (*b* and *f*) extracts as well as tetracycline (*c* and *g*) and ceftriaxone (*d* and *h*) antibiotics at six given concentrations in TSB supplemented with NaCl 1% during 72 h of incubation at 30 °C.

The illustrated statistical analysis between biomass and treatment concentrations, for each Vibrio species. Using the Tukey and Fisher variable comparison methods to create confidence intervals for all pairwise differences in the means of the variable levels, where the bars show the average of the variables for each outcome, in order to determine whether or not there is a significant difference between variables (Control against treatment concentrations), more specifically *p < 0.05; * * p < 0.01; ***p < 0.001; ****p < 0.0001.

Figure 7. Illustration of the biomass evaluation at 630 nm and colony-forming units (CFU) counting in the biofilm eradication assays of *Vibrio parahaemolyticus* and *V. cholerae* strains (VP87, tetracycline-resistant VP275, and VC112) by Eucalyptus (a and e) and Guava (*b* and *f*) extracts as well as tetracycline (*c* and *g*) and ceftriaxone (*d* and *h*) antibiotics at six given concentrations in TSB supplemented with NaCl 1% after 24 h of incubation at 24 °C.

The illustrated statistical analysis between biomass and treatment concentrations, for each Vibrio species. Using the Tukey and Fisher variable comparison methods to create confidence intervals for all pairwise differences in the means of the variable levels, where the bars show the average of the variables for each outcome, in order to determine whether or not there is a significant difference between variables (Control against treatment concentrations), more specifically *$p < 0.05$; * * $p < 0.01$; ***$p < 0.001$; ****$p < 0.0001$.

Figure 8. Illustration of the biomass evaluation at 630 nm and colony-forming units (CFU) counting in the biofilm eradication assays of *Vibrio parahaemolyticus* and *V. cholerae* strains (VP87, tetracycline-resistant VP275, and VC112) by Eucalyptus (a and e) and Guava (*b* and *f*) extracts as well as tetracycline (*c* and *g*) and ceftriaxone (*d* and *h*) antibiotics at six given concentrations in TSB supplemented with NaCl 1% after 72 h of incubation at 30 °C.

The illustrated statistical analysis between biomass and treatment concentrations, for each Vibrio species. Using the Tukey and Fisher variable comparison methods to create confidence intervals for all pairwise differences in the means of the variable levels, where the bars show the average of the variables for each outcome, in order to determine whether or not there is a significant difference between variables (Control against treatment concentrations), more specifically *$p < 0.05$; * * $p < 0.01$; ***$p < 0.001$; ****$p < 0.0001$.

11 Tables

Table 1. Metabolites identified in Guava and Eucalyptus extracts by HPLC-ESI-MS/MS in positive ionization mode.

ID	RT(min)	m/z [M+H]+	Fragmentation	Tentative identification
**Metabolites identified in Guava extract**				
1	13.78	435	303(100)<br>303-> 257(100), 165(80), 285(75), 229(50)	Reynoutrin or Avicularin
2	13.94	417	399(100), 351(30), 381(25), 297(15)	Puerarin
3	14.47	465	303(100)	Quercetin glucoside
4	15.31	419	287(100), 257(10)	Cyanidin 3-O-pentoside
5	15.84	587	285(100), 303(20)	Guavinoside C
6	16.16	449	317(100), 287(50)	petunidin-3-*O*-pentoside
7	16.35	545	527(100), 315(40), 273(30), 387(20)	Guvinoside A isomer
8	17.0	439	261(100), 421(10),	NI
9	17.3	439	369(100), 277(15), 259(10)	NI
10	17.9	573	259(100), 315(60), 297(20), 555(15), 181(10)<br>259-> 181(100), 105(15)	Guavinoside B
**Metabolites identified in Eucalyptus extract**				
1	14.45	611	303(100), 465(35), 593(10)	Rutin
2	14.50	463	287(100)	Luteolin-7-O-betaglucuronide
3	14.50	549	429(100), 379(80), 277(50), 361(45), 331(40), 531(35)	NI
4	14.55	479	303(100), 317(25)	Quercetin 3-O-glucuronide
5	16.42	477	301(100), 315(15), 459(10)	6-O-methylscutellarin
6	16.43	447	285(100)	Glycitin
7	18.11	631	303(100), 311(75), 613(55), 329(35), 595(20)	Cypellogin A or B
8	18.40	487	469(100), 311(60), 451(35)<br>311-> 149(100), 293(75), 167(15)	Like cypellocarpin C (see text)
9	19.31	615	287(100), 311(60), 597(35), 329(30)	Resinoside A
10	19.90	521	503(100), 485(20)<br>503-> 193(100), 311(40), 293(20), 167(15), 485(10)	cypellocarpin C
11	20.44	563	545(100)<br>545-> 425(100), 259(95), 301(65), 379(60), 527(55), 361(50), 191(45)	NI

Legend - NI: not identified

Figure 1

Click here to access/download;Figure;Figure_1-600dpi.jpg

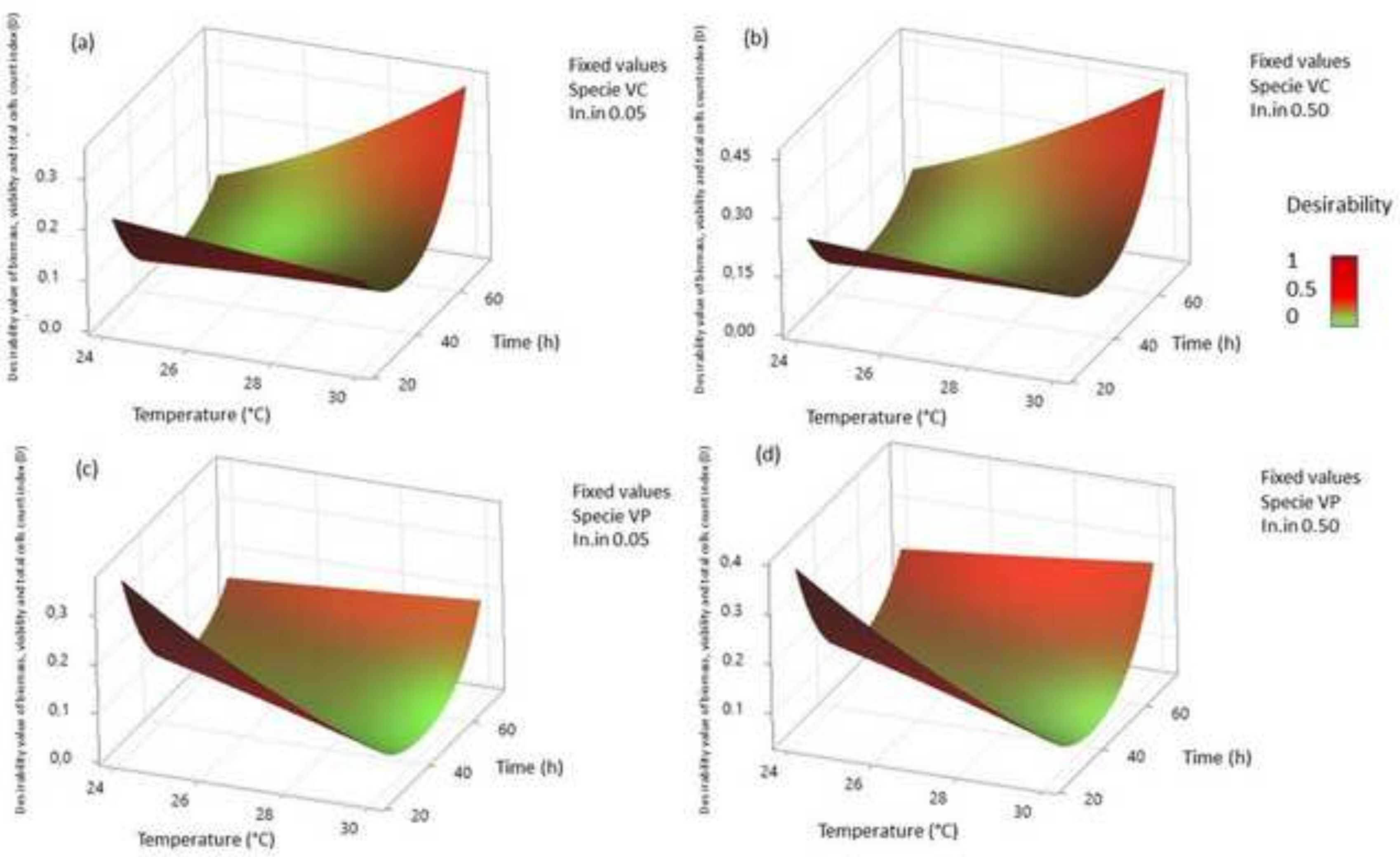

Figure 2 Click here to access/download;Figure;Figure_2-600dpi.jpg

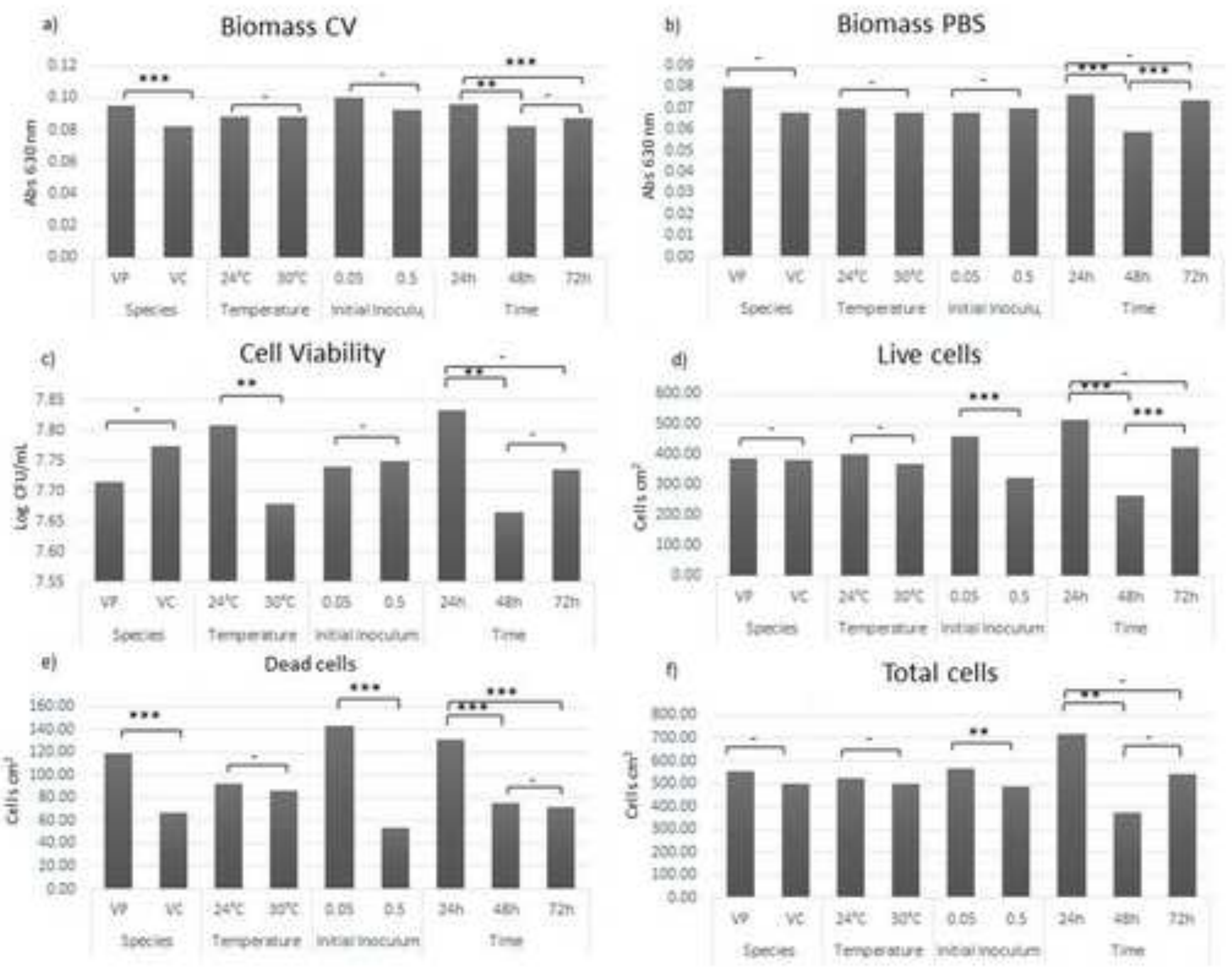

Figure 3

Click here to access/download;Figure;Figure_3-600dpi.jpg

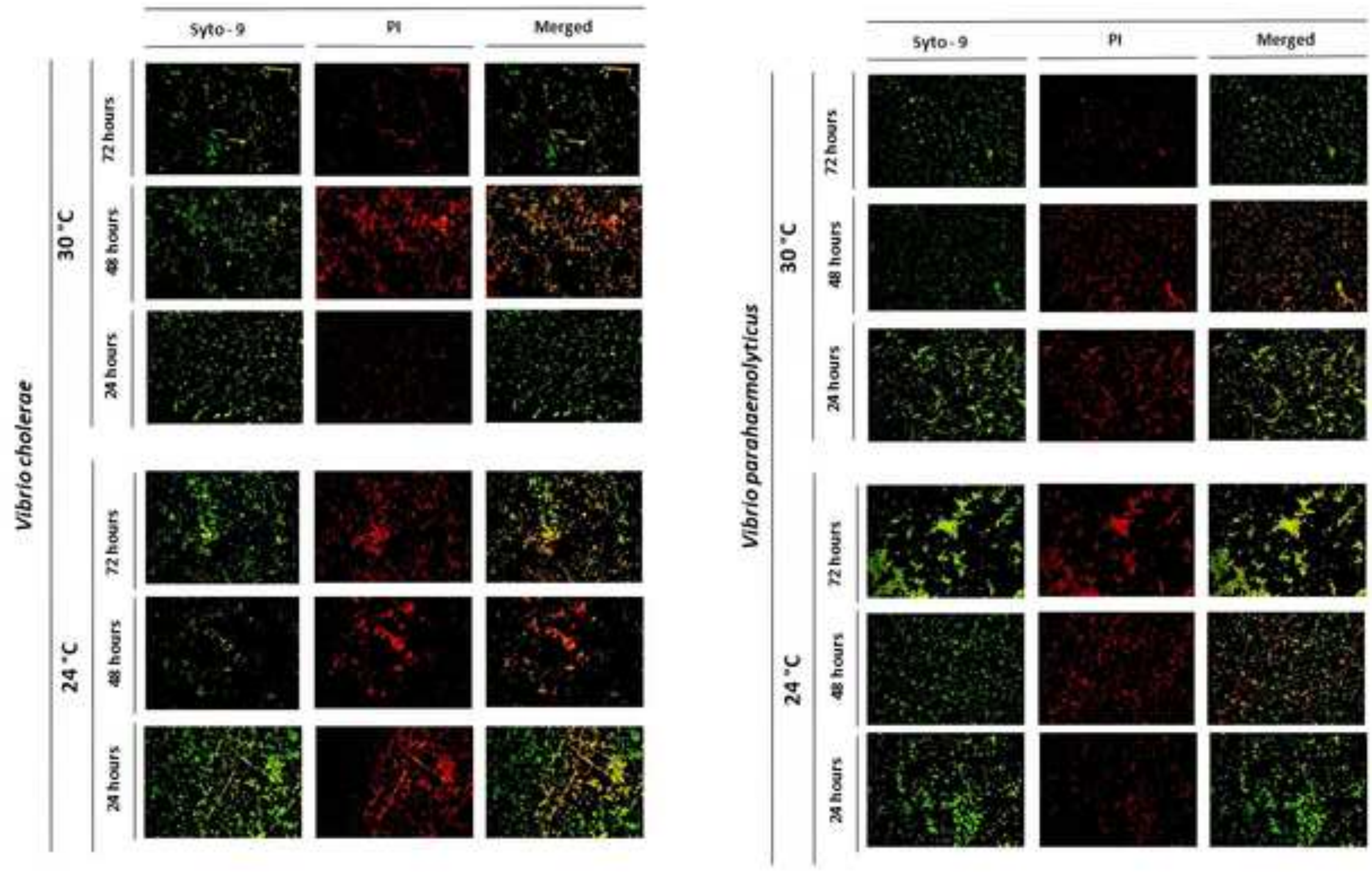

Figure 4

Click here to access/download;Figure;Figure_4-600dpi.jpg

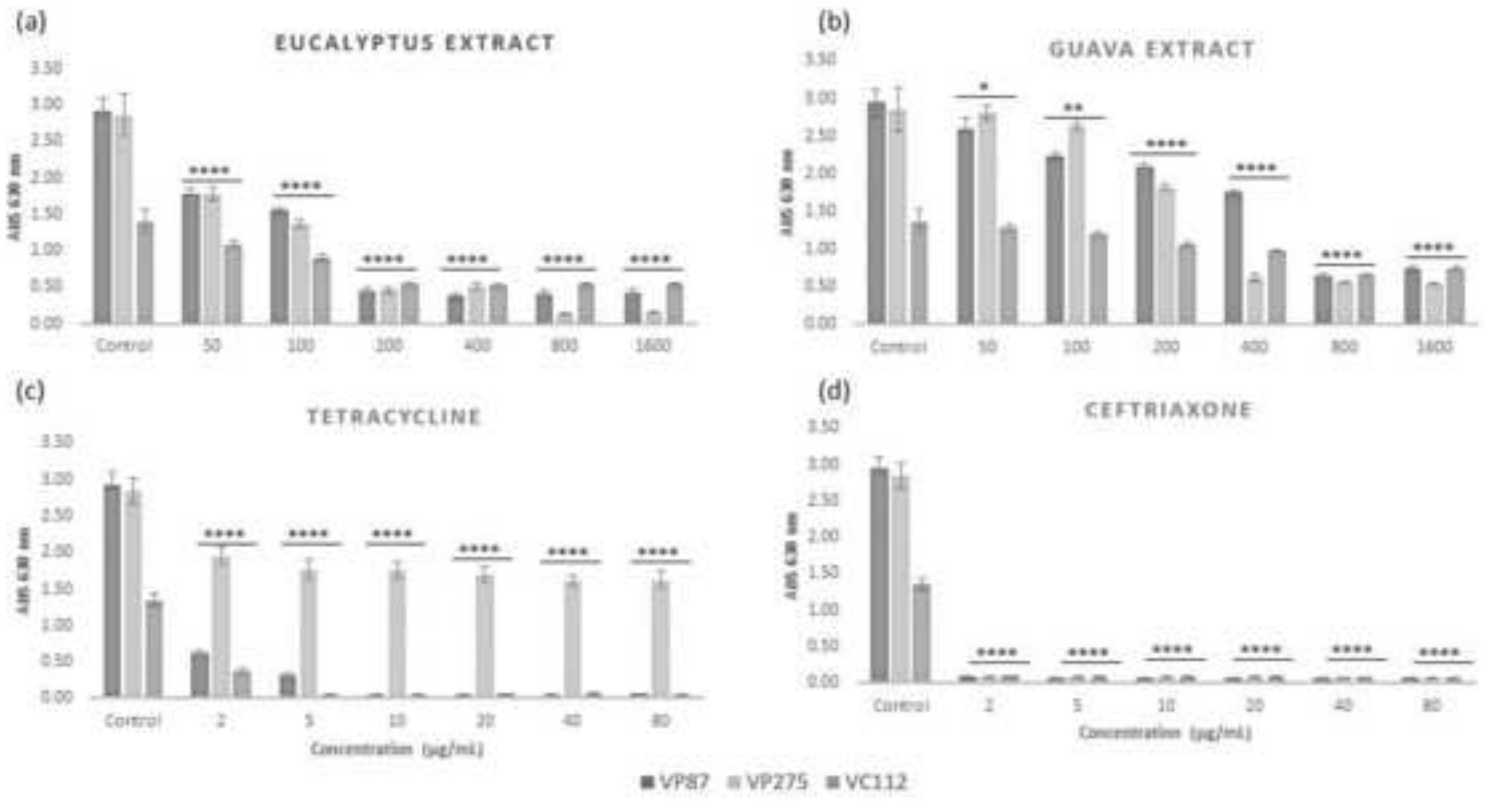

Figure 5

Click here to access/download;Figure;Figure_5-600dpi.jpg

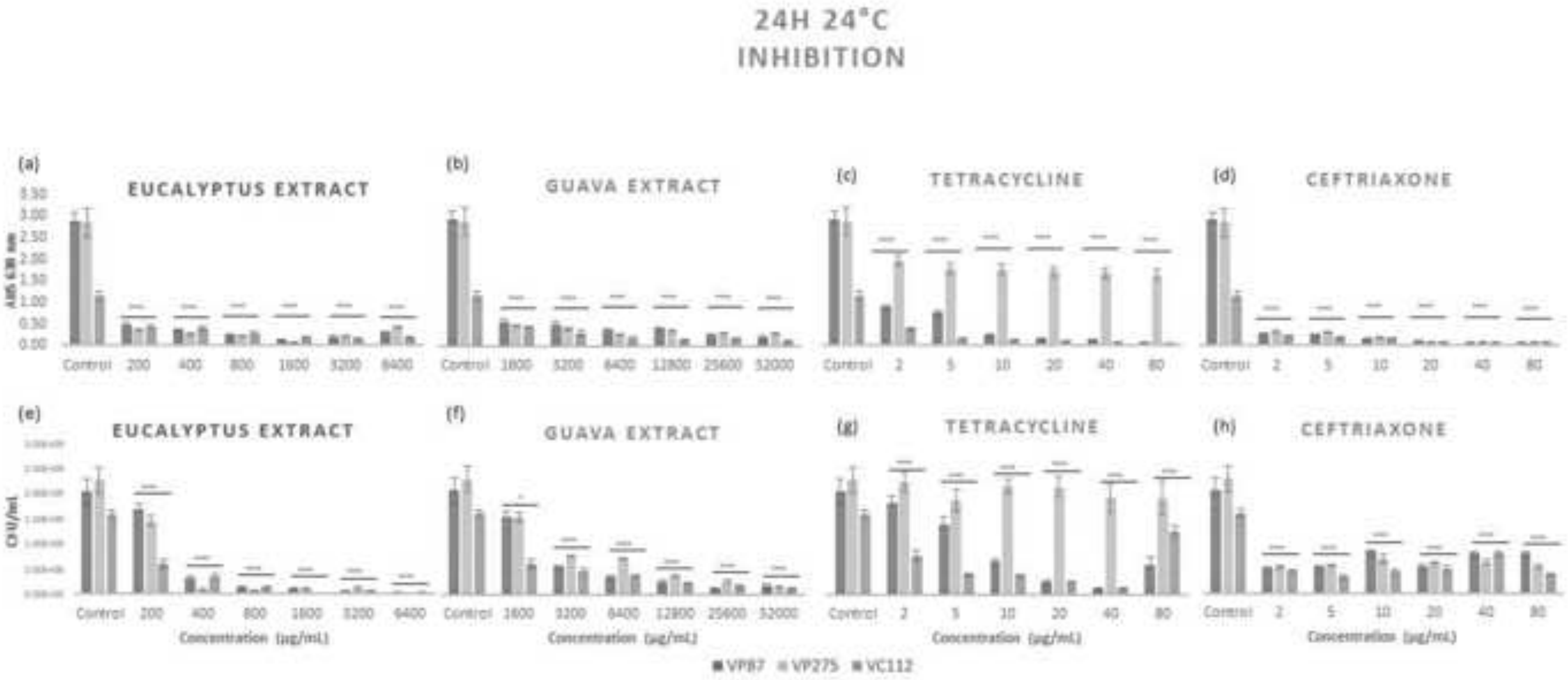

Figure 6

Click here to access/download;Figure;Figure_6-600dpi.jpg

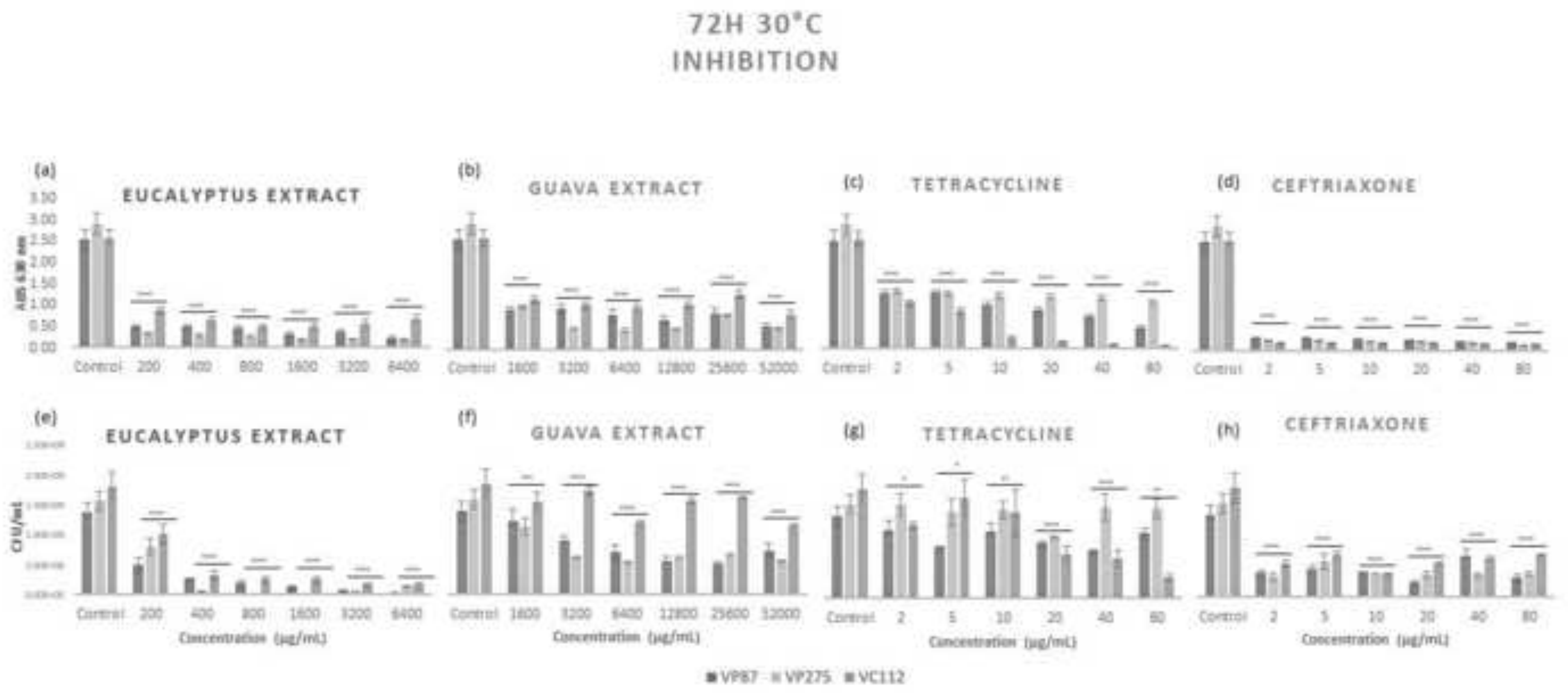

Figure 7

Click here to access/download;Figure;Figure_7-600dpi.jpg

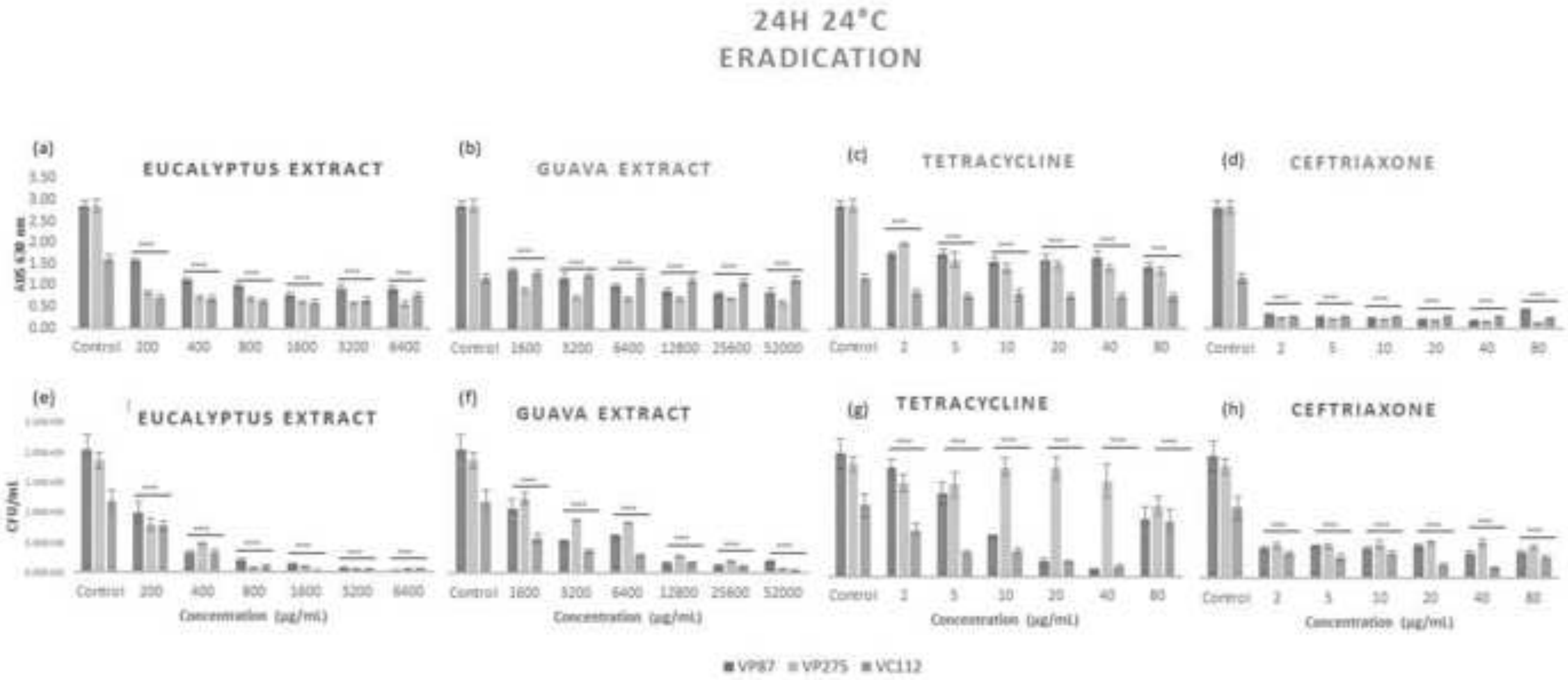

Figure 8
Click here to access/download;Figure;Figure_8-600dpi.jpg

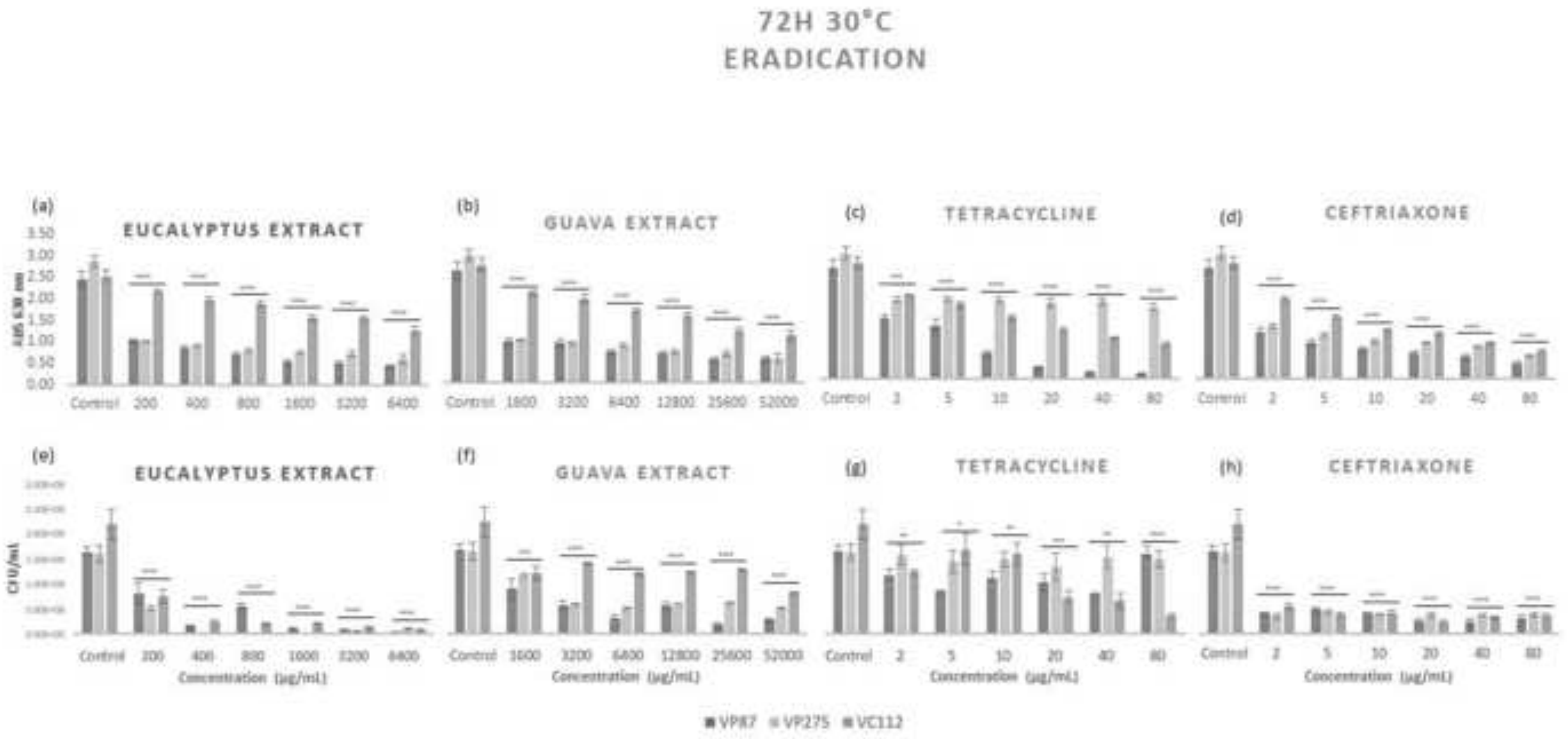

Table 1. Metabolites identified in Guava and Eucalyptus leaf extracts by HPLC-ESI-MS/MS in positive ionization mode.

ID	RT(min)	m/z [M+H]+	Fragmentation	Tentative identification
**Metabolites identified in Guava extract**				
1	13.78	435	303(100)<br>303-> 257(100), 165(80), 285(75), 229(50)	Reynoutrin or Avicularin
2	13.94	417	399(100), 351(30), 381(25), 297(15)	Puerarin
3	14.47	465	303(100)	Quercetin glucoside
4	15.31	419	287(100), 257(10)	Cyanidin 3-O-pentoside
5	15.84	587	285(100), 303(20)	Guavinoside C
6	16.16	449	317(100), 287(50)	petunidin-3-*O*-pentoside
7	16.35	545	527(100), 315(40), 273(30), 387(20)	Guvinoside A isomer
8	17.0	439	261(100), 421(10),	NI
9	17.3	439	369(100), 277(15), 259(10)	NI
10	17.9	573	259(100), 315(60), 297(20), 555(15), 181(10)<br>259-> 181(100), 105(15)	Guavinoside B
**Metabolites identified in Eucalyptus extract**				
1	14.45	611	303(100), 465(35), 593(10)	Rutin
2	14.50	463	287(100)	Luteolin-7-O-betaglucuronide
3	14.50	549	429(100), 379(80), 277(50), 361(45), 331(40), 531(35)	NI
4	14.55	479	303(100), 317(25)	Quercetin 3-O-glucuronide
5	16.42	477	301(100), 315(15), 459(10)	6-O-methylscutellarin
6	16.43	447	285(100)	Glycitin
7	18.11	631	303(100), 311(75), 613(55), 329(35), 595(20)	Cypellogin A or B
8	18.40	487	469(100), 311(60), 451(35)<br>311-> 149(100), 293(75), 167(15)	Like cypellocarpin C (see text)
9	19.31	615	287(100), 311(60), 597(35), 329(30)	Resinoside A
10	19.90	521	503(100), 485(20)<br>503-> 193(100), 311(40), 293(20), 167(15), 485(10)	Cypellocarpin C
11	20.44	563	545(100)<br>545-> 425(100), 259(95), 301(65), 379(60), 527(55), 361(50), 191(45)	NI

Legend - NI: Not Identified.

Click here to access/download
Supporting Information
Supplementary Material_Vibrio_PlosONE-Orig_V1.1.docx
