# Supplementary material for: Eco-alternative treatments for Vibrio parahaemolyticus and V. cholerae biofilms from shrimp industry through Eucalyptus (Eucalyptus globulus) and Guava (Psidium guajava) extracts: A road for an Ecuadorian sustainable economy
Source: PLoS One. 2024 Aug 13;19(8):e0304126. doi: 10.1371/journal.pone.0304126 (PMC11321589; doi:10.1371/journal.pone.0304126)
Supplement: Attachment — Submitted filename: Response to Reviewers-R1.docx [file pone.0304126.s011.docx]

**PONE-D-24-02362**

**Eco-alternative treatments for *Vibrio parahaemolyticus* and *V. cholerae* biofilms from shrimp industry through Eucalyptus (*Eucalyptus globulus*) and Guava (*Psidium guajava*) extracts: A road for an Ecuadorian sustainable economy**

Dear Editor and Reviewers,

Thank you very much for the comments regarding our original version of the submitted manuscript.

We have taken all reviewers’ comments and suggestions into careful consideration and revised the manuscript accordingly. On the following pages, please find our point-to-point responses to the editors’ and reviewers’ concerns in the order that they were originally listed, and details of the lines and pages on which the changes have been made.

It is important to mention that several rectifications were made according to reviewer concerns and comments. The major corrections were realized by adding the voucher specimens of Guava (*Psidium guajava* L.) and Eucalyptus (*Eucalyptus globulus* Labill.) leaf samples used in this study under the codes #35004 and #35005, respectively, and the authorization for collection of biological specimens (MAATE-ARSFC-2024-0328) issued by the Ministry of Environment, Water and Ecological Transition of Ecuador MAATE (see the new subsection 2.3 Ethics statement of the revised manuscript). This amendment was realized together with the Herbario de Botánica Económica del Ecuador of the USFQ; therefore, three coauthors were added to the revised manuscript due to the work involved in the present study. In addition, we improved the description of several procedures in the Materials and Methods section, and the resolution of figure pictures was also improved and corrected in the Results section through PLOS ONE guidelines and the Pace tool (<https://pacev2.apexcovantage.com/>), respectively. New information was added in the Introduction and Discussion sections about the Ecuadorian context and the Public Health threats involving *Vibrio parahaemolyticus* and *V. cholerae*, as well as the low-cost and available amount of Guava (*Psidium guajava*) and Eucalyptus (*Eucalyptus globulus)* plants as natural antimicrobial agents, cost-effective, sustainable, and environmentally friendly approach in Ecuador. Likewise, we added a new supplementary figure S1 to illustrate the descriptive HPLC chromatograms and the yield value of the plant extracts in the Results section. Several studies were added in the Introduction and Discussion sections to clarify the main goal and the novelty of the present study. Finally, we also realized intensive English editing together with English native speakers of our institution to improve the manuscript, checking the uniform format of all references and putting all biological names in the italic form.

We hope that the revised manuscript will be considered suitable for *PLOS ONE*’s publication criteria If there are further questions, please let us know. Thank you very much.

Sincerely,
António Machado (corresponding author)

**Corrected Manuscript**

**Detailed Response to Editor and Reviewers**

We want to thank the Editor and all reviewers for their constructive comments and thoughtful suggestions that allowed us to improve the original manuscript. Based on their comments and suggestions, we have made several modifications and corrections as described in the report below:

# **Reviewer 1**

***Comments to the Authors***

***Reviewer #1: Review comments for*** ***Plos One manuscript no. PONE-D-24-02362 “Eco-alternative treatments for Vibrio parahaemolyticus and V. cholerae biofilms from shrimp industry through Eucalyptus and Guava extracts: A road for an Ecuadorian sustainable economy.”***

***This paper evaluated the impact of temperature, time, and initial inoculum on the biofilm development of two Vibrio species, V. parahaemolyticus and V. cholerae, using a multifactorial experimental design. It also reports the effects of two aqueous extracts, Eucalyptus and Guava aqueous leaf extracts, as well as tetracycline and ceftriaxone, on bacterial biofilm formation and eradication. The paper is well written and the work, in general, is OK. However, there are some aspects that are not clear and need to be addressed before it is suitable for publication in PLOS ONE. Please see those comments, questions, and suggestions in the main manuscript file.***

**Authors' answers:** We want to thank Reviewer 1 for his/her constructive comments and thoughtful suggestions that allowed us to improve the original manuscript. All unclear aspects were addressed in the revised manuscript. We are pleased to inform you that thanks to your recommendations we have made the following changes:

***Lines 40-42- What is/are the rationale (s) for choosing these two species? V. parahaemolyticus form biofilm at a relatively high cell density, while V. cholerae form biofilm at low cell density. The genetic controls of their biofilm formations are different.***

**Authors' answers:** As well-appointed by Reviewer 1, the genetic controls of *V. parahaemolyticus* and *V. cholerae* biofilm formations are different, and detailed information was added in the Introduction section as well-suggested in a further comment (please check lines 104-113 on page 3 of the revised manuscript). Meanwhile, the rationale for choosing these two species is the endemic prevalence of these *Vibrio* species in the shrimp farms in Southern Coastal Ecuador, as previously reported by Ryan et al. (2018) and Sperling et al. (2015), and the collaboration partner Clydent SA suggestion to selecting these species isolated from shrimp farms. This information was added in the Abstract and Introduction section to clarify the non-familiar Readers of the Ecuadorian context. Thank you. Please check the amendments in lines 43-44 in the Abstract section and lines 121-123 on page 4 in the Introduction section of the revised manuscript.

References

Ryan SJ, Stewart-Ibarra AM, Ordóñez-Enireb E, Chu W, Finkelstein JL, King CA, Escobar LE, Lupone C, Heras F, Tauzer E, Waggoner E, James TG, Cárdenas WB, Polhemus M. Spatiotemporal Variation in Environmental *Vibrio cholerae* in an Estuary in Southern Coastal Ecuador. Int J Environ Res Public Health. 2018 Mar 10;15(3):486. doi: 10.3390/ijerph15030486.

Sperling L, Alter T, Huehn S. Prevalence and Antimicrobial Resistance of *Vibrio* spp. in Retail and Farm Shrimps in Ecuador. J Food Prot. 2015 Nov;78(11):2089-92. doi: 10.4315/0362-028X.JFP-15-160.

***Lines 42-44- Why use CFU, not their biofilm biomasses, instead?***

**Authors' answers:** As well-asked by Reviewer 1, the CFU counting assays were chosen as a reference because it is considered a more accurate quantitative methodology in the literature (such as Wilson et al., 2017 or Haas et al., 2023) and this approach was already realized in a previous study with *Candida*-related biofilms (Atiencia et al., 2022). However, CFU quantification allows to evaluate viable cells, but it cannot differentiate between live but non-culturable cells and dead cells. Therefore, CFU alone is not enough to assess biofilm inhibition and eradication, but it must be combined with other methodologies, such as biomass and LIVE/DEAD assays. The clarification of this limitation was added in lines 776-779 on page 19 of the Discussion section.

References

Atiencia-Carrera MB, Cabezas-Mera FS, Vizuete K, Debut A, Tejera E, Machado A. Evaluation of the biofilm life cycle between *Candida albicans* and *Candida tropicalis*. Front Cell Infect Microbiol. 2022 Aug 18;12:953168. doi: 10.3389/fcimb.2022.953168.

Wilson C, Lukowicz R, Merchant S, Valquier-Flynn H, Caballero J, Sandoval J, Okuom M, Huber C, Brooks TD, Wilson E, Clement B, Wentworth CD, Holmes AE. Quantitative and Qualitative Assessment Methods for Biofilm Growth: A Mini-review. Res Rev J Eng Technol. 2017 Dec;6(4): http://www.rroij.com/open-access/quantitative-and-qualitative-assessment-methods-for-biofilm-growth-a-minireview-.pdf.

Haas B, James S, Parker AE, Gagnon MC, Goulet N, Labrie P. Comparison of quantification methods for an endoscope lumen biofilm model. Biofilm. 2023 Oct 20;6:100163. doi: 10.1016/j.bioflm.2023.100163.

***Line 54- plant extracts?***

**Authors' answers:** As well-appointed by Reviewer 1, we replaced “extract plants” with “plant extracts”. Please check the amendment in lines 58-59 on page 2 of the revised manuscript. We apologize for this typo error and all text of the manuscript was carefully revised in the new version.

***Lines 95-99- The genetic controls of V. parahaemolyticus and V. cholerae biofilm formations are different. The authors should provide detailed information here.***

**Authors' answers:** We agree with Reviewer 1 recommendation and therefore we provided detailed information about the differences in genetic controls of *V. parahaemolyticus and V. cholerae* biofilm formations. Please check the new information in lines 104-113 on page 3 of the revised manuscript, more exactly:

“Although all *Vibrio* species are ubiquitous in aquatic ecosystems and can establish strong biofilms, *Vibrio* species can also differ in the mechanisms and regulation of their biofilm formation [19]. For example, *V. cholerae* biofilms are positively controlled by VpsR and VpsT regulators via the transcriptional control of *vps* genes, where VpsR is greater than VpsT. Meanwhile, *V. parahaemolyticus* biofilms are positively regulated via expression of *cps* genes, which are negatively regulated by a homolog of VpsT known as CpsS. In the absence of CpsS and CpsR (VpsR homolog), the positive *cps* gene expression leads to the *V. parahaemolyticus* biofilm formation. Thus, *V. cholerae* and *V. parahaemolyticus* use similar proteins, but they function in the opposite sense and to different degrees: CpsS is the dominant negative regulator in *V. parahaemolyticus* whereas VpsT is a positive co-regulator in *V. cholerae* [19,20].”

References

19.	Yildiz FH, Visick KL. *Vibrio* biofilms: so much the same yet so different. Trends in Microbiology. 2009. pp. 109–118. doi:10.1016/j.tim.2008.12.004 <https://pubmed.ncbi.nlm.nih.gov/19231189/>

20.	Teschler JK, Zamorano-Sánchez D, Utada AS, Warner CJA, Wong GCL, Linington RG, et al. Living in the matrix: Assembly and control of *Vibrio cholerae* biofilms. Nature Reviews Microbiology. Nature Publishing Group; 2015. pp. 255–268. doi:10.1038/nrmicro3433 <https://pubmed.ncbi.nlm.nih.gov/25895940/>

***Lines 100-102- Is this sentence correct? And please also cite the original work.***

**Authors' answers:** As well-suggested by Reviewer 1, we checked the data of the original sentence and we properly cited the original works, more exactly, Tran et al. (2013) and De Schryver et al. (2014). Please check the amendment in lines 114-117 on page 3 of the revised manuscript, more exactly:

**Original sentence**

“The main diseases that affect shrimp are early mortality shrimp-acute hepatopancreatic necrosis disease (EMS – AHPND) and causes 100% of mortality, while vibriosis causes 70% of mortality but damages the shrimp´s oral cavity and appendages [19].”

Original reference

Previous reference 19. Sajali USBA, Atkinson NL, Desbois AP, Little DC, Murray FJ, Shinn AP. Prophylactic properties of biofloc- or Nile tilapia-conditioned water against *Vibrio parahaemolyticus* infection of whiteleg shrimp (*Penaeus vannamei*). Aquaculture. 2019;498: 496–502. doi:10.1016/j.aquaculture.2018.09.002

**Revised sentence**

“The main diseases that affect shrimp are early mortality shrimp-acute hepatopancreatic necrosis disease (EMS – AHPND) affects shrimp postlarvae within 20–30 days after stocking and frequently causes up to 100% mortality [21,22], while the mortality of vibriosis can range between 70-100% but damages the shrimp´s oral cavity and appendages [5,23].”

**Updated and rectified references**

21.	De Schryver P, Defoirdt T, Sorgeloos P. Early Mortality Syndrome Outbreaks: A Microbial Management Issue in Shrimp Farming? PLoS Pathog. 2014;10. doi:10.1371/journal.ppat.1003919 <https://doi.org/10.1371/journal.ppat.1003919>

22.	Tran L, Nunan L, Redman RM, Mohney LL, Pantoja CR, Fitzsimmons K, et al. Determination of the infectious nature of the agent of acute hepatopancreatic necrosis syndrome affecting penaeid shrimp. Dis Aquat Organ. 2013;105: 45–55. doi:10.3354/dao02621 <https://pubmed.ncbi.nlm.nih.gov/23836769/>

23.	De La Peña LD, Cabillon NAR, Catedral DD, Amar EC, Usero RC, Monotilla WD, et al. Acute hepatopancreatic necrosis disease (AHPND) outbreaks in *Penaeus vannamei* and *P. monodon* cultured in the Philippines. Dis Aquat Organ. 2015;116: 251–254. doi:10.3354/dao02919 <https://pubmed.ncbi.nlm.nih.gov/26503780/>

5. Sanches-Fernandes GMM, Sá-Correia I, Costa R. Vibriosis Outbreaks in Aquaculture: Addressing Environmental and Public Health Concerns and Preventive Therapies Using Gilthead Seabream Farming as a Model System. Front Microbiol. 2022;13. doi:10.3389/fmicb.2022.904815

***Lines 102-104- Please also check this sentence for accuracy, and the paper cited. It is well known that V. harveyi and V. parahaemolyticus are the two most important Vibrio species that cause disease in shrimp.***

**Authors' answers:** As well-appointed by Reviewer 1, we examined the accuracy of the original sentence and we rectified and properly cited the original works, more exactly, Ryan et al. (2018) and Sperling et al. (2015). We rectified the main shrimp-related pathogens and the human-related infections, also explaining the Ecuadorian context and justifying the selection of *V. parahaemolyticus* and *V. cholerae* in the present study. We apologize for the mistake and confusing sentence. Please check the amendment in lines 117-123 on pages 3-4 of the revised manuscript, more exactly:

**Original sentence**

“These diseases are caused by pathogenic *Vibrio* species, being *Vibrio alginolyticus*, *V. parahaemolyticus*, *V. cholerae*, and *V. vulnificus* the main pathogens isolated [16].”

Original reference

16. Flemming H-C, van Hullebusch ED, Neu TR, Nielsen PH, Seviour T, Stoodley P, et al. The biofilm matrix: multitasking in a shared space. Nature Reviews Microbiology 2022. 2022; 1–775 17. doi:10.1038/s41579-022-00791-0

**Revised sentence**

“Therefore, several *Vibrio* species can significantly reduce shrimp production where *V. parahaemolyticus*, *V. harveyi*, *V. alginolyticus*, and *V. vulnificus* are commonly isolated in these shrimp farms [24]. In addition, certain *Vibrio* species can emerge as a serious threat to human health, such as *V. parahaemolyticus*, *V. cholerae*, and *V. alginolyticus* [25]. In Ecuador, the prevalence of *V. parahaemolyticus* and *V. cholerae* in shrimps at retail, farms, and estuaries are alarming and represent an eminent and serious public health risk [26,27].”

**Updated and rectified references**

24.	de Souza Valente C, Wan AHL. Vibrio and major commercially important vibriosis diseases in decapod crustaceans. J Invertebr Pathol. 2021;181. doi:10.1016/j.jip.2020.107527

25.	Robert-Pillot A, Copin S, Himber C, Gay M, Quilici ML. Occurrence of the three major *Vibrio* species pathogenic for human in seafood products consumed in France using real-time PCR. Int J Food Microbiol. 2014;189: 75–81. doi:10.1016/j.ijfoodmicro.2014.07.014

26.	Ryan SJ, Stewart-Ibarra AM, Ordóñez-Enireb E, Chu W, Finkelstein JL, King CA, et al. Spatiotemporal variation in environmental *Vibrio cholerae* in an estuary in southern coastal Ecuador. Int J Environ Res Public Health. 2018;15. doi:10.3390/ijerph15030486. <https://pubmed.ncbi.nlm.nih.gov/29534431/>

27.	Sperling L, Alter T, Huehn S. Prevalence and antimicrobial resistance of *Vibrio* spp. in retail and farm shrimps in Ecuador. J Food Prot. 2015;78: 2089–2092. doi:10.4315/0362-028X.JFP-15-160. <https://pubmed.ncbi.nlm.nih.gov/26555534/>

***Lines 112-113- “Some alternative strategies to control Vibrio species and their biofilm formation is using probiotics and natural products in mature ecosystems [2,6].” Please check this "Yatip, P., Teja, D. Nitin Chandra, Flegel, T. W. and Soowannayan, C. (2018) Extract from the fermented soybean product Natto inhibits Vibrio biofilm formation and reduces shrimp mortality from Vibrio harveyi infection, Fish & Shellfish Immunology 72:348-355" and other papers from the same group.***

**Authors' answers:** As well-recommended by Reviewer 1, we added some studies on alternative strategies to control *Vibrio*-related biofilms or infections, such as Yatip et al. (2018), Karnjana et al. (2019), and Kamble et al. (2022). Thank you for your recommendations that allow us to improve the original manuscript. Please check this new information in lines 132-142 on page 4 of the revised manuscript, more exactly:

“Some alternative strategies to control *Vibrio* species and their biofilm formation is using probiotics and natural products in mature ecosystems [2,6]. In 2018, Yatip et al. discovered that extract from the fermented soybean product Natto (generally regarded as safe, GRAS) can inhibit *Vibrio* biofilm formation significantly reducing shrimp mortality with *V. harveyi* infection [29]. Likewise, Karnjana et al. reported that the ethanolic extract of the red seaweed *Gracilaria fisheri* enhanced immune activities and inhibited the growth of *V. harveyi* in shrimp suggesting a potential application of this *G. fisheri* extract as an efficient approach for treating biofilm-associated *Vibrio* diseases in aquacultures [30]. Furthermore, Kamble et al. explored sulphated galactans degraded fractions of the *G. fisheri* evidencing a stronger antibacterial activity against *V. parahaemolyticus* and *V. harveyi* through the disruption of the bacterial cell membrane resulting in leakage of intracellular biological components and thus cell death [31].

**Added references**

29. Yatip P, Nitin Chandra Teja D, Flegel TW, Soowannayan C. Extract from the fermented soybean product Natto inhibits *Vibrio* biofilm formation and reduces shrimp mortality from *Vibrio harveyi* infection. Fish Shellfish Immunol. 2018 Jan;72:348-355. doi: 10.1016/j.fsi.2017.11.008. <https://pubmed.ncbi.nlm.nih.gov/29127029/>

30. Karnjana K, Soowannayan C, Wongprasert K. Ethanolic extract of red seaweed Gracilaria fisheri and furanone eradicate *Vibrio harveyi* and *Vibrio parahaemolyticus* biofilms and ameliorate the bacterial infection in shrimp. Fish Shellfish Immunol. 2019 May;88:91-101. doi: 10.1016/j.fsi.2019.01.058. <https://pubmed.ncbi.nlm.nih.gov/30817993/>

31. Kamble MT, Rudtanatip T, Soowannayan C, Nambunruang B, Medhe SV, Wongprasert K. Depolymerized Fractions of Sulfated Galactans Extracted from *Gracilaria fisheri* and Their Antibacterial Activity against *Vibrio parahaemolyticus* and *Vibrio harveyi*. Mar Drugs. 2022 Jul 23;20(8):469. doi: 10.3390/md20080469. <https://pubmed.ncbi.nlm.nih.gov/35892937/>

***Lines 120-121- What is the rationale for choosing these two species? V. parahaemolyticus forms biofilm at a relatively high cell density, while V. cholerae form biofilm at low cell density. The controls of their biofilm formations are different.***

**Authors' answers:** As previously asked and well-suggested by Reviewer 1, the rationale for choosing *V. parahaemolyticus* and *V. cholerae* was explained in lines 121-123 on page 4 while the genetic controls of their biofilm formations were added in lines 104-113 on page 3 in the Introduction section of the revised manuscript. Please check these amendments and explanations in the revised manuscript and the previous answers to your comments. Thank you.

***Lines 133-135- Are these isolates shrimp pathogens? Were they originated from infected or diseased shrimp?***

**Authors' answers:** As well-observed by Reviewer 1, we did not specify that all three *Vibrio* strains were isolated from shrimp farms by Clydent S.A. laboratory, more exactly from diseased shrimp samples, and then the isolated vials were sent to the Institute Microbiology for further analysis. We apologize for this lapse, and we added this information to the revised manuscript. Please check the amendment in lines 161-167 on page 5 of the revised manuscript, more exactly:

**Original sentence**

“*Vibrio cholerae* and *V. parahaemolyticus* isolates from the microbial collection of the Institute of Microbiology Universidad San Francisco de Quito (IM-USFQ), designated as IMUSFQ-VC112 and IMUSFQ-VP 87, respectively, were selected for the present study. These *Vibrio* isolates were previously recovered from El Oro, Santa Elena, and Guayas and identified through DNA sequences at multiple loci and biochemical properties at the Clydent S.A laboratory (<https://clydent.com/>) and IM-USFQ.”

**Revised sentence**

“*V. parahaemolyticus* and *V. cholerae* strains from the microbial collection of the Institute of Microbiology Universidad San Francisco de Quito (IM-USFQ), designated as IMUSFQ-VP87, tetracycline-resistant IMUSFQ-VP275, and IMUSFQ-VC112, respectively, were selected for the present study. These *Vibrio* isolates were previously recovered by Clydent S.A. laboratory (<https://clydent.com/>) from diseased shrimp samples of shrimp farms located in El Oro, Santa Elena, and Guayas provinces, and then identified through DNA sequences at multiple loci and biochemical properties at the Clydent S.A. laboratory and IM-USFQ.”

***Lines 151-152- “Soxhlet extractions were carried out with 10 grams of dry biomass and 300 mL of distilled water for 3 h.” temperature?***

**Authors' answers:** As well-detected by Reviewer 1, the temperature of the Soxhlet extractions was not mentioned. Thank you for alerting us of this mistake. We apologized and we added the temperature of 80ºC to the sentence. Please check the amendment in lines 183-184 on page 5 of the revised manuscript, more exactly:

**Revised sentence**

“Soxhlet extractions at 80 °C were carried out with 10 grams of dry biomass and 300 mL of distilled water for 3 h.”

***Lines 153-157- Were any pure compounds or known standard compounds used in these analyses?***

**Authors' answers:** As well-questioned by Reviewer 1, no pure or known standard compounds were used in the present study. The use of HPLC-MS/MS for compound identification is quite common in our lab and we used to run several pure compounds of different types in order to validate ruptures (when possible) or help us to define chromatographic regions in which we usually found different chemical groups. In this sense, common natural products, such as quercetin, pelargonidin (as anthocyanin representation), luteolin, and rutin, are used in our lab. However, in the particular case of the guavinoside and cypellocarpin compounds family, we do not have pure compounds. These molecules were identified exclusively using databases and mainly comparing our ruptures with previous reports in these plants. This information was disclosed in lines 204-206 on page 6 of the subsection 2.2 of the revised manuscript, more exactly:

**Lines 204-206 on page 6 of the subsection 2.2 of the revised manuscript**

“The peak tentative identification was exclusively done using MzCloud (https://www.mzcloud.org/) and Global Natural Products Social molecular networking (GNPS, http://gnps.ucsd.edu) databases as well as the scientific literature.”

***Lines 180-181- How were these biofilm assays done precisely?***

**Authors' answers:** As well-quizzed by Reviewer 1, the initial inoculums for biofilm assays were previously described in subsection 2.1 entitled “bacterial isolates and growth conditions” in lines 160-176 on pages 4-5; while the general settings of biofilms assays were described in subsection 2.4 entitled “Biofilm assays” in lines 214-225 on page 6; and finally, each precise of differential steps were carefully explained in the following subsections “2.5 Biomass quantification” (lines 227-230 on page 6), “2.5 Viability quantification” (lines 256-84 on pages 7), and “2.7.2 Biofilm inhibition and eradication assays” (lines 330-350 on page 9). We apologize for the extensive and divided description of the biofilms assays but 2 biomass procedures and 2 viability methodologies together with multifactorial variables (2 *Vibrio* species, 2 temperatures, 2 initial inoculums, and 3 times) and biofilm inhibition and eradication assays were done in the present study. So, we were not able to completely describe all the steps in subsection “2.4 Biofilm assays”. The biofilm assays with different methodologies were similarly described in our previous studies. Please consult the following open-access studies:

<https://www.frontiersin.org/articles/10.3389/fcimb.2022.953168/full>

<https://www.sciencedirect.com/science/article/pii/S2665927123001119?via%3Dihub>

We apologize for the inconvenience and we hope that you understand why we had to split the different descriptions of each methodology applied to the biofilm assays.

***Lines 371-408 at subsection 3.1 Identification of chemical compounds in Guava and Eucalyptus leaf extracts- Why didn't the authors test each fraction for their biofilm-inhibiting and growth-inhibiting activities?***

**Authors' answers:** As well-appointed by Reviewer 1, it is a very important topic. We did not fraction the extract because this process requires more sample materials and a preparative HPLC (or similar equipment). In this study, we used HPLC with analytic conditions and columns. Currently, we are working on the fractioning procedure, as well as on the acquisition of the pure compounds to validate/evaluate the candidate molecules for further fractions activity. These limitations were included in the manuscript in order to be clear on the scope of the work and to open further research analysis. Please check lines 767-773 on page 19 at the end of the Discussion section in the revised manuscript, more exactly:

**Lines 767-773 on page 19 at the end of the Discussion section in the revised manuscript**

“Limitations and further recommendations

In the present work, we indicate the biological activity of the Eucalyptus (*Eucalyptus globulus*) and Guava (*Psidium guajava*) leaf extracts. However, we don’t know which of the identified compounds is responsible for the activity. Further studies need to be done to fraction the extracts with further activity evaluation and/or the direct biological activity evaluation of the pure identified compounds. Moreover, future experiments could be designed to determine the mechanism behind biological activity.”

***Lines 411-413- What are these biomass data in the supplementary table? How were they generated?***

**Authors' answers:** As well-inquired by Reviewer 1, the biomass data showed in Supplementary Tables S2 and S3 in the Supplementary Information section corresponds to the Biomass PBS at abs 630 nm adjusted (Supplementary Tables S2 and S3) and Biomass crystal violet (CV) staining at abs 630 nm adjusted (Supplementary Table S3). More exactly, the abs lectures of biomass by PBS suspension and CV staining were adjusted by subtracting the absorbance measurements of sterility control (media without bacterial growth) from the absorbance measurements of samples. We apologize for this missing information. We added this information about the abs adjustment in the main text of the revised manuscript and legends of Supplementary Tables S2 and S3, more exactly:

The main manuscript in the subsections:

**2.5.1** **Crystal violet staining**

**Lines 243-245 on page 7:** “All OD measurements by CV staining were adjusted by subtracting the absorbance measurements of sterility controls from the absorbance measurements of biofilm samples.”

**2.5.2 Phosphate-buffered saline suspension**

**Lines 252-254 on page 7:** “All OD measurements by PBS suspension were adjusted by subtracting the absorbance measurements of sterility controls from the absorbance measurements of biofilm samples.”

**The Supplementary Information section**

**Legend of the Supplementary Table S3**

**Lines 36-37 on page 5:** “All OD measurements by PBS suspension and CV staining were adjusted by subtracting the absorbance measurements of sterility controls from the absorbance measurements of biofilm samples.”

***Lines 423-426- “Normality tests showed normal distribution after the reduction of the model in biomass and viability tests, but a non-normal distribution in the total cell count test, so a data transformation by Johnson's method was performed. Consequently, a parametric statistical analysis was selected for future evaluation.” Has this viability staining method been optimized for these two bacterial species?***

**Authors' answers:** As well-quizzed by Reviewer 1, the viability staining method was previously optimized for these *Vibrio* species (data not shown) using DAPI staining to validate the total cell count within biofilm samples and further compared with the total cell count obtained by LIVE/DEAD methodology, as realized for other microorganisms in our previous studies. Please consult the following open-access studies:

<https://www.frontiersin.org/articles/10.3389/fcimb.2022.953168/full>

<https://www.sciencedirect.com/science/article/pii/S2665927123001119?via%3Dihub>

It is well known that LIVE/DEAD staining provided information on how many of the total cells were dead and alive within biofilms through their capacity to exclude, accumulate, and metabolize the fluorophores SYTO-9 and PI. However, as expected, LIVE/DEAD staining was the most variable of the methodologies used in this work, as previously suggested in other studies (Rosenberg et al., 2019; McGoverin et al., 2020).

References

Rosenberg, M., Azevedo, N. F., Ivask, A. (2019). Propidium iodide staining underestimates viability of adherent bacterial cells. Sci. Rep. 9, 1–12. doi: 10.1038/s41598-019-42906-3

McGoverin, C., Robertson, J., Jonmohamadi, Y., Swift, S., Vanholsbeeck, F. (2020). Species dependence of SYTO 9 staining of bacteria. Front. Microbiol. 11. doi: 10.3389/fmicb.2020.545419

As recommended by Reviewer 1, this information was incorporated into the Discussion section. Please check lines 650-658 on page 17 in the Discussion section of the revised manuscript, more exactly:

**Lines 650-658** **on page 17 in the Discussion section of the revised manuscript**

“However, it is important to mention that, during the realization of biofilm assays, *V. parahaemolyticus* evidenced thicker biofilms during the first 24 h but *V. cholerae* biofilms showed more resilience to the washing steps during time (72 h) which could influence the results when comparing the four methodologies to assess biofilm development. Due to the observed biofilm thickness in both *Vibrio* species, the fluorescence microscopy analysis through LIVE/DEAD assays could be affected by the dye infiltration (SYTO-9 and PI) within the samples resulting in a lower R-squared value (R^2^ = 0.22 - 0.45) for biofilm analysis. Although the LIVE/DEAD staining assay was previously optimized (data not shown), the present methodology requires to be combined with other methodologies, as previously observed [67,68].”

***Lines 466-467- “Over time V. parahaemolyticus showed a higher number of dead cells within the biofilm (around 44%) when compared to V. cholerae (p <0.001).” How many percent?***

**Authors' answers:** As well-appointed by Reviewer 1, we failed to state the percent of dead cells in *V. cholerae* biofilm samples and the percentage of dead cells in *V. parahaemolyticus* biofilm samples was wrongly calculated. We apologize for these mistakes and we thank you for the correction. The original sentence was rectified in the revised manuscript. Please check the revised sentence in lines 512-515 on page 13 of the revised manuscript, more exactly:

**Lines 512-515 on page 13 of the revised manuscript**

“More exactly, *V. parahaemolyticus* showed a higher number of dead cells within the biofilm (9.09E+5 dead cells/cm^2^) and approximately 21% of the total cells, while *V. cholerae* only showed 13% of dead cells (5.05E+5 dead cells/cm^2^) within the biofilm (*p* <0.001).”

***Lines 494-497- “As expected, fluorescence microscopy analysis revealed the lowest R-squared values for both Vibrio related biofilms (R2 = 0.22 - 0.45), followed by biomass assays with CV staining (R2 = 0.84 - 0.85), and finally biomass assays with PBS suspension (R2 = 0.86 - 0.92).” Exact question: has this method been optimized for these two bacterial biofilms? Do they form similar biofilms, structurally, as well as their thicknesses? The biofilm thickness will undoubtedly affect the dye infiltration and staining.***

**Authors' answers:** As well-appointed by Reviewer 1 and answered in a previous comment, the viability staining method was previously optimized for these *Vibrio* species (data not shown) using DAPI staining to validate the total cell count within biofilm samples and further compared with the total cell count obtained by LIVE/DEAD methodology. However, Reviewer 1 raised a very good question that was not discussed in the original manuscript. Indeed, we observed during biofilm assays that *V. parahaemolyticus* evidenced thicker biofilms during the first 24 h but *V. cholerae* biofilms showed more resilience to the washing steps during time (72 h). It is worth discussing this information in the Discussion section, as well-stated by Reviewer 1, due to the biofilm thickness surely affecting the dye infiltration and staining of biofilm samples. Thank you for calling our attention to this important issue that could explain the results obtained in the present study.

So, we incorporated the report on this factor in lines 650-658 on page 17 in the Discussion section of the revised manuscript, more exactly:

“However, it is important to mention that, during the realization of biofilm assays, *V. parahaemolyticus* evidenced thicker biofilms during the first 24 h but *V. cholerae* biofilms showed more resilience to the washing steps during time (72 h) which could influence the results when comparing the four methodologies to assess biofilm development. Due to the observed biofilm thickness in both *Vibrio* species, the fluorescence microscopy analysis through LIVE/DEAD assays could be affected by the dye infiltration (SYTO-9 and PI) within the samples resulting in a lower R-squared value (R^2^ = 0.22 - 0.45) for biofilm analysis. Although the LIVE/DEAD staining assay was previously optimized (data not shown), the present methodology requires to be combined with other methodologies, as previously observed [67,68].”

**References**

67.	McGoverin C, Robertson J, Jonmohamadi Y, Swift S, Vanholsbeeck F. Species Dependence of SYTO 9 Staining of Bacteria. Front Microbiol. 2020;11: 545419. doi:10.3389/fmicb.2020.545419

68.	Stiefel P, Schmidt-Emrich S, Maniura-Weber K, Ren Q. Critical aspects of using bacterial cell viability assays with the fluorophores SYTO9 and propidium iodide. BMC Microbiol. 2015;15: 36. doi:10.1186/s12866-015-0376-x

***Discussion at pages 15-17- Please consider questions and comments in previous sections and incorporate them into the discussion.***

**Authors' answers:** As well-advised by Reviewer 1, we added a more profound discussion of the results obtained in the present work based on the Reviewer’s questions and comments. More exactly, the rationale for selecting *V. parahaemolyticus* and *V. cholerae* was added after the economic losses in the shrimp industry in lines 639-642 on page 16 in the Discussion section. Also, we discussed the thickness of biofilm samples affecting the dye infiltration and staining of biofilm samples and therefore resulting in a lower R-squared value (R^2^ = 0.22 - 0.45) for biofilm analysis in lines 650-658 on page 17 at Discussion, as replied in our previous answer. We also added the discussion of the different genetic controls in promoting and regulating biofilms of *V. parahaemolyticus* and *V. cholerae* to clarify the results obtained 660-664 on page 17 in the Discussion section. Finally, we discussed previous studies with alternative strategies to control *Vibrio*-related infections, more exactly Yatip et al. (2018), Karnjana et al. (2019), and Kamble et al. (2022), justifying the selection of Eucalyptus and Guayaba due to their well-known antibacterial activity by other previous studies (Gosh et al., 2021a and 2021b; Zheng et al., 2020; Dewi et al., 2021; Mahfuzul Hoque et al., 2007) and their abundance in Ecuador to be applied in the shrimp farms. Please check this new discussion in lines 672-687 on page 17 of the revised manuscript. Finally, we also acknowledged several limitations of the present study and clarified the need for further analysis in future studies. Please check these limitations and further recommendations in lines 767-779 on page 19 of the revised manuscript. Thank you!

**New added references**

29. Yatip P, Nitin Chandra Teja D, Flegel TW, Soowannayan C. Extract from the fermented soybean product Natto inhibits *Vibrio* biofilm formation and reduces shrimp mortality from *Vibrio harveyi* infection. Fish Shellfish Immunol. 2018 Jan;72:348-355. doi: 10.1016/j.fsi.2017.11.008. <https://pubmed.ncbi.nlm.nih.gov/29127029/>

30. Karnjana K, Soowannayan C, Wongprasert K. Ethanolic extract of red seaweed Gracilaria fisheri and furanone eradicate *Vibrio harveyi* and *Vibrio parahaemolyticus* biofilms and ameliorate the bacterial infection in shrimp. Fish Shellfish Immunol. 2019 May;88:91-101. doi: 10.1016/j.fsi.2019.01.058. <https://pubmed.ncbi.nlm.nih.gov/30817993/>

31. Kamble MT, Rudtanatip T, Soowannayan C, Nambunruang B, Medhe SV, Wongprasert K. Depolymerized Fractions of Sulfated Galactans Extracted from *Gracilaria fisheri* and Their Antibacterial Activity against *Vibrio parahaemolyticus* and *Vibrio harveyi*. Mar Drugs. 2022 Jul 23;20(8):469. doi: 10.3390/md20080469. <https://pubmed.ncbi.nlm.nih.gov/35892937/>

74.	Ghosh AK, Panda SK, Luyten W. Effectiveness of medicinal plant extracts against *Vibrio* spp. in shrimp aquaculture. Aquac Res. 2021;52: 6795–6801. doi:10.1111/are.15498. <https://onlinelibrary.wiley.com/doi/10.1111/are.15498>

75.	Ghosh AK, Panda SK, Luyten W. Anti-vibrio and immune-enhancing activity of medicinal plants in shrimp: A comprehensive review. Fish Shellfish Immunol. 2021;117: 192–210. doi:10.1016/j.fsi.2021.08.006.

<https://pubmed.ncbi.nlm.nih.gov/34400334/>

76.	Zheng X, Feyaerts AF, Van Dijck P, Bossier P. Inhibitory Activity of Essential Oils against *Vibrio campbellii* and *Vibrio parahaemolyticus*. Microorganisms. 2020;8: 1946. doi:10.3390/microorganisms8121946.

<https://pubmed.ncbi.nlm.nih.gov/33302532/>

77.	Mahfuzul Hoque MD, Bari ML, Inatsu Y, Juneja VK, Kawamoto S. Antibacterial Activity of Guava (*Psidium guajava* L.) and Neem (*Azadirachta indica* A. Juss.) Extracts Against Foodborne Pathogens and Spoilage Bacteria. Foodborne Pathog Dis. 2007;4: 481–488. doi:10.1089/fpd.2007.0040.

<https://pubmed.ncbi.nlm.nih.gov/18041957/>

78.	Dewi NR, Huang H-T, Wu Y-S, Liao Z-H, Lin Y-J, Lee P-T, et al. Guava (*Psidium guajava*) leaf extract enhances immunity, growth, and resistance against *Vibrio parahaemolyticus* in white shrimp *Penaeus vannamei*. Fish Shellfish Immunol. 2021;118: 1–10. doi:10.1016/j.fsi.2021.08.017.

<https://pubmed.ncbi.nlm.nih.gov/34418559/>

***Table 1 at page 29- This table should include*** ***Observed mass, Predicted mass, and Delta mass.***

**Authors' answers:** As well-quizzed by Reviewer 1, Table 1 could include information on Observed mass, Predicted mass, and Delta mass. However, it is important to note that the mass spectrometer we use is an LTQ-XL, so we do not have an exact mass (at best we can provide around 0.1 and in some cases 0.05). Under this condition is not useful to provide a predicted mass or even a delta if we focus on the rounded mass, rupture patterns, and some standard or previous information. The actual data presentation in Table 1 is not unusual and several publications followed this presentation format from our research group and even other research groups, such as the following examples:

Guevara M, **Tejera E**, Iturralde GA, Jaramillo-Vivanco T, Granda-Albuja MG, Granja-Albuja S, Santos-Buelga C, González-Paramás AM, Álvarez-Suarez JM. Anti-inflammatory effect of the medicinal herbal mixture infusion, Horchata, from southern Ecuador against LPS-induced cytotoxic damage in RAW 264.7 macrophages. Food Chem Toxicol. 2019 Sep;131:110594. doi: 10.1016/j.fct.2019.110594.

<https://doi.org/10.1016/j.fct.2019.110594>

Fernandez-Soto P, Celi D, **Tejera E**, Alvarez-Suarez JM, **Machado A**. *Cinnamomum* sp. and *Pelargonium odoratissimum* as the Main Contributors to the Antibacterial Activity of the Medicinal Drink Horchata: A Study Based on the Antibacterial and Chemical Analysis of 21 Plants. Molecules. 2023 Jan 10;28(2):693. doi: 10.3390/molecules28020693.

<https://doi.org/10.3390/molecules28020693>

Borges DGL, Echeverria JT, de Oliveira TL, Heckler RP, de Freitas MG, Damasceno-Junior GA, Carollo CA, Borges FA. Discovery of potential ovicidal natural products using metabolomics. PLoS One. 2019 Jan 25;14(1):e0211237. doi: 10.1371/journal.pone.0211237.

<https://doi.org/10.1371/journal.pone.0211237>

Shahzad MN, Ahmad S, Tousif MI, Ahmad I, Rao H, Ahmad B, Basit A. Profiling of phytochemicals from aerial parts of *Terminalia neotaliala* using LC-ESI-MS2 and determination of antioxidant and enzyme inhibition activities. PLoS One. 2022 Mar 31;17(3):e0266094. doi: 10.1371/journal.pone.0266094. <https://doi.org/10.1371/journal.pone.0266094>

**Authors' final comment:** We appreciated all the suggestions and efforts made by Reviewer 1 that really allowed us to improve the original manuscript. We hope that Reviewer 1 finds this version suitable for publication in the *Plos One* journal.

# **Reviewer 2**

***Comments to the Authors***

***Reviewer #2: The manuscript presents meticulously designed experiments with a logical structure, promising to capture the interest of the scientific community. The findings contribute significantly to the field.***

**Authors' answers:** We greatly appreciated your positive comments. Thank you!

***However, the reviewer highlighted areas for improvement. Firstly, the introduction should clearly articulate the main objective and underscore the novelty of the research. A comprehensive introduction will aid readers in understanding the study's significance.***

**Authors' answers:** We want to thank Reviewer 2 for his/her constructive comments and thoughtful suggestions that allowed us to improve the original manuscript. We are pleased to inform you that thanks to your recommendations we have made the following changes:

**Lines 87-90 on page 3 of the revised manuscript**

“Eucalyptus and Guava leaves constitute raw and cheap materials with well-known antimicrobial activity [9,10], which are not commonly used in the aquaculture industry, thus constituting a source of antimicrobial agents for a new potential sustainable economy for the Ecuadorian market.”

**Lines 114-123 on pages 3-4 of the revised manuscript**

“The main diseases that affect shrimp are the early mortality shrimp-acute hepatopancreatic necrosis disease (EMS – AHPND), which affects shrimp postlarvae within 20–30 days after stocking and frequently causes up to 100% mortality [21,22], and vibriosis which mortality can range between 70-100% but damages the shrimp´s oral cavity and appendages [5,23]. Therefore, several *Vibrio* species can significantly reduce shrimp production where *V. parahaemolyticus*, *V. harveyi*, *V. alginolyticus*, and *V. vulnificus* are commonly isolated in these shrimp farms [24]. In addition, certain *Vibrio* species can emerge as a serious threat to human health, such as *V. parahaemolyticus*, *V. cholerae*, and *V. alginolyticus* [25]. In Ecuador, the prevalence of *V. parahaemolyticus* and *V. cholerae* in shrimps at retail, farms, and estuaries is alarming and represents an eminent and serious public health risk [26,27].”

**Added references**

5.	Sanches-Fernandes GMM, Sá-Correia I, Costa R. Vibriosis Outbreaks in Aquaculture: Addressing Environmental and Public Health Concerns and Preventive Therapies Using Gilthead Seabream Farming as a Model System. Front Microbiol. 2022;13. doi:10.3389/fmicb.2022.904815

9.	Salvatori ES, Morgan L V., Ferrarini S, Zilli GAL, Rosina A, Almeida MOP, et al. Anti-Inflammatory and Antimicrobial Effects of *Eucalyptus* spp. Essential Oils: A Potential Valuable Use for an Industry Byproduct. Evidence-Based Complementary and Alternative Medicine. 2023;2023: 1–13. doi:10.1155/2023/2582698

10.	Díaz-de-Cerio E, Verardo V, Gómez-Caravaca A, Fernández-Gutiérrez A, Segura-Carretero A. Health Effects of *Psidium guajava* L. Leaves: An Overview of the Last Decade. Int J Mol Sci. 2017;18: 897. doi:10.3390/ijms18040897

23.	De La Peña LD, Cabillon NAR, Catedral DD, Amar EC, Usero RC, Monotilla WD, et al. Acute hepatopancreatic necrosis disease (AHPND) outbreaks in *Penaeus vannamei* and *P. monodon* cultured in the Philippines. Dis Aquat Organ. 2015;116: 251–254. doi:10.3354/dao02919

24.	de Souza Valente C, Wan AHL. Vibrio and major commercially important vibriosis diseases in decapod crustaceans. J Invertebr Pathol. 2021;181. doi:10.1016/j.jip.2020.107527

25.	Robert-Pillot A, Copin S, Himber C, Gay M, Quilici ML. Occurrence of the three major *Vibrio* species pathogenic for human in seafood products consumed in France using real-time PCR. Int J Food Microbiol. 2014;189: 75–81. doi:10.1016/j.ijfoodmicro.2014.07.014

26.	Ryan SJ, Stewart-Ibarra AM, Ordóñez-Enireb E, Chu W, Finkelstein JL, King CA, et al. Spatiotemporal variation in environmental *Vibrio cholerae* in an estuary in southern coastal Ecuador. Int J Environ Res Public Health. 2018;15. doi:10.3390/ijerph15030486

27.	Sperling L, Alter T, Huehn S. Prevalence and antimicrobial resistance of *Vibrio* spp. in retail and farm shrimps in Ecuador. J Food Prot. 2015;78: 2089–2092. doi:10.4315/0362-028X.JFP-15-160

***Secondly, there is a need for improvement in figure quality to enhance clarity and readability. High-quality figures are essential for facilitating comprehension, and efforts should be directed toward refining the visual representations.***

**Authors' answers:** As well-observed by Reviewer 2, the figure quality should be improved to enhance the clarity of the obtained results. We took the reviewer's advice and we followed the PLOS ONE recommendations to improve all figures resolution as indicated on the online website:

<https://journals.plos.org/plosone/s/figures#loc-resolution>

All figures were properly improved through Inkscape software following the desired dimensions with the 600-dpi resolution and then we used the PACE tool to validate or correct the improvements realized in the original figures.

<https://pacev2.apexcovantage.com/>

As you may see in the image above, all figures were corrected and approved by the PACE tool given by PLOS ONE guidelines. However, it is important to mention that, when uploading the figures on the journal online platform, the figures lose some resolution in the visualization in the pdf archive. Please consider this observation when evaluating the improvements made on the new figures.

Please check the resolution of the corrected and approved PACE tool figures by download the original pictures in the pdf archive as follow:

We hope that Reviewer 2 finds this version suitable for publication in the *Plos One* journal.

***Lastly, the discussion section requires attention. Some parts are challenging for readers to comprehend, so efforts should be made to enhance coherence and clarity, ensuring a better understanding for the audience.***

**Authors' answers:** As well-recommended by Reviewer 2, we carefully revised the Discussion section rectifying grammatical errors and confusing sentences. We also justified the selection of *Vibrio* species as well as Eucalyptus and Guava extracts in the Ecuadorian context enhancing the novelty of the present study (see lines 639-642 on page 16 of the revised manuscript). Furthermore, we added previous studies realized on *Vibrio* species with these plant extracts but clarified the lack of characterization in biofilm formation of *V.* *parahaemolyticus* and *V.* *cholerae* under different growth variables or the evaluation of cell viability following biomass assays (see lines 649-663 and 671-686 on page 17 of the revised manuscript). Finally, several limitations of the present study were acknowledged (see lines 732-738 on page 18 and 760-766 on page 19) and further recommendations were appointed for future research (see lines 767-779 on page 19 of the revised manuscript). Please see all these modifications and rectifications on pages 16-19 of the revised manuscript.

***Specific comments:***

***Comment 1: In line 171, 202, minutes~min. Please check throughout the manuscript.***

**Authors' answers:** As well-stated by Reviewer 2, the original manuscript showed the inconsistency of these two terms. We apologize for this mistake. We replaced all “minutes” with “min” in all text of the revised manuscript, please check the six amendments in lines 215, 236, 237, 239, 249, and 293 in the revised manuscript.

***Comment 2: In line 246-251, Please check, rewrite.***

**Authors' answers:** As recommended by Reviewer 2, we rewrote the sentences eliminating the repetitions and simplifying the description of the total cell counting. We apologize for the confusing sentences. Please check the rectifications in lines 295-298 on page 8 of the revised manuscript, more exactly:

Lines 246-251 in the original manuscript

“For more reproducible result presentation, cell counts were given per cm^2^. The number of *Vibrio* cells was counted from each field to obtain the average number of cells over the total area of the abiotic surface. Briefly, the coverslip area (4.84E + 8 µm^2^) was divided by the area of the photograph picture (12880 µm^2^) and the average of live and dead cells from microscopic fields was multiplied by the previous ratio, obtaining the total number of cells over the abiotic glass surface, as previously described [29].”

**Lines 295-298 on page 8 in the revised manuscript**

“The total cell counts were given per cm^2^ ± standard deviation (*N* cells/cm^2^ ± SD) by manually counting the number of *Vibrio* cells from each field (12,880 µm^2^) and recalculating the average number of cells over the total coverslip area (4.84E+8 µm^2^), as previously described [40].”

***Comment 3: In line 466-470, This are not correct, please check.***

**Authors' answers:** As well-detected by Reviewer 2, the sentence was incorrect, and we apologize for this mistake. We checked the sentences and data in Supplementary Table S5, and so the sentences were rectified accordingly. Please check the amendments in lines 512-517 on pages 13-14 of the revised manuscript, more exactly:

Lines 466-470 in the original manuscript

“Over time *V. parahaemolyticus* showed a higher number of dead cells within the biofilm (around 44%) when compared to *V. cholerae* (*p* <0.001). Although no statistical differences were found between *Vibrio* species in the total count and live cells, both biofilms evidenced significant differences in their intrinsic growth between 24 and 48 h (*p* <0.001), as well as 48 and 72 h (*p* <0.001).”

**Lines 512-517 on pages 13-14 in the revised manuscript**

“More exactly, *V. parahaemolyticus* showed a higher number of dead cells within the biofilm (9.09E+5 dead cells/cm^2^) and approximately 21% of the total cells, while *V. cholerae* only showed 13% of dead cells (5.05E+5 dead cells/cm^2^) within the biofilm (*p* <0.001). Although no statistical differences were found between *Vibrio* species in the total count and live cells, both *Vibrio* biofilms evidenced significant differences in their intrinsic growth between 24 and 48 h (*p* <0.001), as well as 48 and 72 h (*p* <0.001).”

**Authors' final comment:** We appreciated all the suggestions and efforts made by Reviewer 2 that really allowed us to improve the original manuscript. We hope that Reviewer 2 finds this version suitable for publication in the *Plos One* journal.

# **Reviewer 3**

***Comments to the Author***

**Authors' answers:** We want to thank Reviewer 3 for his/her constructive comments and thoughtful suggestions that allowed us to improve the original manuscript. All unclear aspects were addressed in the revised manuscript. We are pleased to inform you that thanks to your recommendations we have made the following changes:

***Reviewer #3: This MS is well written but lacks many scientific aspects. Scientifically the major issue on this MS is it was not followed up through bioassay guided purification. The compounds are identified by MS based on the major abundance is inappropriate since it brings lot of questions. What compound is responsible for the bioactivity. Authors did not test any of the predicted compounds leads to novelty, since previously reports are available on these two plants with against Vibrio parahaemolyticus.***

**Authors' answers:** As well-observed by Reviewer 3, it is a very important topic, and we agree with the reviewer that several questions remain unanswered. We don’t have (now) the pure compounds to validate possible molecules responsible for biological activity. Actually, we are currently working on fractioning the sample in order to narrow the possible chemicals responsible for biological activity while at the same time getting more information about other molecules less abundant in the extract and that we cannot reject their involvement in the activity. We can´t say which molecule is responsible and probably more than one molecule is involved in the biological response. These limitations were included in the manuscript in order to be clear on the scope of the work and to open further research analysis. Please check these limitations at the end of the Discussion section in lines 767-773 on page 19 of the revised manuscript, more exactly:

“Limitations and further recommendations

In the present work, we indicate the biological activity of the Eucalyptus (*Eucalyptus globulus*) and Guava (*Psidium guajava*) leaf extracts. However, we don’t know which of the identified compounds is responsible for the activity. Further studies need to be done to fraction the extracts with further activity evaluation and/or the direct biological activity evaluation of the pure identified compounds. Moreover, future experiments could be designed to determine the mechanism behind biological activity.”

***Guava (Psidium guajava) leaf extract enhances immunity, growth, and resistance against Vibrio parahaemolyticus in white shrimp Penaeus vannamei - PubMed (***[***nih.gov***](http://nih.gov/)***)***

***Antibacterial activity of guava (Psidium guajava L.) and Neem (Azadirachta indica A. Juss.) extracts against foodborne pathogens and spoilage bacteria - PubMed (***[***nih.gov***](http://nih.gov/)***)***

***Microorganisms | Free Full-Text | Inhibitory Activity of Essential Oils against Vibrio campbellii and Vibrio parahaemolyticus (***[***mdpi.com***](http://mdpi.com/)***)***

***Anti-vibrio and immune-enhancing activity of medicinal plants in shrimp: A comprehensive review - ScienceDirect
Effectiveness of medicinal plant extracts against Vibrio spp. in shrimp aquaculture - Ghosh - 2021 - Aquaculture Research - Wiley Online Library
While w.r.t. V. cholerae so many literatures available even with antibiofilm properties.***

**Authors' answers:** As well-appointed by Reviewer 3, we discussed previous studies with alternative strategies to control *Vibrio*-related infections, more exactly Yatip et al. (2018), Karnjana et al. (2019), and Kamble et al. (2022), justifying the selection of Eucalyptus and Guayaba due to their well-known antibacterial activity by other previous studies (Gosh et al., 2021a and 2021b; Zheng et al., 2020; Dewi et al., 2021; Mahfuzul Hoque et al., 2007) and their abundance in Ecuador to be applied in the shrimp farms. Please check this new discussion in lines 672-687 on page 17 of the revised manuscript, more exactly:

“Several studies have been conducted with alternative treatments to control *Vibrio*-associated infections using probiotic and natural-derived products. In Thailand, Yatip and colleagues applied extract from the fermented soybean product Natto to inhibit *Vibrio* biofilm formation [29], while Karnjana and colleagues evaluated the ethanolic extract of the red seaweed *G. fisheri* suggesting its future application as an alternative treatment for biofilm-associated *Vibrio* infections in aquacultures [30]. While Kamble and colleagues assessed the antimicrobial activity of sulphated galactans of *G. fisheri* against *V. parahaemolyticus* and *V. harveyi* [31]. All these studies analyzed abundant and native natural products of Thailand's resources for their application in the shrimp industry. Likewise, Ecuador evidenced abundant and natural products with well-known antimicrobial activity such as Eucalyptus (*Eucalyptus globulus*) and Guava (*Psidium guajava*) [73–77], which evaluated the antimicrobial activity of Eucalyptus and Guayaba extracts against several *Vibrio* species (including *V. parahaemolyticus*). However, to the authors’ best acknowledge, none of the previous studies characterized biofilm formation of *V.* *parahaemolyticus* and *V.* *cholerae* under different growth variables nor evaluated cell viability after biomass assays. As demonstrated in this study, the cell viability reduction was significantly higher when compared to biomass assays and antibiotic activity, thus illustrating the novelty of the present study. Therefore,”

Although several previous studies indeed evaluated the antimicrobial activity of Eucalyptus (*Eucalyptus globulus*) and Guava (*Psidium guajava*) extracts against *Vibrio* species (as well-stated by Reviewer 3), none of the previous literature to the best acknowledge of the authors realized an extensive evaluation of these *Vibrio* species' biofilm formation under different growth variables nor evaluated cell viability after biomass assays. As demonstrated by the present work, the cell viability reduction was significantly efficient when compared to biomass assays and antibiotic activity, thus justifying the unique results of the present study as a novelty. However, as well-observed by Reviewer 3, the lack of evaluation of the predicted compounds constitutes by itself a strong limitation that was acknowledged at the end of the Discussion section. Future studies will evaluate the fractions or purified compounds of these extracts but, until the present moment, we did not have the financial funding to proceed without the publication of the extensive work already realized and we are currently optimizing the fraction procedure in the laboratory. We hope that Reviewer 3 understands our justification and present situation.

**Added references**

29. Yatip P, Nitin Chandra Teja D, Flegel TW, Soowannayan C. Extract from the fermented soybean product Natto inhibits *Vibrio* biofilm formation and reduces shrimp mortality from *Vibrio harveyi* infection. Fish Shellfish Immunol. 2018 Jan;72:348-355. doi: 10.1016/j.fsi.2017.11.008. <https://pubmed.ncbi.nlm.nih.gov/29127029/>

30. Karnjana K, Soowannayan C, Wongprasert K. Ethanolic extract of red seaweed *Gracilaria fisheri* and furanone eradicate *Vibrio harveyi* and *Vibrio parahaemolyticus* biofilms and ameliorate the bacterial infection in shrimp. Fish Shellfish Immunol. 2019 May;88:91-101. doi: 10.1016/j.fsi.2019.01.058. <https://pubmed.ncbi.nlm.nih.gov/30817993/>

31. Kamble MT, Rudtanatip T, Soowannayan C, Nambunruang B, Medhe SV, Wongprasert K. Depolymerized Fractions of Sulfated Galactans Extracted from *Gracilaria fisheri* and Their Antibacterial Activity against *Vibrio parahaemolyticus* and *Vibrio harveyi*. Mar Drugs. 2022 Jul 23;20(8):469. doi: 10.3390/md20080469. <https://pubmed.ncbi.nlm.nih.gov/35892937/>

73.	Ghosh AK, Panda SK, Luyten W. Effectiveness of medicinal plant extracts against *Vibrio* spp. in shrimp aquaculture. Aquac Res. 2021;52: 6795–6801. doi:10.1111/are.15498. <https://onlinelibrary.wiley.com/doi/10.1111/are.15498>

74.	Ghosh AK, Panda SK, Luyten W. Anti-vibrio and immune-enhancing activity of medicinal plants in shrimp: A comprehensive review. Fish Shellfish Immunol. 2021;117: 192–210. doi:10.1016/j.fsi.2021.08.006.

<https://pubmed.ncbi.nlm.nih.gov/34400334/>

75.	Zheng X, Feyaerts AF, Van Dijck P, Bossier P. Inhibitory Activity of Essential Oils against *Vibrio campbellii* and *Vibrio parahaemolyticus*. Microorganisms. 2020;8: 1946. doi:10.3390/microorganisms8121946.

<https://pubmed.ncbi.nlm.nih.gov/33302532/>

76.	Mahfuzul Hoque MD, Bari ML, Inatsu Y, Juneja VK, Kawamoto S. Antibacterial Activity of Guava (*Psidium guajava* L.) and Neem (*Azadirachta indica* A. Juss.) Extracts Against Foodborne Pathogens and Spoilage Bacteria. Foodborne Pathog Dis. 2007;4: 481–488. doi:10.1089/fpd.2007.0040.

<https://pubmed.ncbi.nlm.nih.gov/18041957/>

77.	Dewi NR, Huang H-T, Wu Y-S, Liao Z-H, Lin Y-J, Lee P-T, et al. Guava (*Psidium guajava*) leaf extract enhances immunity, growth, and resistance against *Vibrio parahaemolyticus* in white shrimp *Penaeus vannamei*. Fish Shellfish Immunol. 2021;118: 1–10. doi:10.1016/j.fsi.2021.08.017.

<https://pubmed.ncbi.nlm.nih.gov/34418559/>

***Other comments:***

***1. here are several grammatical errors throughout the manuscript. I recommend having a native English speaker review the manuscript for clarity and correctness.***

**Authors' answers:** As well-recommended by Reviewer 3, we realized intensive English editing together with English native speakers of our institution to improve the manuscript (Please check these amendments on the text of the revised manuscript with track changes).

***2. This MS lack the detail information on indigenous uses in aquaculture or why these plants were chosen. Providing this context could strengthen your argument and make the study more relatable to readers.***

**Authors' answers:** As well-detected by Reviewer 3, we indicated the reason why these plant extracts and *Vibrio* species were chosen in the present study. Please check the Introduction section of the revised manuscript, more exactly:

**Lines 87-90 on page 3 of the revised manuscript**

“Eucalyptus and Guava leaves constitute raw and cheap materials with well-known antimicrobial activity [9,10], which are not commonly used in the aquaculture industry, thus constituting a source of antimicrobial agents for a new potential sustainable economy for the Ecuadorian market.”

**Lines 119-123 on pages 3-4 of the revised manuscript**

“In addition, certain *Vibrio* species can emerge as a serious threat to human health, such as *V. parahaemolyticus*, *V. cholerae*, and *V. alginolyticus* [25]. In Ecuador, the prevalence of *V. parahaemolyticus* and *V. cholerae* in shrimps at retail, farms, and estuaries is alarming and represents an eminent and serious public health risk [26,27].”

***3. The taxonomic identification of the plants is missing, and no voucher specimen appears to have been deposited. This is essential for the reproducibility and verification of your study. Why the plant is not identified to species level?***

**Authors' answers:** As well-detected by Reviewer 3, the taxonomic identification of the plants and voucher specimens were missing in the original manuscript. We apologized for these lapses and we reached Professor Nelson Miranda, who is the curator at the Herbario de Botánica Económica del Ecuador of the Universidad San Francisco de Quito USFQ, for the proper taxonomic identification of the plant leaves. The voucher specimens of Guava (*Psidium guajava* L.) and Eucalyptus (*Eucalyptus globulus* Labill.) leaf samples used in this study are deposited at Herbario de Botánica Económica del Ecuador of the USFQ under the codes #35004 and #35005, respectively. Finally, we properly added all this information to the revised manuscript. Please check the amendments in lines 178-181 and 207-212 on pages 5-6 of the revised manuscript, more exactly:

**Lines 178-181 on page 5 of the revised manuscript**

“Fresh guava (*Psidium guajava*) leaf samples were obtained from the Sangolquí market, province of Pichincha, Ecuador. Eucalyptus (*Eucalyptus globulus*) leaf samples were collected at Alangasí, province of Pichincha, Ecuador. All samples were identified by Nelson Miranda, curator at the Herbario de Botánica Económica del Ecuador of the Universidad San Francisco de Quito USFQ.”

**Lines 207-212 on page 6 of the revised manuscript**

“**2.3 Ethics statement**

Voucher specimens of Guava (*Psidium guajava*) and Eucalyptus (*Eucalyptus globulus*) leaf samples used in this study are deposited at Herbario de Botánica Económica del Ecuador of the USFQ under the codes #35004 and #35005, respectively. The present study was conducted under the authorization for collection of biological specimens (MAATE-ARSFC-2024-0328) issued by the Ministry of Environment, Water and Ecological Transition of Ecuador MAATE.”

***4. What are the yield value of the extract.***

**Authors' answers:** As well-noticed by Reviewer 3, we did not report the yield values of the plant extracts. We apologize for this lapse and thank you for calling our attention to this mistake. We properly add the calculation of the yield values in subsection 2.2 (lines 184-188 on page 5) and the yield results in subsection 3.1 (lines 419-421 on page 11) of the revised manuscript, more exactly:

**Lines 184-188 on page 5 of the revised manuscript**

“Percent yield values of both plant extracts were calculated using the following formula:

$Yield \left( \% \right)= \frac{Dried extract weight (g)}{Biomass weight (g)}\times100.$

For every plant extract, triplicate leaf samples were used for extraction and yield values of the extracts were expressed as mean percentage ± standard deviation (SD) (n = 3).”

**Lines 419-421 on page 11 of the revised manuscript**

“In addition, Guava (*Psidium guajava*) and Eucalyptus (*Eucalyptus globulus*) showed yield values in the extracts of 19.6% (± 0.6) and 19.7% (± 0.4), respectively.”

Thank you for calling our attention and allowing us to improve the original version of the manuscript!

***5. Line nos : 169-186, deals with biofilm but the cited reference is for Candida sp. This raises questions about the authenticity of the protocols followed. Please ensure that the references cited are relevant to your study.
21. “Atiencia-Carrera MB, Cabezas-Mera FS, Vizuete K, Debut A, Tejera E, Machado A. Evaluation 788 of the biofilm life cycle between Candida albicans and Candida tropicalis. Front Cell Infect 789 Microbiol. 2022;12: 953168. doi:10.3389/fcimb.2022.953168” and 24. “Lohse MB, Gulati M, Johnson AD, Nobile CJ. Development and regulation of single-and multi 798 species Candida albicans biofilms. Nat Rev Microbiol. 2018;16: 19–31. 799 doi:10.1038/NRMICRO.2017.107”***

**Authors' answers:** As well-detected by Reviewer 3, the references cited in protocols for biofilm assays are related to *Candida* biofilms. However, it is important to mention that protocols for biofilm optimization and characterization can be applied to different microorganisms, and our research group specializes in biofilm characterization as Reviewer 3 may check in some of our most recent papers:

Cabezas-Mera FS, Atiencia-Carrera MB, Villacrés-Granda I, Proaño AA, Debut A, Vizuete K, Herrero-Bayo L, Gonzalez-Paramás AM, Giampieri F, Abreu-Naranjo R, **Tejera E**, Álvarez-Suarez JM, **Machado A**. Evaluation of the polyphenolic profile of native Ecuadorian stingless bee honeys (Tribe: Meliponini) and their antibiofilm activity on susceptible and multidrug-resistant pathogens: An exploratory analysis. Curr Res Food Sci. 2023 Jun 29;7:100543. doi: 10.1016/j.crfs.2023.100543. <https://www.ncbi.nlm.nih.gov/pmc/articles/PMC10344713/>

Cangui-Panchi SP, Ñacato-Toapanta AL, Enríquez-Martínez LJ, Salinas-Delgado GA, Reyes J, Garzon-Chavez D, **Machado A.** Battle royale: Immune response on biofilms - host-pathogen interactions. Curr Res Immunol. 2023 Mar 28;4:100057. doi: 10.1016/j.crimmu.2023.100057.

<https://pubmed.ncbi.nlm.nih.gov/37025390/>

Cangui-Panchi SP, Ñacato-Toapanta AL, Enríquez-Martínez LJ, Reyes J, Garzon-Chavez D, **Machado A**. Biofilm-forming microorganisms causing hospital-acquired infections from intravenous catheter: A systematic review. Curr Res Microb Sci. 2022 Nov 17;3:100175. doi: 10.1016/j.crmicr.2022.100175.

<https://pubmed.ncbi.nlm.nih.gov/36518176/>

**Machado A**, Foschi C, Marangoni A. Editorial: Vaginal dysbiosis and biofilms. Front Cell Infect Microbiol. 2022 Aug 9;12:976057. doi: 10.3389/fcimb.2022.976057.

<https://pubmed.ncbi.nlm.nih.gov/36017371/>

Atiencia-Carrera MB, Cabezas-Mera FS, Vizuete K, Debut A, **Tejera E, Machado A**. Evaluation of the biofilm life cycle between *Candida albicans* and *Candida tropicalis*. Front Cell Infect Microbiol. 2022 Aug 18;12:953168. doi: 10.3389/fcimb.2022.953168.

<https://pubmed.ncbi.nlm.nih.gov/36061861/>

Nonetheless, to ensure that the references cited are relevant to the study, we replaced the previous reference of Lohse et al. (2018) with our last study realized by Cabezas-Mera et al. (2023), which the biofilm assays of the present study were realized in 6-well plates containing a sterile coverslip (as abiotic surface) as described in the subsection 2.4.2. of our most recent published study of 2023 (<https://doi.org/10.1016/j.crfs.2023.100543>). Also, the biofilm assays realized in Cabezas-Mera et al. (2023) were applied against different microorganisms’ biofilms, more exactly *S. aureus*, *K. pneumoniae*, *C. albicans*, and *C. tropicalis*. Please check the replaced citation [35] in line 222 on page 6 of the revised manuscript.

**Replace reference**

35.	Cabezas-Mera FS, Atiencia-Carrera MB, Villacrés-Granda I, Proaño AA, Debut A, Vizuete K, et al. Evaluation of the polyphenolic profile of native Ecuadorian stingless bee honeys (Tribe: Meliponini) and their antibiofilm activity on susceptible and multidrug-resistant pathogens: An exploratory analysis. Curr Res Food Sci. 2023;7: 100543. doi:10.1016/j.crfs.2023.100543

***“2.4.1 Crystal violet staining” is well known but lacks here with proper citation.***

**Authors' answers:** As well-appointed by Reviewer 3, the now subsection 2.5.1 lacked proper citation and we apologize for this lapse. We properly cited a previous reference study using crystal violet staining protocol, where the authors compared multiple methods for quantification of microbial biofilms by biomass assays. Please check the citations in lines 232-233 on page 6 in the subsection 2.5.1 of the revised manuscript, more exactly:

**Lines 232-233 on page 6 in the subsection 2.5.1 of the revised manuscript**

“After a certain period of growth (24, 48, and 72 h), we used the CV method to quantify the biomass of biofilm samples as described by Peeters and colleagues [36] with slight modifications. Briefly, …”

**Added reference**

36.	Peeters E, Nelis HJ, Coenye T. Comparison of multiple methods for quantification of microbial biofilms grown in microtiter plates. J Microbiol Methods. 2008;72: 157–165. doi:10.1016/J.MIMET.2007.11.010

***6. Line no. 154 “0.5 mL of 0.1 % formic acid/acetonitrile (70:30, v/v), does it mean 30% water? On line 154, you mention a 0.1% formic acid/acetonitrile (70:30, v/v) solution. If this implies 30% water, a water extract would be expected to dissolve in 1-5% acetonitrile rather than 70% acetonitrile. Please provide details of the HPLC LC protocols and chromatogram as supplementary information.***

**Authors' answers:** As requested by Reviewer 3, we checked the details of the HPLC protocol. There was an error in the description of the original manuscript, and we apologize for this mistake. We rectified the HPLC description in the revised manuscript. We used a mixture of methanol/water (80:20 v/v) as previously used in our recent study of 2023 (Please check: <https://www.ncbi.nlm.nih.gov/pmc/articles/PMC9862262/>). This description is now corrected in the new version of the manuscript. The reason to use this strategy is two-fold: 1) The addition of methanol in high concentration improves the solubility of some compounds that when concentration increased tend to reduce their solubility and 2) We had been using this strategy (with the same gradient elution) in different samples and standards which facilitate identification comparing mass ruptures and retention time.

In the manuscript we also added the elution gradient and in the supplementary materials the chromatograms (see the new Supplementary Figure S1).

**The rectified HPLC protocol description in lines 189-199 on page 5 of the revised manuscript**

“The dry extracts were dissolved in 1 mL of methanol/water (80:20, v/v), filtered through a 0.22 μm disposable LC filter disk (RephiLe Bioscience Ltd), and analyzed with HPLC using double online detection by diode array detector (DAD) and mass spectrometry (MS) following a previously described methodology [34]. The system consisted of a Vanquish (Thermo Fisher Scientific) fitted with a binary pump and DAD coupled with an LTQ-XL (Thermo Fisher Scientific) controlled by Xcalibur Software (Thermo Scientific™, Waltham, Massachusetts, USA). An Accucore Vanquish C18 column (1.5 µm, 100 × 2.1 mm) thermostated at 35 °C was used as the stationary phase, while the mobile phase consisted of a solution of 0.1% formic acid (A) and acetonitrile (B). The elution gradient established was: 2% B, 0–4 min; 4% B, 4–22 min; 40% B, 22–32 min; 70% B, 32–35 min; 98% B, 35–40 min, and rebalanced the column to the initial conditions of the solvent. The flow rate was 0.2 mL/min, and the injection volume was 50 µL (see the descriptive chromatograms in Supplementary Figure S1).”

**Added reference**

34.	Fernandez-Soto P, Celi D, Tejera E, Alvarez-Suarez JM, Machado A. *Cinnamomum* sp. and *Pelargonium odoratissimum* as the Main Contributors to the Antibacterial Activity of the Medicinal Drink Horchata: A Study Based on the Antibacterial and Chemical Analysis of 21 Plants. Molecules. 2023;28. doi:10.3390/molecules28020693

The new Supplementary Figure S1 of the chromatograms can be observed in the revised supplementary material (please check lines 2-10 on page 1 of the supplementary file), as follows:

**Supplementary Figure S1.** Descriptive chromatograms obtained in Eucalyptus (*Eucalyptus globulus*) and Guava (*Psidium guajava*) extracts during HPLC-DAD-MS analysis.

***7. The figures in the manuscript are of low quality and difficult to read. Please consider improving the resolution for better readability.***

**Authors' answers:** As well-observed by Reviewer 3, the figure quality should be improved to enhance the clarity of the obtained results. We took the reviewer's advice, and we followed the PLOS ONE recommendations to improve all figures resolution as indicated on the online website:

<https://journals.plos.org/plosone/s/figures#loc-resolution>

All figures were properly improved through Inkscape software following the desired dimensions with the 600-dpi resolution and then we used the PACE tool to correct and/or validate the improvements realized in the original figures.

<https://pacev2.apexcovantage.com/>

As you may see in the image above, all figures were corrected and approved by the PACE tool given by PLOS ONE guidelines. However, it is important to mention that, when uploading the figures on the journal online platform, the figures lose some resolution in the visualization of the pdf archive. Please consider this observation when evaluating the improvements made on the new figures.

Please check the resolution of the corrected and approved PACE tool figures by download the original pictures in the pdf archive as follow:

We hope that Reviewer 3 finds this version suitable for publication in the *Plos One* journal.

***8. The plant name is not italics in many cases, and authors citation is italics. Many time it is abbreviated and again full name is mentioned.***

**Authors' answers:** As well-detected by Reviewer 3, in many cases the plant names were not mentioned in scientific format or italic form, as well as some authors' citations are wrongly cited in italic form. In addition, some bacterial names were fully mentioned after the abbreviation was added. We apologize and we rectified all these errors during the English editing with the English native speakers of our institution, as recommended by Reviewer 3. Thank you!

***9. References are not uniform format. Many biological names are not italics.***

**Authors' answers:** As well-noted by Reviewer 3, we rectified all the references of the revised manuscript, and we put all species names in italic form. However, it is important to mention that the reference software did not insert the species name in the italic form. We believe that an incorrect reference software update occurred during the submission time. We also checked for duplicated references and reference-associated errors in the remaining text of the revised manuscript. We apologize for these errors. Thank you for correcting us.

**Authors' final comment:** We appreciated all the suggestions and efforts made by Reviewer 3 that really allowed us to improve the original manuscript. We hope that Reviewer 3 finds this version suitable for publication in the *Plos One* journal.
